# The synergistic extract of *Zophobas atratus* and *Tenebrio molitor* regulates neuroplasticity and oxidative stress in a scopolamine-induced cognitive impairment model

**DOI:** 10.3389/fnagi.2025.1566621

**Published:** 2025-04-23

**Authors:** Ngoc Buu Tran, Haesung Lee, Myoung-Geun Ji, Long Ngo Hoang, Sook-Jeong Lee

**Affiliations:** Department of Bioactive Material Sciences and Research Center of Bioactive Materials, Jeonbuk National University, Jeonju, Jeonbuk-do, Republic of Korea

**Keywords:** cognitive dysfunction, edible insect, neuroinflammation, oxidative stress, senescence

## Abstract

**Introduction:**

Neurodegenerative disorders, such as Alzheimer’s disease, arise from neuroinflammation, which leads to cognitive and memory impairment. Scopolamine is commonly used to induce cognitive and memory deficits in mouse models.

**Aims:**

This study investigated the neuroprotective potential of a *Zophobas atratus* (*Za*) and *Tenebrio molitor* (Tm) extract mixture (ZaTm mixture) in mitigating scopolamine-induced cognitive and memory deficits in mice.

**Results:**

Behavioral assessments, including the Morris water maze, Y-maze, and light/dark tests, demonstrated that the ZaTm mixture significantly enhanced memory and cognitive function in treated mice. Furthermore, the ZaTm mixture restored the disrupted expression of choline acetyltransferase and acetylcholinesterase in the hippocampi of scopolamine-treated mice. Additionally, scopolamine-induced glutamatergic/GABAergic dysfunction was markedly improved following treatment with the ZaTm mixture. The extract also exhibited neuroprotective effects by enhancing the activity of antioxidants, such as glutathione and malondialdehyde, and key enzymes, including catalase and superoxide dismutase. Moreover, it effectively inhibited senescence in the hippocampus by modulating the AMPK/SIRT and BDNF-Akt/mTOR signaling pathways.

**Discussion:**

This study highlights the promising potential of the ZaTm extract mixture as a novel therapeutic agent and functional food for the prevention and treatment of Alzheimer’s disease and other neurodegenerative disorders.

## Graphical abstract

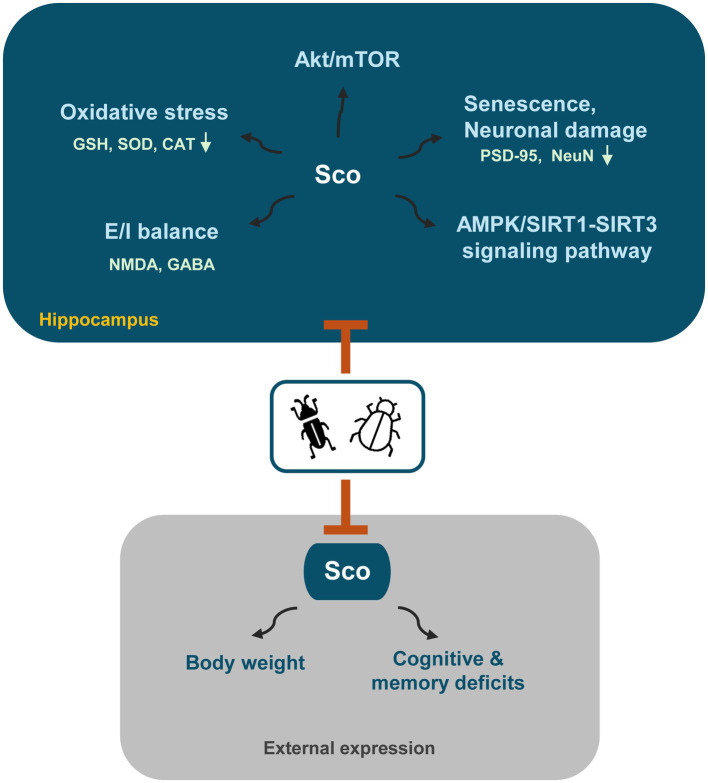

## 1 Introduction

As the average global lifespan continues to increase, the prevalence of neurodegenerative diseases, such as Alzheimer’s (AD) and Parkinson’s disease (PD), along with age-related cognitive and memory impairments, is also increasing (Agnihotri and Aruoma, 2020). Patients with AD often experience progression from early memory impairment to more complex functional disorders, including language deficits, dementia, visuospatial impairment, and difficulties in behavioral control (Huang et al., 2023). Nowadays, the evaluation of pharmacological targets in Alzheimer’s disease and its management as well as finding new approaches to treat many other neurological disorders have been widely studied such as the use of neurofilaments as markers in the management of neurological disorders (Tripathi et al., 2024; Sharma et al., 2024; Siddiqui et al., 2024). Recent clinical studies have indicated that treating the early stages of cognitive and motor impairments is the most effective way to delay the onset and progression of AD. Therefore, identifying the functional factors that enhance memory and behavior is substantially important (Bachurin et al., 2018). Restoring cholinergic function plays a crucial role in the management of AD symptoms. One approach to enhance cholinergic function in AD is to prolong the availability of acetylcholine (ACh) in the synaptic cleft. This can be achieved by inhibiting acetylcholinesterase (AChE), the enzyme responsible for ACh hydrolysis, using AChE inhibitors (Ferreira-Vieira et al., 2016). Disrupting the cholinergic system in the hippocampus lowers ACh and choline acetyltransferase (ChAT) levels, while increasing AChE levels, which accelerates choline ester hydrolysis, leading to memory loss and behavioral issues (Sugisaki et al., 2016).

Scopolamine, a muscarinic ACh receptor inhibitor, is commonly used to induce oxidative stress, reduce hippocampal volume, and disrupt cholinergic neurotransmission in rodents and humans (Khurana et al., 2021). Additionally, scopolamine lowers the expression of brain-derived neurotrophic factor (BDNF), a key neurotrophic factor that activates the BDNF receptor, TrkB, thereby enhancing synaptic plasticity, memory formation, and long-term memory retention. Previous studies have also shown that scopolamine disrupts the AMPK/SIRT1-SIRT3 signaling pathway, which is closely associated with the Akt/mTOR pathway, further contributing to cognitive deficits (Liu et al., 2024; Abuelezz and Hendawy, 2023). Disruptions in these signaling pathways lead to oxidative stress and hippocampal damage, and are key factors in neuronal aging, resulting in neurodegenerative diseases and impairments in motor function, cognition, and memory (Chen et al., 2012). Furthermore, abnormalities in synaptic proteins can impair neurotransmission at the excitatory (E) and inhibitory (I) synapses, disrupting the E/I balance and affecting neural plasticity. This imbalance, which can be triggered by disruptions in glutamatergic and GABAergic neuronal differentiation, may be the underlying cause of cognitive and memory deficits (Voleti et al., 2013).

Insect extracts exhibit protective effects against intestinal inflammation and liver damage, their ability to reduce systemic inflammation, and anticancer properties (Hwang et al., 2019; Stull et al., 2018; Lim and Byun, 2021). Several recent studies have shown that extracts from various insects have neuroprotective effects. For example, extract derived from insects of several species such as *Gryllus bimaculatus*, *Oxya chinensis sinuosa* effectively protects the blood–brain barrier, apoptosis, mitigates brain damage in models of autism, epilepsy, and enhance cognitive and motor skills (Tran and Lee, 2023; Tran et al., 2024; Phokasem et al., 2020). Bioactive peptides from insect-derived extracts as multifunctional molecules tailored for Alzheimer’s disease and neuroprotection (Bhola et al., 2024; Mesias et al., 2025). Besides, fermented *Protaetia brevitarsis larvae* improves neurotoxicity in chronic ethanol-induced-dementia mice via suppressing AKT and NF-κB signaling pathway (Lee et al., 2020). Furthermore, protective effects on neuronal SH-SY5Y cells and antioxidant activity of enzymatic hydrolyzate from Silkworms Fed the leaves of *Cudrania tricuspidata* was also reported (An et al., 2024).

*Tenebrio molitor* (Tm) is an excellent energy source owing to its high fat content (37.42–48.97% dry weight). Protein extracts (0.077–0.097 mg/mL) of Tm have lower blood pressure by inhibiting anti-ogensin-converting enzymes, anti-proliferative effects in human colorectal adenocarcinoma and hepatocellular carcinoma cell lines (Cito et al., 2017; Ding et al., 2021). A previous study has shown protective effects of *Tenebrio molitor* extracts in crossing and modulating blood–brain barrier, amyloid β plaques, and intestinal inflammation in mice with cognitive, memory, and behavioral deficits due to degeneration (Tran et al., 2024). *Zophobas atratus* (Za) is recognized for its anti-inflammatory and antibacterial properties, and is considered a novel food source (Kim et al., 2021). A recent study identified a glycine-rich peptide from Za, coleoptericin B, which targets bacterial membranes and offers protection against *Klebsiella pneumonia*-induced mastitis in mice (Wang et al., 2024). In addition, the neuroprotective effects of *Zophobas atratus* in improving cognition and behavior as well as ameliorating neuronal damage and modulating acetylcholinesterase activity in a scopolamine induced model of cognitive and behavioral deficits have been reported (Lee, 2024).

Previously, we have examined the neuroprotective effect of Za and Tm in different mouse disease such as autism spectrum disorder and aging. Despite the well-documented nutritional and health benefits of Za and Tm, studies with combined treatment of insect extracts are rare and their synergistic effects on neurodegeneration and cognitive impairment remain unexplored. This study aims to clarify the protective effects of these edible insect extracts against oxidative stress-induced neuronal toxicity, aging processes, and their ability to regulate the glutamatergic/GABAergic system to maintain the E/I balance, as well as their potential to modulate the AMPK/SIRT and BDNF-Akt/mTOR signaling pathways.

## 2 Materials and methods

### 2.1 Chemicals and antibodies

Supplementary Table S1 presents a comprehensive overview of the chemicals and antibodies employed in this study.

### 2.2 Preparation of ZaTm extract and analysis of mixture compounds

#### 2.2.1 Extract of ZaTm mixture

Dried Za and Tm were extracted using 10 volumes of distilled water at 60°C for 12 h. The Za and Tm extracts were filtered through progressively smaller strainers (90, 45, 40 μm), and finally through a 20-μm syringe filter to eliminate most bacteria, mold, and impurities (filter efficiency >99%). The extracts were then concentrated under reduced pressure using a rotary evaporator (IKA™ RV 3 V Rotary Evaporator; Thermo Fisher Scientific, Waltham, MA, USA) and freeze-dried. The prepared samples were stored at 4°C for future use. The mixing ratio of the Za and Tm insect powders followed our previously established protocol (Tran et al., 2023; Tran and Lee, 2023; Tran et al., 2024).

#### 2.2.2 Analysis of chromatography and mass spectrometry

LC–MS/MS (liquid chromatography-mass spectrometry) analysis was performed using an ultra-performance liquid chromatography (UPLC) system (Waters, Milford, USA) installed in the Center for University-wide Research Facilities (CURF) at Jeonbuk National University. The chromatographic separation was carried out using an ACQUITY UPLC HSS T3 column (100 mm × 2.1 mm, 1.8 μm, Waters) with a column temperature of 40°C and a flow rate of 0.5 mL/min, where the mobile phase contained solvent A (water +0.1% formic acid) and solvent B (acetonitrile +0.1% formic acid). Compounds were eluted using the following gradient elution conditions: 97% phase A for 0–5 min; 3–100% liner gradient phase B for 5 ~ 16 min; 100% phase B for 16–17 min; 100–3% reverse liner gradient phase B for 17 ~ 19 min; 97% Phase A for 19–25 min. The loading volume of each sample was 5 μL. The compounds eluted from the column were detected by a high-resolution tandem mass spectrometer SYNAPT G2 Si HDMS QTOF (Waters) in positive and negative ion modes. For positive ion mode, the capillary voltage and the cone voltage were set at 2 kV and 40 V, respectively. For negative ion mode, they were 1 kV and 40 V, respectively. Centroid MS^E^ mode was used to collect the mass spectrometry data. The primary scan ranged from 50 to 1,200 Da and the scanning time was 0.2 s. All the parent ions were fragmented using 20–40 eV. The information of all fragments were collected and the time was 0.2 s. In the data acquisition process, the LE signal was gained every 3 s for real-time quality correction. For accurate mass acquisition, leucine enkephalin at a flow rate of 10 μL min^−1^ was used as a lock mass by a lock spray interface to monitor the positive ([M + H]^+^ = 556.2771) and the negative ([M − H]^−^ = 554.2615) ion modes. Data acquisition and analysis were controlled by Waters UNIFI V1.71 software. The scan rang in MS and MS/MS modes were over a range of 50–1,200 m/z.

#### 2.2.3 Analysis of ZaTm extract in relation to brain functions

The dataset of compounds identified through LC–MS/MS analysis of ZaTm extract was rigorously refined to ensure accuracy and consistency. Extraneous characters were removed, retaining only valid entries in the “Component name” column. Each compound was classified using well-established chemical databases: PubChem,^1^ a comprehensive repository of chemical information; ChEBI (Chemical Entities of Biological Interest),^2^ which focuses on biologically relevant molecules; and DrugBank,^3^ a resource dedicated to pharmacologically active substances. Compound names were manually searched within these databases, and their molecular structures, classifications, and properties were systematically reviewed. Compounds that could not be located in any database or did not fit into predefined category were assigned to the “Others” group.

### 2.3 Establishment of a scopolamine-induced mouse model of cognitive and memory deficits

#### 2.3.1 Animals

Eight-week-old male ICR mice (weighing 23–28 g) were purchased from Samtaco (Suwon, Korea). The animals were granted unrestricted access to water and a standard rodent chow diet while housed in an environment maintained at 23 ± 2°C with 53 ± 7% humidity, under a 12-h light/dark cycle. Stringent measures were taken to minimize the use of animals and alleviate any potential suffering. Behavioral tests were evaluated by a blind person throughout the experimental process. All experimental protocols were approved by the Animal Experimentation Ethics Committee of Jeonbuk National University (Approval Number: JBNU2024-0346).

#### 2.3.2 Mouse model of cognitive and memory deficits

To determine the optimal concentration of scopolamine, mice were divided into five groups and administered various doses of scopolamine: (1) control group (*n* = 8), in which mice received 2% DMSO; (2) scopolamine (2 mg/kg) (*n* = 8); (3) scopolamine (3 mg/kg) (*n* = 8); (4) scopolamine (2 mg/kg) + donepezil (5 mg/kg) (*n* = 8); and (5) scopolamine (2 mg/kg) + donepezil (5 mg/kg) (*n* = 8). Donepezil was used as a positive control in cognitive and memory deficit models. Morris water maze (MWM), Y-maze, and light/dark tests were conducted to evaluate changes in cognitive and memory function between the groups in this dose-optimization experiment. H&E, Nissl staining, and western blotting were performed to assess the extent of hippocampal cell damage, as described in the following sections. After identifying the most effective scopolamine dose through behavioral tests, such as spatial learning, memory impairment, and anxiety (Supplementary Figures S1, S2), and evaluating PSD95 and NeuN expression in the hippocampus (Supplementary Figure S3) along with histopathological damage (Supplementary Figure S4), a concentration survey revealed that both 2 and 3 mg/kg of scopolamine induced cognitive and memory deficits in mice. However, when combined with donepezil (5 mg/kg), 2 mg/kg of scopolamine demonstrated better recovery than that with 3 mg/kg of scopolamine. Although donepezil at 5 mg/kg improved cognitive and memory deficits, it did not fully restore the severe nerve cell damage induced by 3 mg/kg scopolamine. Therefore, 2 mg/kg scopolamine was chosen as the optimal dose for recovery, either in combination with 5 mg/kg donepezil or the ZaTm mixture. Following this, mice were divided into nine groups (*n* = 72) as follows: (1) control group (*n* = 8); (2) scopolamine (2 mg/kg) (*n* = 8); (3) scopolamine (2 mg/kg) + donepezil (5 mg/kg) (*n* = 8); (4) scopolamine (2 mg/kg) + ZaTm (100 mg/kg) (*n* = 8); (5) scopolamine (2 mg/kg) + ZaTm (250 mg/kg) (*n* = 8); (6) scopolamine (2 mg/kg) + ZaTm (500 mg/kg) (*n* = 8); (7) scopolamine (2 mg/kg) + ZaTm (1,000 mg/kg) (*n* = 8); (8) ZaTm (100 mg/kg) (*n* = 8); and (9) ZaTm (1,000 mg/kg) (*n* = 8). In the cognitive and memory deficit groups, mice received daily intraperitoneal injections of scopolamine (2 mg/kg) for 8 days, from D24 to D31, with the exception of the control and ZaTm alone groups. Specifically, groups 3–9 were orally administered 0.1 mL of the ZaTm mixture once daily for 24 days, from D8 to D31. The ZaTm extract was administered to the experimental group, while an equivalent volume of water was provided to the control group to ensure consistency. Drug solutions were freshly prepared immediately before administration.

### 2.4 Behavioral tests

#### 2.4.1 Morris water maze test

Morris water maze (MWM) test is a widely used behavioral tool for assessing spatial learning and memory in mice (Morris, 1984). The experimental setup consisted of a water tank with a diameter of 200 cm and depth of 60 cm, filled with water maintained at 24°C ± 2°C to a depth of 42 cm. The tank was divided into four quadrants, with a central platform (10 cm in diameter and 41 cm in height) placed in the third quadrant. Each mouse underwent training and testing sessions, consisting of four trials per day over five consecutive days. In each trial, the mouse was gently released into the water, initially facing the pool wall, from one of the four equidistant starting points around the perimeter of the pool. The mice were given a maximum of 60 s to locate the platform. Upon reaching the platform, the mouse remained there for 15 s. If the platform was not located within 60 s, the trial was terminated and a time of 60 s was recorded. The mice were guided to the platform and allowed to remain there for 15 s. The time required to reach the platform was recorded as a measure of spatial learning. After 5 days of training, an exploratory test was conducted to evaluate spatial memory. During this test, the platform was removed from the water tank, and each mouse was allowed to swim freely for 60 s before being removed from the water. Behavioral data were recorded using a high-definition 64 MP camera (Snapdragon 730-8 cores; Vivo, Dongguan, China) and supported by ANY-maze video tracking software.

#### 2.4.2 Y-maze test

The Y-maze test was conducted to assess learning and memory impairment in mice from D25 to D28, following previously established protocols with minor modifications (Kraeuter et al., 2019). The maze was 60 cm in length, 10 cm in width, and 25 cm height. To increase food motivation, each mouse was subjected to a 10-h fasting period prior to testing. On D25, cheese was strategically placed at the ends of both the left and right arms of the Y-maze, and each mouse was placed in the starting arm. During each session, the mice were allowed a 5-min period of free exploration of the maze. On D26, the directed exploration of both the right and left arms of the maze was introduced, in which each mouse was placed in the starting arm and encouraged to traverse both the left and right arms three times. On D27, the left arm was designated as the correct path, whereas the right arm was identified as incorrect. Each mouse was required to navigate through the left and right arms three times. A correct choice led to access to the cheese reward, whereas an incorrect choice resulted in a 10-s confinement as a penalty. Formal testing occurred on D28, during which each mouse completed five trials in the Y-maze. A camera (HD 64 MP, Snapdragon 730-8 cores) was used to capture the percentage of correct responses. After each trial, the Y-maze apparatus was thoroughly sanitized with 75% ethanol.

#### 2.4.3 Light/dark test

The light/dark test was performed to evaluate the photophobia levels in male mice on D29 and D30 (Xu et al., 2015; Pinton et al., 2011). The enclosed chamber (80 × 80 × 30 cm) was divided into two equal compartments (40 × 40 × 30 cm). Mice were allowed a 10-min period to freely explore the light/dark box, and their movements, as well as the time spent in each compartment, were recorded to assess photophobia-related behaviors across different groups. The data were captured using an HD 64 MP camera (Snapdragon 730-8 cores) and supported by ANY-maze video tracking software.

### 2.5 Western blotting

Brain tissue was homogenized by RIPA buffer containing 150 mM sodium chloride, 1% Triton X-100, 0.5% sodium deoxycholate, 0.1% sodium dodecyl sulfate, and 50 mM Tris (pH 8.0). Protease inhibitor cocktail (Sigma-Aldrich, St. Louis, MO, USA) was added in RIPA buffer right before tissue lysis. The protein concentrations were quantified using the bicinchoninic acid method (Thermo Fisher Scientific). Equal amounts of proteins were separated by sodium dodecyl sulfate-polyacrylamide gel electrophoresis and transferred onto polyvinylidene difluoride membranes. Membranes were blocked with Tris-buffered saline containing 0.1% Tween 20 and 5% skim milk for 1 h at room temperature.

The membranes were incubated overnight with specific primary antibodies (diluted 1:1000) in phosphate-buffered saline with Tween-20 (PBS-T), supplemented with 1% BSA at 4°C. The following day, membranes were incubated with horseradish peroxidase-conjugated secondary antibodies (diluted 1:10,000) in PBS-T containing 1% BSA for 1 h at room temperature. Immunoreactive proteins were visualized using an enhanced chemiluminescence kit (Thermo Scientific™ West Femto maximum sensitivity substrate, #34095) and iBright CL1000 Imaging System (Thermo Fisher Scientific). Protein expression levels were quantified by densitometric analysis using ImageJ software, with intensity values normalized to either the corresponding *α*-tubulin bands or total protein bands.

### 2.6 Histopathological analysis

Hippocampal specimens, specifically from the CA1, CA3, and dentate gyrus (DG) regions, were used. Tissue samples were fixed in 10% buffered formalin and embedded in paraffin blocks. Coronal brain sections, 20 μm in thickness, were prepared using a cryostat (CM 1510S; Leica Microsystems, Germany) and mounted on slides. Sections were deparaffinized using xylene and rehydrated using a graded ethanol series (100, 90, 70, and 50%). Brain slices (20 μm) were then stained using Nissl and senescence-associated beta-galactosidase (SA-β-gal). All procedures were performed according to the manufacturers’ instructions for commercially available kits. Digital images of the CA1, CA3, and DG regions were captured using an optical microscope (Nikon Eclipse Ts2) equipped with a camera (HK6E3 E3CMOS; Nikon Inc., Tokyo, Japan) with identical imaging settings applied to all samples. The evaluation of histological results was determined by qualitative methods. According to the manufacturer’s kit instructions, beta-galactosidase and Nissl staining is labeled by causes the cells to have a characteristic blue color. The cells of the normal hippocampus will have a dense density creating a line that can be observed by a characteristic blue color. However, with a damaged hippocampus, the distribution is not dense, the line is loose or distorted, observed by a characteristic blue color.

### 2.7 Oxidative stress measurement

#### 2.7.1 Estimation of glutathione activity

The levels of reduced glutathione (GSH) in the brain at D31 were determined by measuring absorbance at 412 nm using an Epoch™ Microplate Spectrophotometer (Bio-Tek Instruments). The procedure was performed according to the manufacturer’s guidelines as previously described (Wang et al., 2020).

#### 2.7.2 Estimation of antioxidant enzyme activity

To evaluate the antioxidant enzyme activity, we followed the manufacturer’s protocols for commercially available kits (Wang et al., 2020; Chen et al., 2022). CAT and SOD activities were quantified by measuring the absorbance at 540 and 450 nm, respectively, using an Epoch Microplate Spectrophotometer (Bio-Tek Instruments).

#### 2.7.3 Estimation of MDA activity

MDA levels in the brain at D31 were quantified by measuring absorbance at 532 nm using a microplate reader (Epoch Microplate Spectrophotometer, Bio-Tek Instruments). The procedure was performed according to the manufacturer’s guidelines, as previously described by Ishola et al. (2017).

### 2.8 Data analysis

All statistical analyses were conducted using the GraphPad Prism 7 software (San Diego, CA, USA). Data are presented as mean ± SEM, and comparisons between groups were performed using Student’s *t*-tests to analyze differences. Statistical significance was determined as indicated in the figures (^*^p < 0.05, ^**^p < 0.01, ^***^p < 0.001, compared to the control group; ^#^*p* < 0.05, ^##^*p* < 0.01, ^###^*p* < 0.001, compared to the scopolamine-only group; *ns*, not significant; paired *t*-test).

## 3 Results

### 3.1 The compound components of ZaTm extract may play a protective role against scopolamine-induced cognitive impairments in mice

**Figure 1:**
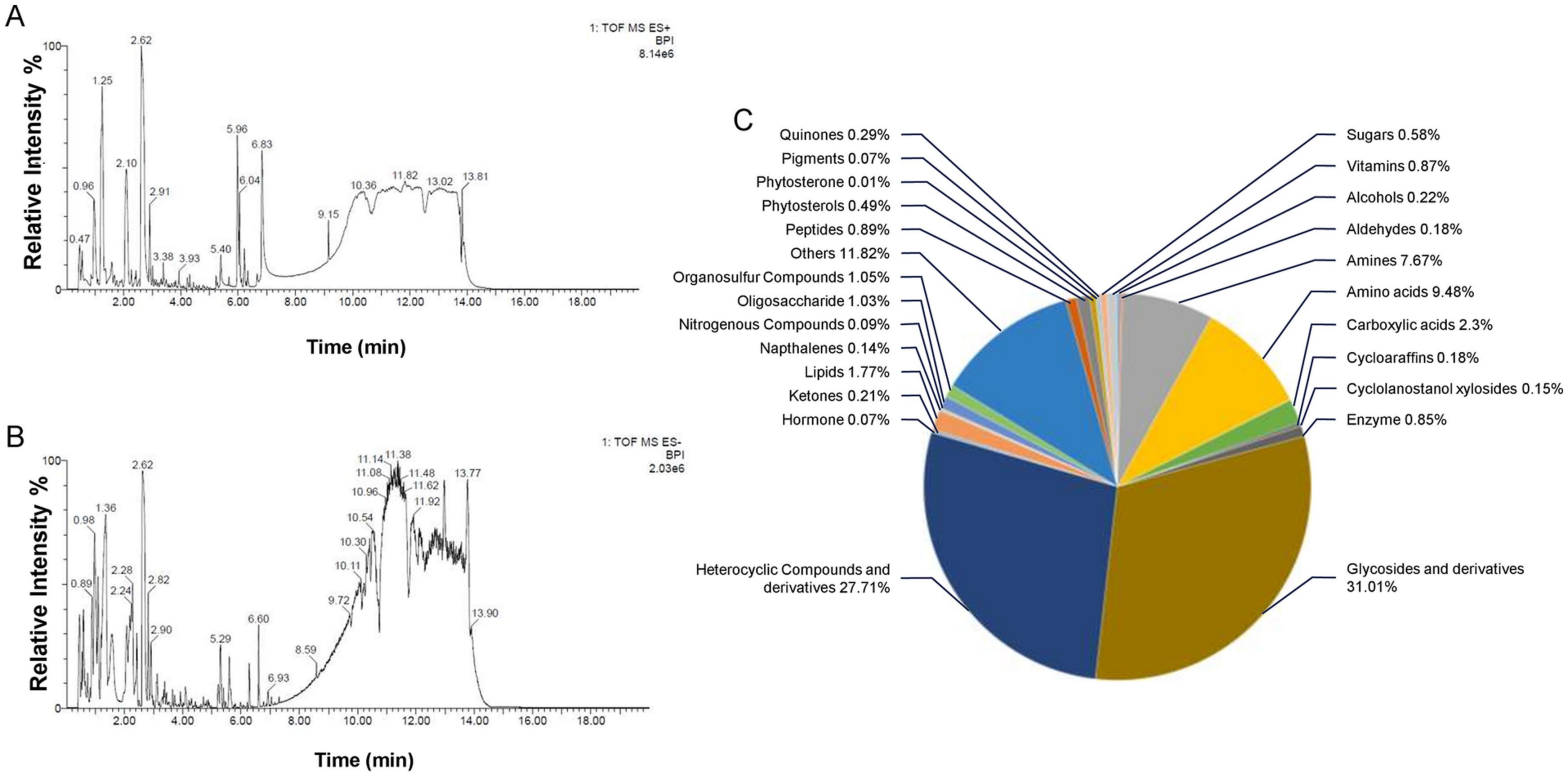
Base peak intensity (BPI) chromatograms of the ZaTm extract analyzed using UPLC-Q-TOF. The chromatograms were obtained in **(A)** negative ion mode and **(B)** positive ion mode. **(C)** A representative graph displaying the categorical distribution of compounds within the ZaTm extract, presented as percentages.

High-resolution MS data of the ZaTm extract was efficiently obtained using the UPLC-Q-TOF method. The base peak intensity (BPI) chromatograms, acquired in both positive and negative ionization modes, are presented in Figure 1. The MS data were analyzed using the UNIFI screening platform, which facilitated automatic fragment matching. Following manual validation, 627 compounds were identified or tentatively characterized in the ZaTm extract. Comprehensive MS details of the identified compounds are provided in Supplementary Table S2.

To evaluate the nutrient composition of the ZaTm extract, individual compounds were meticulously analyzed using established chemical and biological databases, including PubChem, ChEBI, and DrugBank, which offer comprehensive data on biologically relevant compounds and their potential pharmacokinetic properties. The analysis, as summarized in Figure 1C, indicates that glycosides and their derivatives constitute the largest properties (31.01%) of identified compounds. This is followed by heterocyclic compounds and their derivatives (27.71%). Interestingly, 11.82% of the compounds could not be classified into any predefined chemical categories or were absent from the referenced databases. Amino acids accounted for 9.48%, while amines represented 7.67% of the total. The remaining 12.31% were distributed across various other chemical classes, as detailed in Figure 1C.

While glycosides, heterocyclic compounds, amino acids, and amines emerged as the predominant categories, it is important to emphasize that compound outside these classifications may still possess significant bioactive properties relevant to brain health. Tables 1–3 provides an in-depth classification of these compounds, underscoring their potential neuroprotective effects. These include their roles as nutraceuticals, metabolites, or other bioactive agents, which collectively highlight the diverse chemical landscape and therapeutic potential of the ZaTm extract.

**Table 1:** Nutraceuticals^1^ in ZaTm extract and their relevance to brain health.

Class^2^	Sub-class^2^	Component name	Properties
Amino acids		Acetyl-L-tryptophan	Modified amino acid; precursor to serotonin
		DL-Arginine	Precursor to nitric oxide; supports brain function
		DL-Tryptophan	Precursor to serotonin; linked to neurodegenerative pathways
		DL-Tyrosine	Precursor to dopamine; implicated in neurodegenerative diseases
		D-Phenylalanine	Precursor to dopamine; potential role in neuroprotection
		Ethyl glutamate	Precursor to glutamate; supports excitatory neurotransmission
		Glutamic acid	Nutraceutical; a ferroptosis inducer and a neurotransmitter.
		Methionine	Essential amino acid; supports methylation and CNS function
Glycosides	Nucleosides	Cordycepin	Adenosine derivative; supports neuroprotection
Lipids	Fatty acids	Linolic acid	Omega-6 fatty acid; supports brain health and function
Oligosacc-haride		D-(+)-Trehalose	Geroprotector
		Stachyose	Prebiotic with potential neuroprotective effects
Others		Betaine	Cellular hydration and maintaining cell function
Vitamins		Pantothenic acid (Vitamin B5)	Curare poisoning; geroprotector
		Riboflavin (Vitamin B2)	Vitamin B2; supports nervous system metabolism

1 Nutraceuticals are bioactive compounds found in foods or supplements that provide health benefits beyond basic nutrition, often used to prevent or treat diseases, enhance physiological functions, or support overall health.

2 Compounds were classified based on chemical information retrieved from PubChem, ChEBI, and DrugBank databases.

**Table 2:** Metabolites^1^ in ZaTm extract and their relevance to brain health.

Class^2^	Sub-class^2^	Component name	Properties
Amines		(−)-Epinephrine	Neurotransmitter; supports stress response and alertness
		3,4-Dihydroxyphene-thylamine	Dopamine precursor; supports neurotransmitter synthesis
		Tryptamine	Precursor to serotonin; potential role in mood regulation
		Tyramine	Precursor to dopamine; supports neurotransmitter synthesis
Amino acids		5-Oxoproline	Intermediate in glutathione metabolism; supports antioxidant defense
		Histidine	Precursor to histamine; involved in neuroinflammation
		Ornithine	Precursor to urea cycle; supports nitrogen metabolism
		Phenylalanine	Precursor to tyrosine and dopamine synthesis
		Valine	Essential amino acid; supports CNS metabolism
Glycosides	Nucleosides	Adenosine	Neuromodulator with neuroprotective effects
		Adenosine diphos-phate	Involved in energy transfer within cells
		Inosine	Purine nucleoside with neuroprotective effects
		Thymidine	Nucleoside; supports DNA synthesis and repair
		Uridine	Nucleoside; supports neuroplasticity and memory
Heterocyclic compounds	Alkaloids	Xanthine	Purine base; involved in energy metabolism
	Derivatives	5-Hydroxyindole-3-acetic acid	Serotonin metabolite; supports mood regulation
		Adenine	Precursor to nucleotides; supports DNA repair
		Hypoxanthine	Purine derivative; supports energy metabolism
		Indole	Precursor to serotonin; linked to gut-brain axis
Sugars	Sugar Phosphates	D-ribose-5-phosphate	Precursor to nucleotides; supports DNA and RNA synthesis

1 Metabolites are intermediate or end-products of metabolism in living organisms, involved in physiological functions such as energy production, cellular signaling, and biosynthetic pathways.

2 Compounds were classified based on chemical information retrieved from PubChem, ChEBI, and DrugBank databases.

**Table 3:** Other compounds in ZaTm extract and their relevance to brain health.

Class^1^	Sub-Class^1^	Component name	Properties
Glycosides	Derivatives	Bufalin	Na+/K + -ATPase inhibitor; apoptosis inducer; activation AP-1 via MAPK pathway
Heterocyclic compounds	Alkaloids	Chuanxiongzine	Neuroprotective; vasodilator; platelet aggregation inhibitor
		Harmalol	EC 1.4.3.4 (monoamine oxidase) inhibitor
		Liriodenine	EC 3.1.1.7 (acetylcholinesterase) inhibitor, an EC 3.2.1.20 (alpha-glucosidase) inhibitor
		Nuciferine	CNS depressant; glutamic acid antagonist
	Derivatives	Maltol	Depressant properties in mice; potentiates hexobarbital-induced narcosis & inhibits spontaneous motor activity.
		Thiopental	Thiopental binds to the chloride ionophore site of the gamma-aminobutyric acid (GABA)-A/chloride ionophore receptor complex, thereby enhancing the inhibitory actions of GABA-A in the brain. This leads to synaptic inhibition, decreased neuronal excitability and induction of anesthesia. In addition, this agent decreases glutamate (Glu) responses.
	Terpenes	Aconine	NF-kappaB inhibitor
	Xanthenes	Norswertianolin	EC 3.1.1.7 (acetylcholinesterase) inhibitor
Lipids		Canrenone	Increasing sodium excretion and inhibiting potassium excretion.
Others		1-Monomethyl citrate	Monoamine oxidase B inhibitors
		Cimiside C	Inhibit TNF-α-induced VCAM-1 expression; involvement of PPAR-γ upregulation and PI3K, ERK1/2, and PKC signal pathways
Phenols	Benzene derivatives	Magnolol	Neuroprotective components

1 Compounds were classified based on chemical information retrieved from PubChem, ChEBI, and DrugBank databases.

### 3.2 ZaTm extract restores scopolamine-induced body weight loss in a dose-dependent manner

**Figure 2:**
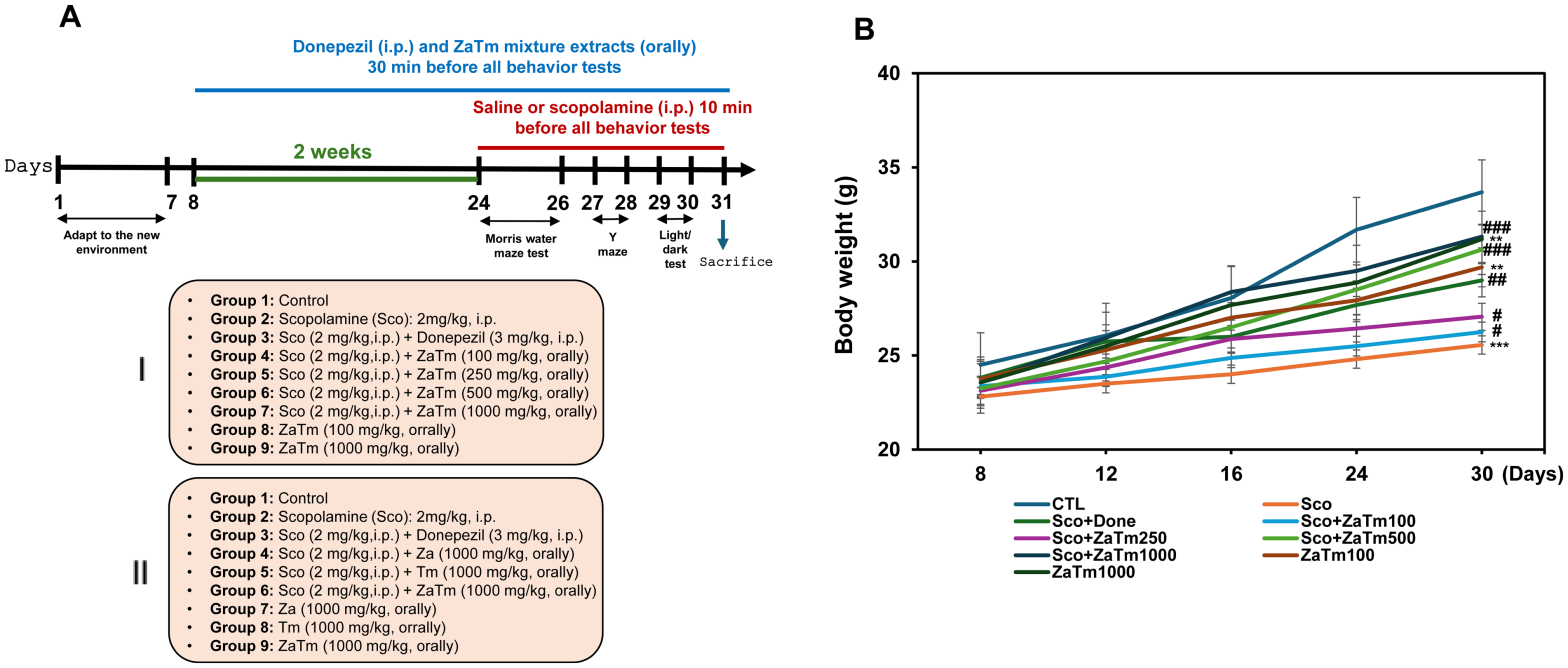
Schematic and body weight of mice for the neuroprotective effects of ZaTm extract in a scopolamine-induced cognitive and memory deficits mouse model. **(A)** Schematic representation of the neuroprotective effects of ZaTm extract on scopolamine-induced cognitive and memory deficits in mice. The experiment spanned a total of 31 days, beginning with a one-day acclimation period for the mice to adjust to their new environment. Following adaptation, donepezil (5 mg/kg) was administered intraperitoneally (i.p.) as a positive control, and ZaTm extract were introduced simultaneously with the donepezil treatment. After 2 weeks of treatment, scopolamine (2 mg/kg, i.p.) was administered for 1 week. During the scopolamine administration period, various behavioral tests were conducted to assess memory and sensory functions. **(B)** Tracking of body weight throughout the experimental period. Additionally, the body weight of the mice was monitored daily throughout the course of the experiment. Although all experimental groups exhibited an increase in body weight, the scopolamine-treated group showed the least amount of weight gain. The control group values were normalized to 1 (mean ± SEM, *n* = 8; **p < 0.01, ***p < 0.001 compared to the control group; ^#^*p* < 0.05, ^##^*p* < 0.01, ^###^*p* < 0.001 compared to the scopolamine-only group; *ns*, not significant; paired *t*-test). ZaTm, *Zophobas atratus* and *Tenebrio molitor* extract mixture.

To induce cognitive and memory impairment in mice, scopolamine was administered with or without the ZaTm extract for 24 days following the adaptation period (Figure 2A). In previous study using either Za or Tm, we found that each edible insect extract provided effective protection against autism spectrum disorder and aging, respectively. To investigate the synergistic efficacy of combining these two edible insects, as well as to compare it with single treatments for cognitive function and neuron protection, we fed mice with Za, Tm, or a ZaTm mixture in this study.

Various behavioral tests, including MWM, Y-maze, and light/dark tests, were conducted 14 days after drug treatment, which was performed 24 days after the commencement of the experiment (Figure 2A). Throughout the treatment period, the body weights of the mice were monitored on alternate days (Figure 2B). The control group demonstrated a natural increase in body weight over the 31-day experimental period, whereas the scopolamine group exhibited a reduced weight gain (Figure 2B). The co-treatment group, consisting of scopolamine and donepezil (a positive control), showed restoration of body weight to levels similar to those of the control group (Figure 2B). However, prolonged administration of scopolamine resulted in a significant reduction in body weight, a marker indicative of aging, compared to that in the control group (Figure 2B). When scopolamine was combined with varying concentrations of ZaTm (100, 250, 500, and 1,000 mg/kg), a significant dose-dependent recovery of body weight was observed (Figure 2B). In contrast, no significant changes in body weight were observed in the group treated with the ZaTm extract alone at any concentration compared with that of the control group (Figure 2B).

### 3.3 ZaTm extract effectively restores spatial learning, memory impairment, and anxiety in scopolamine-induced aging mice

**Figure 3:**
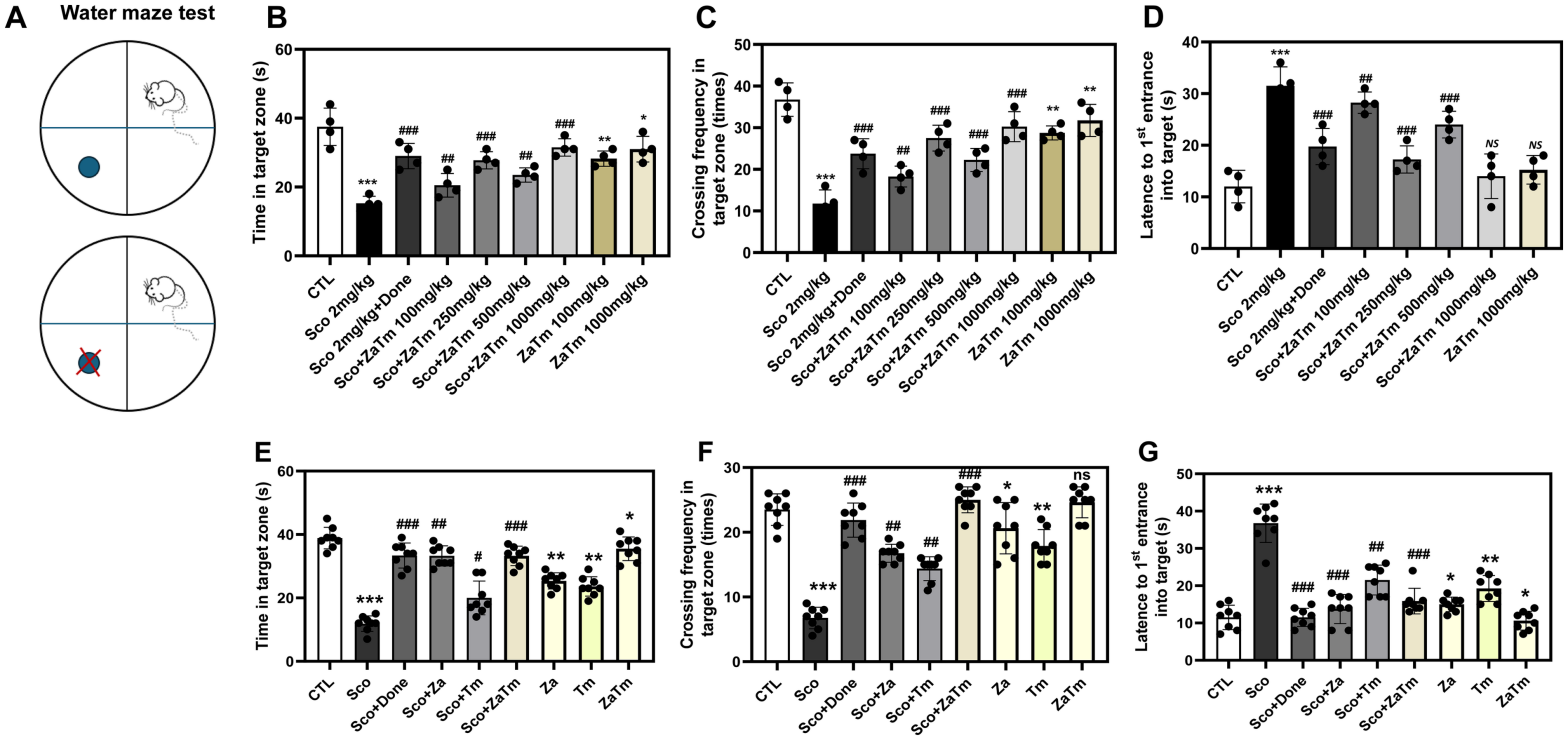
Behavioral assessment using the Morris water maze (MWM) in a scopolamine-induced mouse model of cognitive and memory deficits. **(A)** Schematic overview of the MWM behavioral experiment. This diagram illustrates the experimental design used to assess cognitive and memory functions, focusing on the platform located in one quadrant of the water maze and tracking the movement patterns of the mice across groups. **(B,E)** Time spent on the platform. The duration each group of mice spent on the platform in one specific area of the water maze was recorded and analyzed. **(C,F)** Frequency of platform entry. The frequency with which each group of mice entered the platform or the area surrounding it during the MWM test was measured and compared across groups. **(D,G)** Latency to reach the platform. The time it look for each group of mice to reach the platform or the surrounding area during the MWM test was recorded. The control group’s values were normalized to 1 (mean ± SEM, *n* = 4; *p < 0.05, **p < 0.01, ***p < 0.001 compared to the control group; ^#^*p* < 0.05, ^##^*p* < 0.01, ^###^*p* < 0.001 compared to the scopolamine-only group; *ns*, not significant; paired *t*-test).

**Figure 4:**
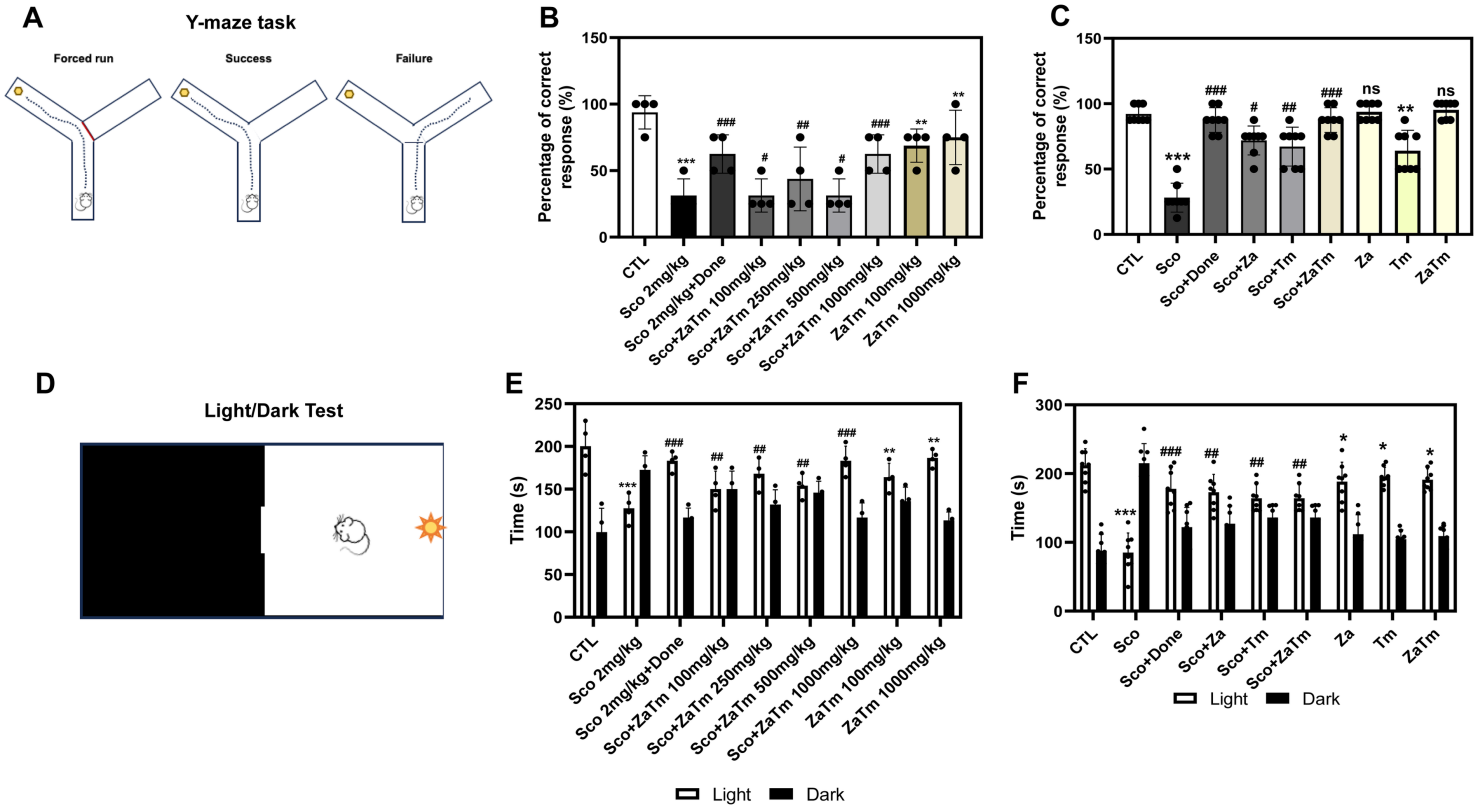
Behavioral testing using the Y-maze and light/dark test in a scopolamine-induced mouse model of cognitive and memory deficits. **(A)** Schematic of the Y-maze behavioral experiment. This diagram illustrates the experimental design for the Y-maze test, where the movements of mice in each group were tracked, focusing on their ability to locate food placed in one arm of the Y-maze. **(B,C)** Percentage of successful food retrieval. The percentage of mice in each group that successfully reached the arm of the Y-maze where the food was located was measured and analyzed. **(D)** Schematic of the light/dark test behavioral experiment. This schematic outlines the design of the light/dark test, tracking the movements of mice in each group as they spent time in either the light or dark compartments of the test apparatus. **(E,F)** Time spent in light/dark conditions. The time spent by each group of mice in the light or dark compartments was measured during the light/dark test. The control group values were normalized to 1 (mean ± SEM, *n* = 4; *p < 0.05, **p < 0.01, ***p < 0.001 compared to the control group; ^#^*p* < 0.05, ^##^*p* < 0.01, ^###^*p* < 0.001 compared to the scopolamine-only group; *ns*, not significant; paired *t*-test).

A decline in learning ability, memory, and cognitive function are the hallmark clinical symptoms associated with aging. The MWM test, which assesses spatial learning in mice, relies on distal cues to guide mice from their starting positions around an open-swimming area to locate a hidden platform. To evaluate the effect of the ZaTm extract on scopolamine-induced cognitive and memory impairment, we used the MWM test (Figure 3A). To determine the concentration most effective at restoring cognitive and memory functions in mice, mixture of two insect extracts were divided into multiple dosage groups and co-administered with scopolamine. Furthermore, to compare the effects of combined treatment with those of single-agent administration (either Za or Tm alone), we evaluated the behavioral changes in animals treated with scopolamine plus Za + Tm at 1000 mg/kg—the dose that proved most effective in combination (Supplementary Figure S5; Figures 3E–G, 4C,F). As depicted in Figures 3B,C, the frequency of crossings and the time spent in the target zone were significantly reduced in the scopolamine-treated group compared to those in the control group. Mice co-treated with scopolamine and donepezil, which served as a positive control, exhibited a moderate recovery in behavioral patterns, demonstrating an increased frequency of crossings and time spent in the target zone, similar to the control group. However, the combined administration of scopolamine and ZaTm significantly improved both the frequency of crossings and the time spent in the target zone compared to that of the scopolamine alone group (Figures 3B,C). Furthermore, the latency period of initial entry into the target zone was extended in the scopolamine group compared to that in the control group (Figure 3D). In contrast, the combination of scopolamine with the ZaTm extract resulted in a shorter latency period for the first entry into the target zone compared with that of the group treated with scopolamine alone (Figure 3D). Notably, in the group treated solely with the ZaTm extract, no significant differences in behavioral patterns were observed compared with that of the control group (Figures 3B–D).

Additionally, the effects of the ZaTm extract on learning and memory impairment were further evaluated using the Y-maze test, which incorporates high-intensity punishment and rewards to encourage mice to select the correct path (Figure 4A). Upon examining the percentage of mice in each group that successfully chose the correct direction, as depicted in Figure 4B, the scopolamine-treated group exhibited a lower percentage of correct responses than that of the control group. In contrast, the group receiving combined treatment with scopolamine and ZaTm demonstrated a significantly higher percentage of correct responses than the scopolamine-only group (Figure 4B). The group treated with ZaTm alone showed no significant behavioral differences compared to those of the control group (Figure 4B). Furthermore, when comparing the effects of the combined treatment (Za plus Tm) with those of single-agent administration (either Za or Tm alone), the combination therapy produced the most robust recovery from scopolamine-mediated learning and memory impairment (Figure 4C). The light/dark test, was conducted to evaluate heightened anxiety levels by measuring the mice’s adaptability to high-intensity lighting conditions (Figure 4D). The anxiety test revealed that the scopolamine-treated group displayed a prolonged preference for the darkened area over the well-lit area compared to the control group (Figure 4E). Conversely, the group subjected to simultaneous treatment with scopolamine and ZaTm displayed a pronounced preference for well-lit areas, with no statistically significant differences observed between the scopolamine and ZaTm-alone groups (Figure 4E). When comparing the effects of single-agent treatment (either Za or Tm) with those of combined therapy (Za + Tm), the combined treatment demonstrated the greatest restoration of anxiety levels (Figure 4F).

### 3.4 ZaTm extract modulates hippocampal neurotrophic factor activities and restores glutamatergic/GABAnergic balance in scopolamine-treated mice

**Figure 5:**
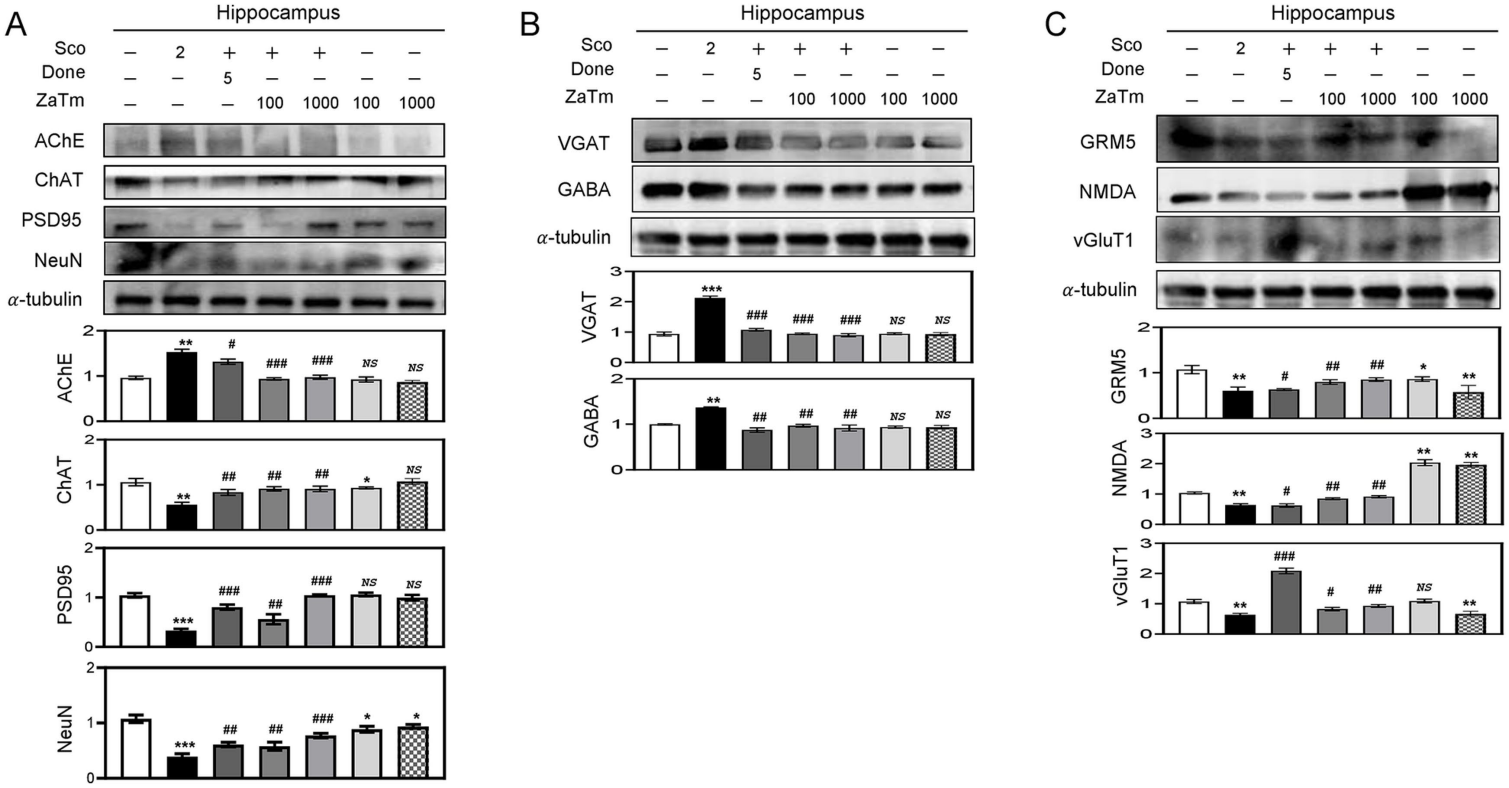
The effect of ZaTm extracts on neurotransmitter regulation and glutamatergic/GABAergic dysfunction in cognitive and memory deficits. **(A–C)** Western blot analysis of the hippocampus. Western blot results from hippocampal samples isolated from each experimental group, probed with antibodies against **(A)** AChE, ChAT, PSD95, NeuN, **(B)** VGAT, GABA, **(C)** GRM5, NMDA, and vGluT1. Equivalent amounts of protein were loaded into each lane, with *α*-tubulin serving as the internal control. The bar graphs represent the fold changes in densitometric values for each protein, normalized to the corresponding α-tubulin bands. The control group’s values were set to 1 (mean ± SEM, *n* = 3; *p < 0.05, **p < 0.01, ***p < 0.001 compared to the control group; ^#^*p* < 0.05, ^##^*p* < 0.01, ^###^*p* < 0.001 compared to the scopolamine-only group; *ns*, not significant; paired *t*-test). ZaTm, *Zophobas atratus* and *Tenebrio molitor* extract mixture.

To assess whether neuronal activity could also be restored by ZaTm treatment, the expression levels of AchE and ChAT, key markers of cholinergic neurons involved in the synthesis or degradation of acetylcholine, were analyzed in the hippocampus using western blotting. For this experiment, we selectively administered 100 and 1,000 mg/kg ZaTm to evaluate the dose-dependent effects. The results revealed that AchE protein expression was significantly elevated in the scopolamine group, whereas ChAT expression was reduced (Figure 5A). The effect of ZaTm extract mixture on scopolamine-induced neuronal damage was assessed using western blot analysis. Among the targets examined, NeuN, a neuronal marker and PSD-95, a marker of neuronal postsynaptic function, were used to measure neuronal damage and senescence. Co-administration of scopolamine and ZaTm restored the scopolamine-induced reduction in NeuN and PSD-95 expression in the hippocampus compared to that in the group treated with scopolamine alone (Figure 5A). Treatment with donepezil moderately restored scopolamine-induced alterations in protein expression (Figure 5A).

In addition to previous findings, we confirmed that ZaTm significantly ameliorates abnormal cognitive function and modulates cholinesterase expression in the hippocampus of scopolamine-treated mice. Therefore, we further assessed the expression levels of key marker proteins involved in maintaining the glutamatergic/GABAergic balance across different experimental groups using western blot analysis. The results demonstrated a significant elevation in the expression of GABA and VGAT proteins in the scopolamine-treated group, accompanied by a reduction in the expression of glutamate metabotropic receptor 5 (GRM5), N-methyl-D-aspartate (NMDA), and vesicular glutamate transporter 1 (vGluT1) (Figures 5B,C). Additionally, co-treatment with scopolamine and the ZaTm extract significantly reduced the expression of VGAT and GABA proteins, while upregulating the expression of GRM5, NMDA, and vGluT1 proteins, compared to the scopolamine only group (Figures 5B,C). In contrast, the ZaTm-only group did not exhibit any significant changes in the expression of these proteins (Figures 5B,C). Donepezil treatment successfully restored the altered expression of AChE, ChAT, PSD95, NeuN, VGAT, GABA, GRM5, NMDA, and vGluT1 to near-normal levels (Figures 5B,C).

### 3.5 ZaTm extract protects the hippocampus from scopolamine-induced oxidative stress in mice

**Figure 6:**
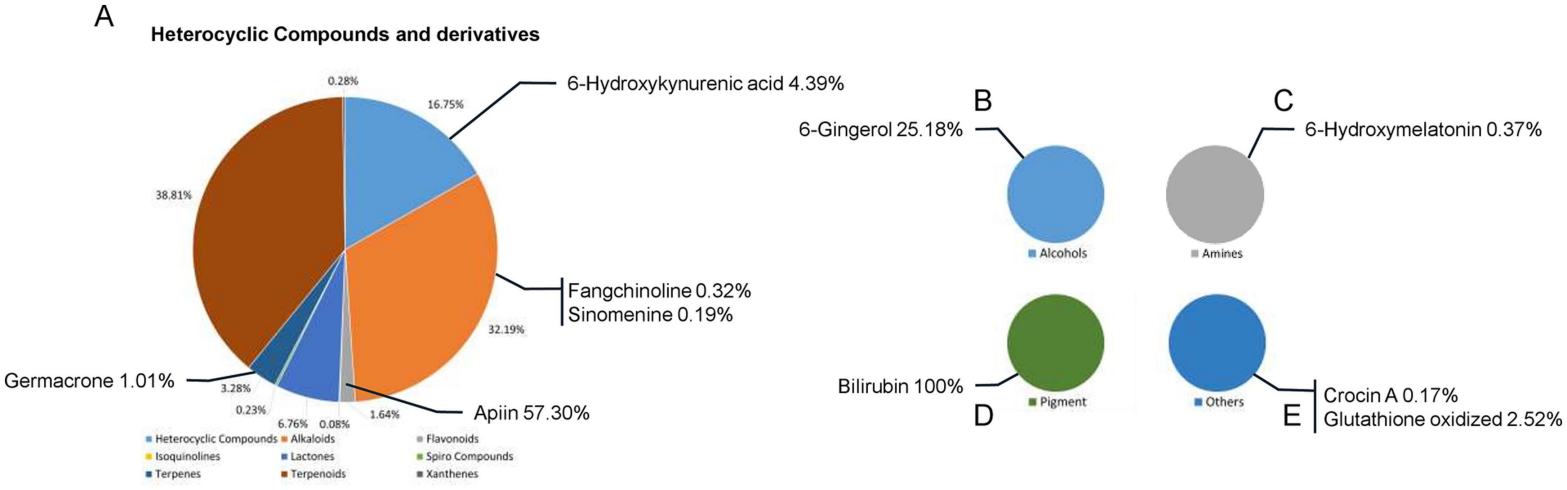
Summary of antioxidant compounds identified in the ZaTm extract, highlighting their potential contributions to brain health. The graphs depict the classification and proportional distribution of antioxidant compounds across various chemical categories: **(A)** heterocyclic compounds and their derivatives, **(B)** alcohols, **(C)** amines, **(D)** pigments, and **(E)** others. The classification and functional annotations of each compound were derived through an in-depth analysis using the PubChem, ChEBI, and DrugBank databases. Percentages represent the relative abundance of each compound within its respective sub-category.

To further elucidate the mechanisms underlying the protective effects of the ZaTm extract, we investigated its antioxidant composition and its potential role in mitigating oxidative stress in scopolamine-induced mice. Figure 6 highlights the key antioxidant compounds present in the ZaTm extract, which may contribute to alleviating oxidative damage in this experimental model. Among the identified antioxidants, Apiin accounted for 57.3% of the flavonoid subclass, while 6-hydroxykynurenic acid constituted 4.39% of the heterocyclic compounds. Additionally, Germacrone, a member of the terpenes class, comprised 1.01% of the mixture (Figure 6A). 6-gingerol, representing the alcohols class, made up 25.18% of the antioxidant profile (Figure 6B). Further analysis identified 6-hydroxymelatonin (Figure 6C) and bilirubin, the sole antioxidant classified as a pigment (Figure 6D). Lastly, oxidized glutathione, a derivative of glutathione, was also present in the antioxidant profile (Figure 6E). Collectively, these compounds suggest that the ZaTm extract possesses significant antioxidant potential, which likely plays a critical role in protecting against scopolamine-induced oxidative stress in mice.

**Figure 7:**
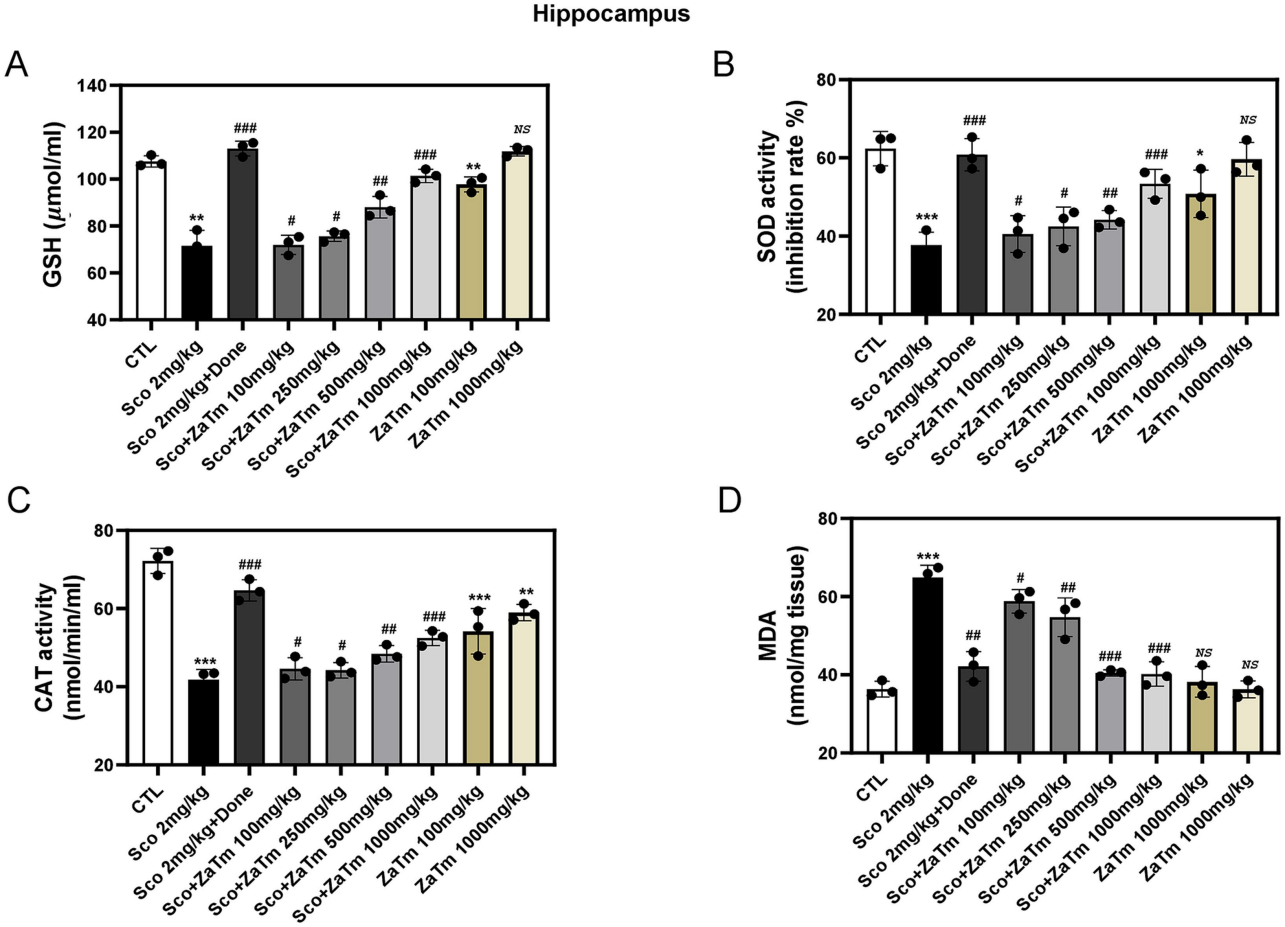
Estimation of glutathione (GSH), superoxide dismutase (SOD), catalase (CAT), and malondialdehyde (MDA) activities in hippocampal tissues. Scopolamine injections resulted in a significant reduction of GSH **(A)**, SOD activity **(B)**, and CAT activity **(C)** in the hippocampus of aged model mice, compared to the control group. Conversely, scopolamine injections caused a marked increase in MDA levels **(D)** in the hippocampus of aged model mice, relative to the control group. The control group’s values were normalized to 1 (mean ± SEM, *n* = 3; *p < 0.05, **p < 0.01, ***p < 0.001 compared to the control group; ^#^*p* < 0.05, ^##^*p* < 0.01, ^###^*p* < 0.001 compared to the scopolamine-only group; *ns*, not significant; paired *t*-test).

Oxidative stress, a major consequence of neuronal toxicity, is a well-established contributor to the development and progression of neurological disorders when sustained over time. To assess the protective effects of the ZaTm extract, we evaluated the activity of key antioxidant enzymes in the hippocampus of scopolamine-treated mice. As illustrated in Figures 7A–C, scopolamine administration significantly reduced the activity of GSH, SOD, and CAT enzymes in the hippocampal lysates compared with those in the control group. Co-administration of the ZaTm extract, however, significantly restored the activities of these enzymes to levels comparable to the control group (Figures 7A–C). Moreover, MDA levels, an indicator of lipid peroxidation, were markedly elevated in the hippocampi of scopolamine-treated mice compared with the control group (Figure 7D). Treatment with the ZaTm extract effectively reduced MDA levels to those observed in the control group (Figure 7D). Importantly, administration of the ZaTm extract alone did not result in significant changes in antioxidant enzyme activities or MDA levels compared with the control group (Figures 7A–D).

### 3.6 The neuroprotective effect of the ZaTm extract in the hippocampus of scopolamine-treated mice is mediated via the AMPK/SIRTs and BDNF-associated Akt/mTOR signaling pathways

Sirtuins (SIRTs) play critical roles in numerous cellular processes, including aging and metabolism. SIRT1 promotes mitochondrial biogenesis and energy production, whereas SIRT3 serves as an activator of proteins involved in oxidative phosphorylation and adenosine monophosphate-activated protein kinase (AMPK). To investigate the involvement of the AMPK/SIRT signaling pathway in the neuroprotective effects of the ZaTm extract during the progression of scopolamine-induced cognitive deficits, we assessed the phospho-AMPK (p-AMPK)/AMPK ratio and SIRT1 and SIRT3 levels in the hippocampus using western blotting.

**Figure 8:**
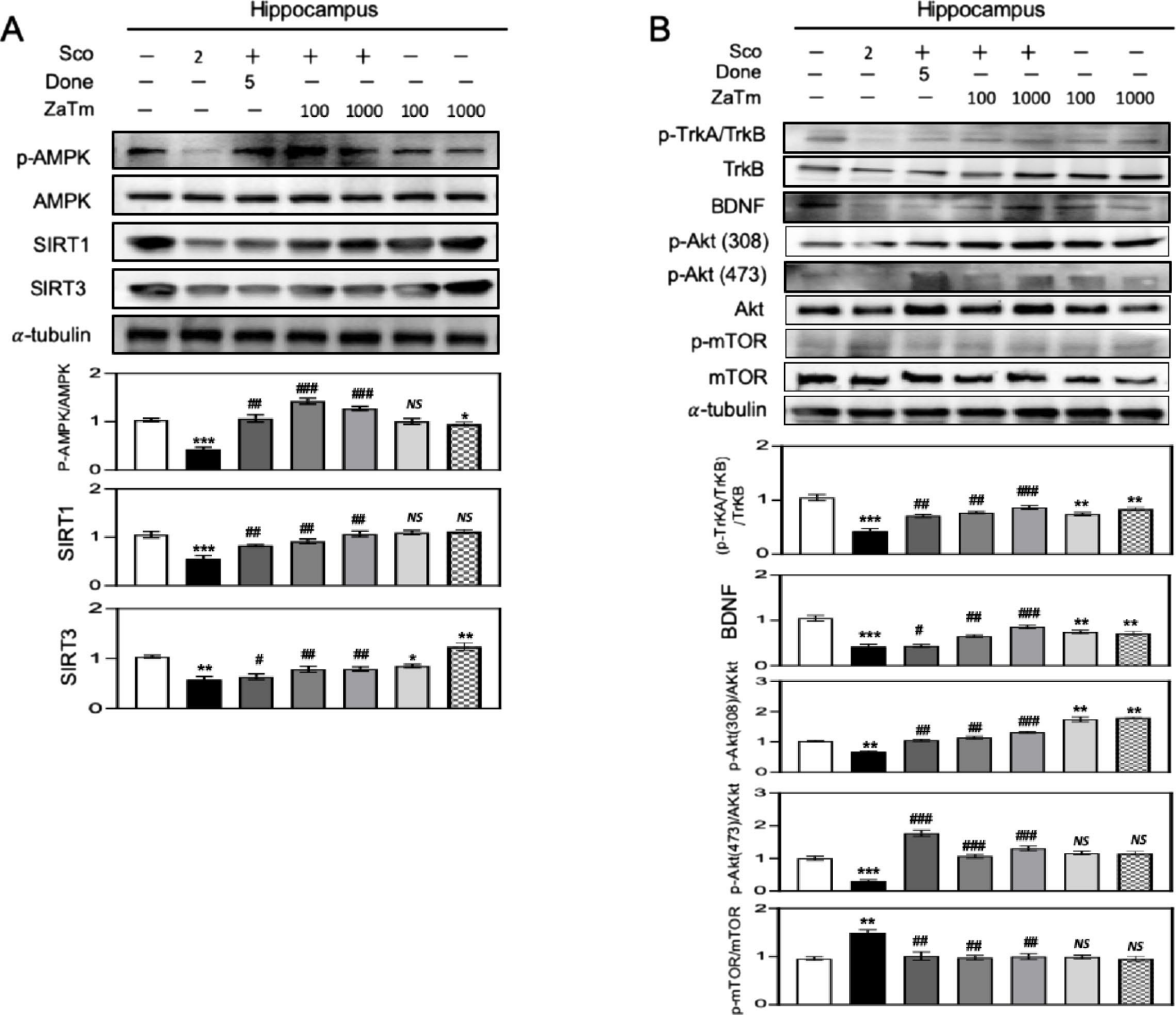
The effect of ZaTm extracts on the AMPK/SIRTs and BDNF-Akt/mTOR signaling pathways in a scopolamine-induced mouse model of cognitive and memory deficits. **(A,B)** Western blot analysis of hippocampal tissues from each group, probed with antibodies against phospho-AMPK (p-AMPK), AMPK, SIRT1, SIRT3, phospho-TrkA/TrkB (p-TrkA/TrkB), TrkB, BDNF, phospho-Akt (p-Akt) (Thr308), phospho-Akt (Ser473), AKT, phospho-mTOR (p-mTOR), and mTOR. Equal amounts of proteins were loaded into each lane, with α-tubulin serving as a control. The bar graphs represent fold changes in the densitometric values of phospho-AMPK (p-AMPK), AMPK, SIRT1, SIRT3, phospho-TrkA/TrkB (p-TrkA/TrkB), TrkB, BDNF, phospho-Akt (p-AKT) (Thr308), phospho-Akt (Ser473), AKT, phospho-mTOR (p-mTOR), and mTOR bands, normalized to the corresponding α-tubulin bands. The control group’s value was set to 1 (mean ± SEM, *n* = 3; *p < 0.05, **p < 0.01, ***p < 0.001 compared to the control group; ^#^*p* < 0.05, ^##^*p* < 0.01, ^###^*p* < 0.001 compared to the scopolamine-only group; *ns*, not significant; paired *t*-test). ZaTm, *Zophobas atratus* and *Tenebrio molitor* extract mixture.

The p-AMPK/AMPK ratio and SIRT1 and SIRT3 expression levels were significantly lower in the scopolamine-treated group than those in the control group (Figure 8A). However, in the positive control group treated with both donepezil and scopolamine, there was a marked increase in the expression of p-AMPK, SIRT1, and SIRT3 compared to that in the scopolamine group (Figure 8A). Co-treatment with either 100 or 1,000 mg/kg ZaTm resulted in a notable upregulation of the p-AMPK/AMPK ratio, as well as SIRT1 and SIRT3 protein levels, compared with those in the scopolamine group (Figure 8A). In contrast, treatment with the ZaTm extract alone, at either 100 or 1,000 mg/kg, did not yield significant changes in protein expression levels relative to those in the control group (Figure 8A).

Furthermore, we explored whether BDNF-Akt/mTOR signaling was implicated in the neuroprotective effects of the ZaTm extract in scopolamine-induced pathogenesis in mice. Specifically, we measured p-Akt expression at two phosphorylation sites, Thr308 and Ser473, as well as BDNF and p-mTOR expression in hippocampal lysates using western blotting. The expression levels of BDNF, p-Akt (Thr308), and p-Akt (Ser473) were significantly reduced, whereas the p-mTOR/mTOR ratio was elevated in the scopolamine-treated group compared with that in the control group (Figure 8B). The positive control group, treated with both donepezil and scopolamine, showed restored protein levels to those observed in the control group (Figure 8B). Similarly, co-treatment with scopolamine and either 100 or 1,000 mg/kg ZaTm produced protein expression results comparable to those observed in the donepezil co-treated group (Figure 8B). However, treatment with either 100 or 1,000 mg/kg of the ZaTm extract alone did not result in significant changes compared to those of the control group (Figure 8B).

### 3.7 ZaTm extract restores senescence-associated hippocampal galactosidase expression and histological damage in scopolamine-induced cognitive deficits in mice

**Figure 9:**
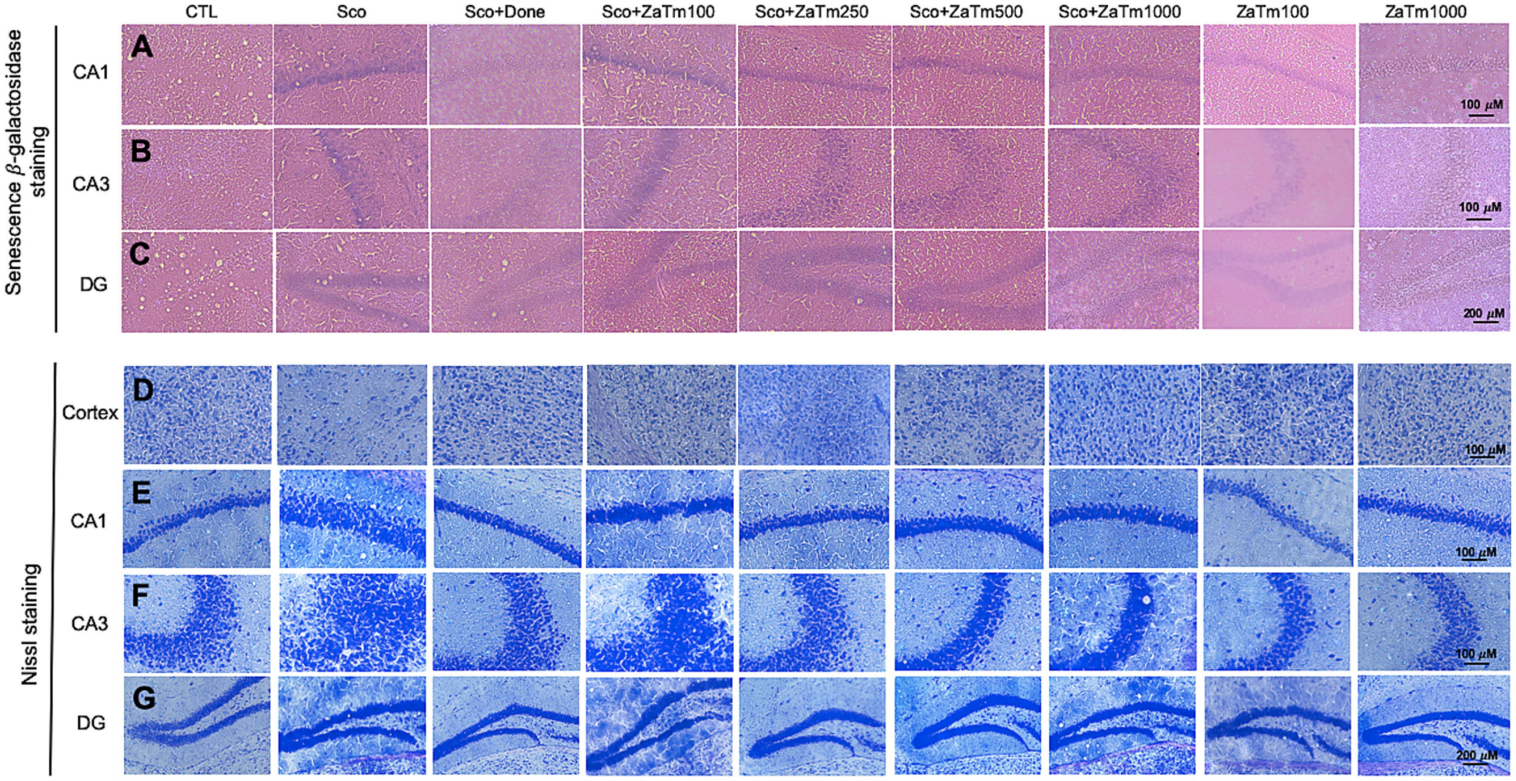
The ZaTm extract’s effects on histopathological damage triggered by senescence and neuronal death in a scopolamine-induced mouse model of cognitive and memory impairments. The cortex and the hippocampal regions, including CA1, CA3, and the dentate gyrus (DG), are critical areas involved in cognitive and memory functions. Representative images of **(A–C)** senescence-associated β-galactosidase staining and **(D–G)** Nissl staining. Staining were performed on sagittal sections of the hippocampus across experimental groups. Scale bars: **(A,B,D,E)** = 100 μm; **(C,F)** = 200 μm. ZaTm, *Zophobas atratus* and *Tenebrio molitor* extract mixture.

To investigate the expression of well-established *in vivo* marker of senescence, SA-β-gal, which exhibits high pH galactosidase activity detectable in senescent cells and tissues. As illustrated in Figures 9A–C, the scopolamine-treated group displayed a markedly elevated rate of SA-β-gal staining compared to that in the control group. Moreover, the group co-treated with scopolamine and donepezil exhibited a significant reduction in the positive expression rate of SA-β-gal staining when compared to that of the scopolamine group (Figures 9A–C). Similarly, the combined treatment with scopolamine and ZaTm extract significantly decreased the percentage of SA-β-gal positive cells relative to that of the scopolamine group (Figures 9A–C). Conversely, treatment with the ZaTm extract alone did not result in a significant difference in SA-β-gal positive cells when compared to that of the control group (Figures 9A–C).

To investigate whether the protective effects of the ZaTm extract against scopolamine-induced cognitive and memory impairments are linked to neuronal damage, we conducted Nissl staining to examine histological changes in the cortex and CA1, CA3, DG regions of the hippocampus (Figures 9D–G). Scopolamine treatment significantly impaired the cortex and CA1, CA3 regions of the hippocampus in mice (Figures 9D–G). However, co-treatment with scopolamine and the ZaTm extract led to a near-complete recovery from scopolamine-induced tissue damage, particularly in the cortex and CA1, CA3 regions of the hippocampus (Figures 9D–G). In contrast, treatment with the ZaTm extract alone did not result in any significant differences when compared to those of the control group (Figures 9D–G).

## 4 Discussion

Neurodegenerative diseases, including AD and PD, along with their associated cognitive and behavioral impairments, currently lack curative treatments, with most therapies focusing on slowing disease progression and alleviating symptoms. In scopolamine-induced models, neurotoxicity leads to cognitive and behavioral deficits in mice. This study explored the neuroprotective potential of a ZaTm extract by examining its capacity to enhance behavioral, cognitive, and memory functions, while countering oxidative stress-induced neurological dysfunction. This dysfunction, marked by the accumulation of neurotoxic substances, neuronal damage, and disruption of key signaling pathways, including AMPK/SIRTs and BDNF-associated Akt/mTOR, plays a pivotal role in neuronal aging.

Abnormal behaviors, including cognitive and memory decline, are hallmark indicators of human neurodegenerative diseases, such as AD, aging-related cognitive impairment, and dementia (O’Leary and Brown, 2024; Tran et al., 2024). In rats, scopolamine administration led to a reduction in average body weight, impaired cognitive performance, and memory deficits, while concurrently increasing anxiety in response to external stimuli, such as social interactions, communication, and exposure to intense lighting (Hamilton et al., 2017). As neurodegenerative processes advance, these symptoms become progressively exacerbated. Treatment with the ZaTm extract markedly ameliorated the behavioral deficits and abnormalities observed in this study. Previous studies highlighted the neuroprotective properties of various plant and insect extracts against a spectrum of neurodegenerative diseases and dysfunctions, including AD, PD, aging, autism spectrum disorders, and epilepsy (Olubodun-Obadun et al., 2024; Salem et al., 2023; Nonato et al., 2024). These findings highlight the potential of natural and medicinal compounds to promote neuroprotection, slow disease progression, and address a wide array of neurological disorders.

Oxidative stress serves as a critical biomarker for AD and insulin resistance, playing a significant role in the pathophysiology of these conditions (Butterfield et al., 2014). Cognitive and behavioral impairments induced by scopolamine are closely linked to oxidative stress within the brain, particularly in the hippocampus, a region that is highly vulnerable to neurological damage characteristic of AD (Mostafa et al., 2021). In this study, we demonstrated that the ZaTm extract markedly mitigated oxidative stress by modulating key biomarkers, including GSH, SOD, CAT, and MDA, thereby alleviating oxidative damage in the hippocampus. Our analysis suggests that various bioactive compounds within the ZaTm extract may synergistically contribute to its antioxidant effects. Notably, Apiin, a flavonoid subclass compound present in the ZaTm extract, has been shown in both *in vitro* and *in vivo* studies to exert substantial scavenging activity against lipofuscin (LPF) and malondialdehyde (MDA), markers indicative of oxidative damage. Moreover, Apiin enhances total antioxidant capacity (TAOC), indicating its role in strengthening the brain’s antioxidative defense mechanisms (Li et al., 2014). Another component, 6-gingerol, a member of the alcohol subclass, has demonstrated protective effects against oxidative stress by reducing the levels of H_2_O_2_, MDA, myeloperoxidase (MPO), nitric oxide (NO), TNF-*α*, and caspase-3, all of which are key indicators of inflammation and apoptosis. Furthermore, bilirubin, traditionally considered a byproduct of heme catabolism, has gained recognition for its potent antioxidant properties. It neutralizes ROS and exhibits neuroprotective effects, underscoring its potential as a therapeutic agent for oxidative stress-related neurodegenerative conditions (Ziberna et al., 2016; Jayanti et al., 2020). Accumulating evidence underscores the critical role of oxidative stress, defined by an imbalance between pro-oxidants and antioxidants leading to excessive ROS production, in the pathogenesis of neurodegenerative diseases. Oxidative stress not only acts as a precursor to neuropathological alterations but is also intricately associated with hallmark features of AD, including metabolic dysregulation, mitochondrial dysfunction, cell cycle anomalies, DNA damage, and neuronal loss (Oladele et al., 2021). Collectively, these pathological cascades manifest clinically as memory deficits, cognitive decline, apathy, depression, and various behavioral abnormalities, which define the progression and symptomatology of AD.

The central cholinergic system significantly influences memory (Burjanadze et al., 2022). Previous research has shown that rats administered scopolamine exhibited reduced neuronal density and heightened AChE activity in the hippocampus compared to those in the control group (Foudah et al., 2023). In our study, ZaTm recovered cholinergic transmission and synaptic plasticity impaired by scopolamine, suggesting that cholinergic regulation via ZaTm in scopolamine-treated mice is closely linked to improvements in behavioral outcomes, neuronal protection, and mitigation of oxidative stress.

The balance between E/I signals is crucial for maintaining proper neural network function and must be tightly regulated to ensure optimal synaptic activity and prevent excitotoxicity (Bi et al., 2020). Hyperexcitability is often associated with inhibitory neurons, particularly GABAergic interneurons. The inability to properly balance the excitability of glutamatergic neurons leads to heightened inhibition, which increases GABA levels (Alcoreza et al., 2021). The ZaTm extract fully restored the imbalance in VGAT and GABA activities, thereby preventing excessive excitation or inhibition that could disrupt plastic mechanisms, such as long-term potentiation and long-term depression. Furthermore, the reduction in vGluT1, a glutamate transporter expressed in the nerve terminals of interneurons and GABAergic astrocytes, was significantly reversed in the hippocampus of mice treated with ZaTm in combination with scopolamine, further underscoring its role in restoring neural equilibrium.

Scopolamine suppresses AMPK activation by inhibiting autophagy and increasing oxidative stress (Jee et al., 2020). Concurrently, key molecules critical for mitochondrial function and antioxidant defense, such as SIRT1 and SIRT3, are downregulated by scopolamine, impairing mitochondrial biogenesis and increasing the vulnerability to oxidative damage (Liu et al., 2024). Consequently, disruption of the AMPK/SIRT pathway amplifies oxidative stress, leads to mitochondrial dysfunction, and accelerates neuronal aging, ultimately contributing to cognitive decline. Therefore, it can be inferred that ZaTm plays a crucial role in the comprehensive regulation of this pathway, thereby mitigating these adverse effects.

BDNF is a crucial neurotrophic factor that enhances protein synthesis by promoting translation initiation through multiple signaling pathways, including Akt (Takei et al., 2001). The BDNF/TrkB/Akt signaling pathway has also been reported to be activated by memory training exercises aimed at detecting spatial reference and working memory (Mizuno et al., 2003; Lee et al., 2020). Scopolamine disrupts BDNF levels, leading to impaired TrkB receptor signaling and inhibition of synaptic strengthening (Sreelatha et al., 2024). Previous studies have shown that disruption of BDNF–TrkB signaling adversely affects the Akt/mTOR pathway, resulting in impaired synaptic plasticity and reduced neuronal resilience (Lee et al., 2020; Shin et al., 2019). The mTOR signaling pathway plays a pivotal role in regulating protein synthesis and degradation, and together with AMPK, it governs both protein quantity and energy homeostasis (Garza-Lombó et al., 2018). Inhibition of BDNF release by scopolamine suggests a reduction in glutamate release and neuronal depolarization. In our study, scopolamine negatively affected the BDNF/TrkB/Akt signaling pathway; however, treatment with the ZaTm extract nearly fully restored the scopolamine-induced alteration of these signals. Reduced glutamate release may also occur through the activation of GABAergic neurons, which subsequently inhibit glutamate transmission (Ghosal et al., 2018). The presence of GABAergic neurons in the hippocampus likely contributes to scopolamine-induced reduction in BDNF expression (Ghosal et al., 2018). Similarly, interventions that enhance BDNF levels result in the activation of Akt/mTOR signaling, AMPK/SIRT activity, and glutamatergic function, highlighting the critical role of BDNF as a central coordinator in both AMPK/SIRT-mediated oxidative stress responses and Akt/mTOR-driven synaptic plasticity.

Many studies have examined the effects of single insect extracts in neurological research; however, each insect contains different bioactive components. In particular, combined treatment has been shown to exhibit synergistic protective effects against cognitive impairment in mice. Although further studies are necessary to precisely elucidate which specific chemicals contribute to particular brain activities, the ZaTm extract demonstrates significant potential as a source of bioactive compounds with antioxidant properties, along with other nutritional components that may benefit brain health. The application of mixed insect extracts in the field of brain health has not been widely explored. Their high nutritional content, antioxidant capacity, and potential to contain various anti-inflammatory compounds underscore the promise of this research, especially given that neuronutrition and oxidative stress are critical in understanding the etiology of many neurological diseases. Moreover, extracts from various edible insects have been shown to modulate the blood–brain barrier, enhance the expression of tight junction proteins, reduce brain damage, alleviate oxidative stress, and inhibit apoptosis. Identifying a single target compound with a strong effect remains a long-term research challenge that encourages further investigation. Therefore, our initial positive results, which reveal several important potential bioactive components, will pave the way for subsequent studies aimed at elucidating the molecular mechanisms underlying these effects. This research is particularly significant as traditional food resources are increasingly scare worldwide, making the discovery of alternative food sources and the development of new pharmaceuticals as urgent priority.

## 5 Conclusion

In conclusion, although further research is necessary to isolate the active compounds in the ZaTm extract responsible for scopolamine-mediated improvements in cognition and memory, this study demonstrates the neuroprotective potential of ZaTm in mitigating scopolamine-induced cognitive and memory deficits in mice. These findings revealed that ZaTm significantly enhanced memory and cognitive performance, restored the altered expression of choline acetyltransferase and acetylcholine esterase, regulated glutamatergic and GABAergic dysfunction, improved antioxidant activity, including GSH, MDA, CAT, and SOD, and prevented hippocampal senescence by modulating the AMPK/SIRT1-SIRT3 and Akt/mTOR signaling pathways. This study highlights the potential of ZaTm as a promising candidate for the development of novel treatments and functional foods aimed at preventing and treating AD and other neurodegenerative disorders.

## Data Availability

The original contributions presented in the study are included in the article/Supplementary material, further inquiries can be directed to the corresponding author.

## Footnotes

^1^ https://pubchem.ncbi.nlm.nih.gov/

^2^ https://www.ebi.ac.uk/chebi/

^3^ https://go.drugbank.com/

## Supplementary material

SUPPLEMENTARY FIGURE S1 Behavioral testing using the Morris water maze (MWM) for optimization of scopolamine in a mouse model of cognitive and memory deficits. **(A)** Schematic of the MWM behavioral experiment. The diagram illustrates the procedure for assessing cognitive and memory function, tracking the movements of mice as they focus on reaching a platform located in one quadrant of the water maze. **(B)** Time spent on the platform. The duration that each group of mice spent on the platform in a specific area of the water maze was recorded and analyzed. **(C)** Frequency of platform entry. The frequency with which each group of mice entered the platform or the surrounding area during the MWM test was measured and compared. **(D)** Latency to reach the platform. The time it look for each group of mice to reach the platform or the surrounding area during the MWM test was recorded. Values for the control group were normalized to 1 (mean ± SEM, *n* = 4; **p* < 0.05, ***p* < 0.01, ****p* < 0.001 compared to the control group; ^#^*p* < 0.05, ^##^*p* < 0.01, ^###^*p* < 0.001 compared to the scopolamine-only group; *ns*, not significant; paired *t*-test).

SUPPLEMENTARY FIGURE S2 Behavioral testing using the Y-maze and light/dark test for optimization of scopolamine in a mouse model of cognitive and memory deficits. **(A)** Schematic of the Y-maze behavioral experiment. This diagram illustrates the procedure of the Y-maze test, tracking the behavior of mice in each group as they navigate toward a food reward located in one arm of the maze. **(B)** Percentage of successful food retrieval. The percentage of mice in each group that successfully reached the arm of the Y-maze containing the food was measured during the test. **(C)** Schematic of the light/dark test behavioral experiment. This schematic outlines the light/dark test, tracking the amount of time each group of mice spent in the light or dark compartments of the test apparatus. **(D)** Time spent in light/dark compartments. The duration that each group of mice spent in the light or dark compartments during the light/dark test was recorded. Values for the control group were normalized to (mean ± SEM, *n* = 4; **p* < 0.05, ***p* < 0.01, ****p* < 0.001 compared to the control group; ^#^*p* < 0.05, ^##^*p* < 0.01, ^###^*p* < 0.001 compared to the scopolamine-only group; *ns*, not significant; paired *t*-test).

SUPPLEMENTARY FIGURE S3 Effect of ZaTm extract on neuronal damage marker expression in the optimization of scopolamine-induced cognitive and memory deficits in mice. **(A)** Western blot analysis of hippocampal samples from each group, probed with antibodies against PSD95 and NeuN. Equal amounts of protein were loaded in each lane, with α-tubulin used as a loading control. The bar graphs represent fold changes in the densitometric values of PSD95 and NeuN bands, normalized to the corresponding α-tubulin bands. **(B)** The graph illustrates the expression levels of PSD95 and NeuN proteins. Control group values were set to (mean ± SEM, *n* = 4; **p* < 0.05, ***p* < 0.01, ****p* < 0.001 compared to the control group; ^#^*p* < 0.05, ^##^*p* < 0.01, ^###^*p* < 0.001 compared to the scopolamine-only group; *ns*, not significant; paired *t*-test). ZaTm, *Zophobas atratus* and *Tenebrio molitor* extract mixture.

SUPPLEMENTARY FIGURE S4 Effects of ZaTm extract on histological damage and neuronal death in the optimization of scopolamine-induced cognitive and memory deficits in mice. The hippocampal regions, including CA1, CA3, and the dentate gyrus (DG), are critical areas associated with cognitive and memory functions. **(A–C)** H&E staining and **(D–F)** Nissl staining were performed on sagittal sections of the hippocampus across the experimental groups. Scale bars: **(A,B,D,E)** = 100 μm; **(C,F)** = 200 μm. H&E, hematoxylin and eosin; ZaTm, *Zophobas atratus* and *Tenebrio molitor* extract mixture.

SUPPLEMENTARY FIGURE S5 The effect of Za and Tm extracts alone on neurotransmitter regulation in cognitive and memory deficits. **(A,B)** Western blot analysis of the hippocampus. Western blot results from hippocampal samples isolated from each experimental group, probed with antibodies against AChE and NeuN. Equivalent amounts of protein were loaded into each lane, with α-tubulin serving as the internal control. The bar graphs represent the fold changes in densitometric values for each protein, normalized to the corresponding α-tubulin bands. (mean ± SEM, *n* = 8; ***p* < 0.01, ****p* < 0.001 compared to the control group; ^#^*p* < 0.05, ^##^*p* < 0.01, ^###^*p* < 0.001 compared to the scopolamine-only group; *ns*, not significant; paired *t*-test). Za, *Zophobas atratus* and Tm, *Tenebrio molitor* alone extract.
