# Supplementary figures and images for: The synergistic extract of Zophobas atratus and Tenebrio molitor regulates neuroplasticity and oxidative stress in a scopolamine-induced cognitive impairment model
Source: Front Aging Neurosci. 2025 Apr 23;17:1566621. doi: 10.3389/fnagi.2025.1566621 (PMC12055851; doi:10.3389/fnagi.2025.1566621)

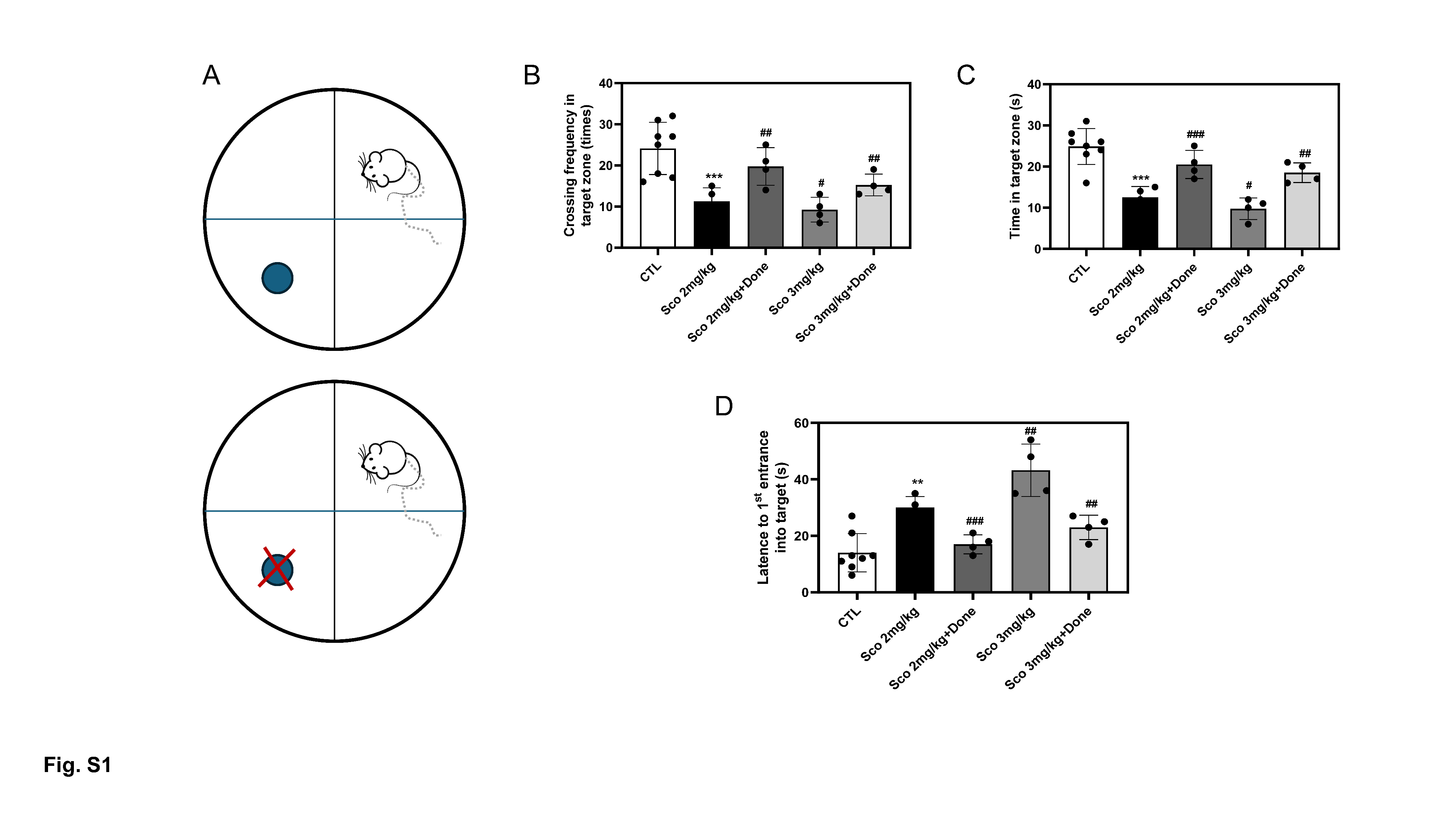

Supplement: SUPPLEMENTARY FIGURE S1 — Behavioral testing using the Morris water maze (MWM) for optimization of scopolamine in a mouse model of cognitive and memory deficits. (A) Schematic of the MWM behavioral experiment. The diagram illustrates the procedure for assessing cognitive and memory function, tracking the movements of mice as they focus on reaching a platform located in one quadrant of the water maze. (B) Time spent on the platform. The duration that each group of mice spent on the platform in a specific area of the water maze was recorded and analyzed. (C) Frequency of platform entry. The frequency with which each group of mice entered the platform or the surrounding area during the MWM test was measured and compared. (D) Latency to reach the platform. The time it look for each group of mice to reach the platform or the surrounding area during the MWM test was recorded. Values for the control group were normalized to 1 (mean ± SEM, n = 4; *p < 0.05, **p < 0.01, ***p < 0.001 compared to the control group; #p < 0.05, ##p < 0.01, ###p < 0.001 compared to the scopolamine-only group; ns, not significant; paired t-test). [file Image_1.tiff]

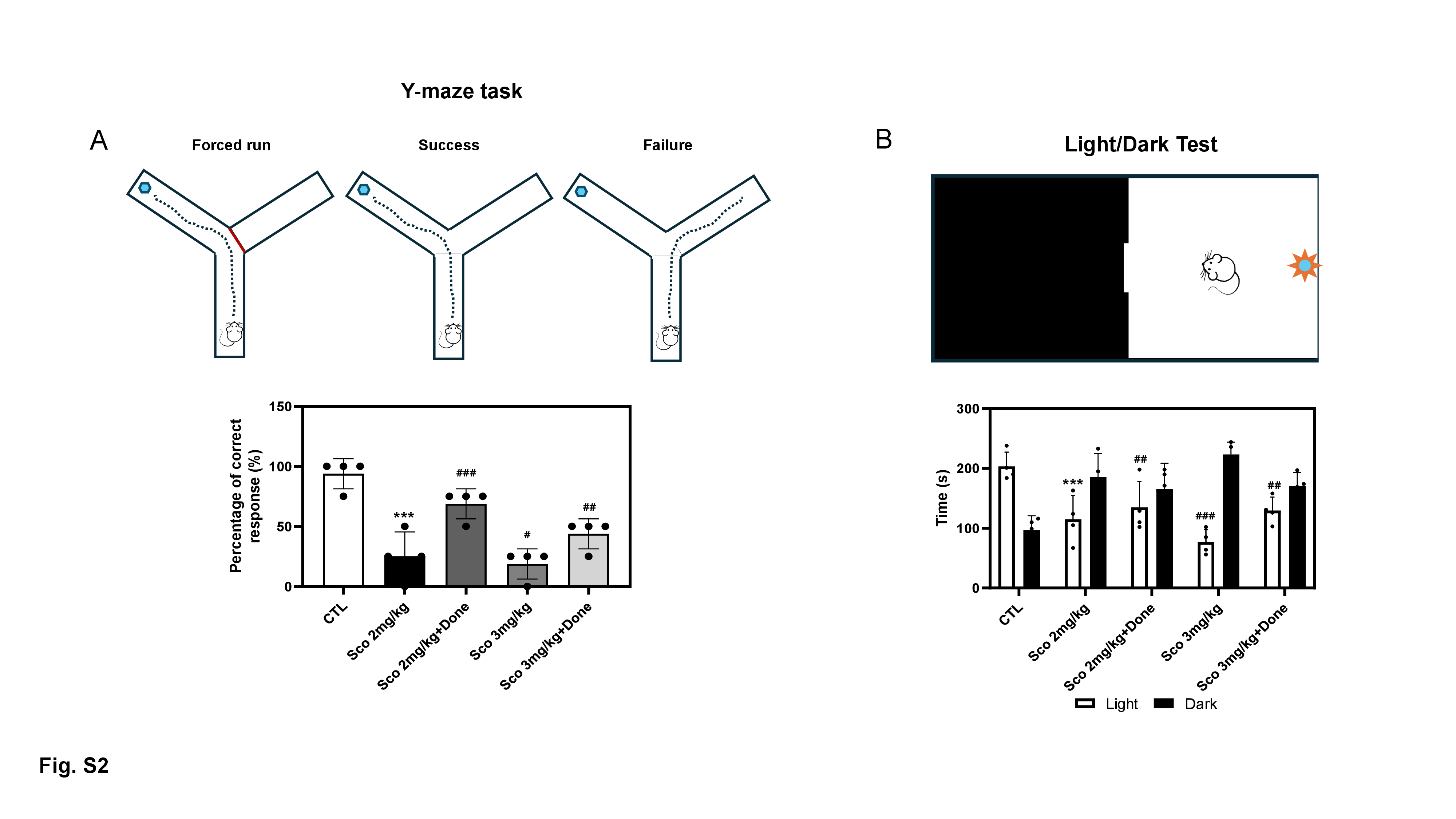

Supplement: SUPPLEMENTARY FIGURE S2 — Behavioral testing using the Y-maze and light/dark test for optimization of scopolamine in a mouse model of cognitive and memory deficits. (A) Schematic of the Y-maze behavioral experiment. This diagram illustrates the procedure of the Y-maze test, tracking the behavior of mice in each group as they navigate toward a food reward located in one arm of the maze. (B) Percentage of successful food retrieval. The percentage of mice in each group that successfully reached the arm of the Y-maze containing the food was measured during the test. (C) Schematic of the light/dark test behavioral experiment. This schematic outlines the light/dark test, tracking the amount of time each group of mice spent in the light or dark compartments of the test apparatus. (D) Time spent in light/dark compartments. The duration that each group of mice spent in the light or dark compartments during the light/dark test was recorded. Values for the control group were normalized to (mean ± SEM, n = 4; *p < 0.05, **p < 0.01, ***p < 0.001 compared to the control group; #p < 0.05, ##p < 0.01, ###p < 0.001 compared to the scopolamine-only group; ns, not significant; paired t-test). [file Image_2.tiff]

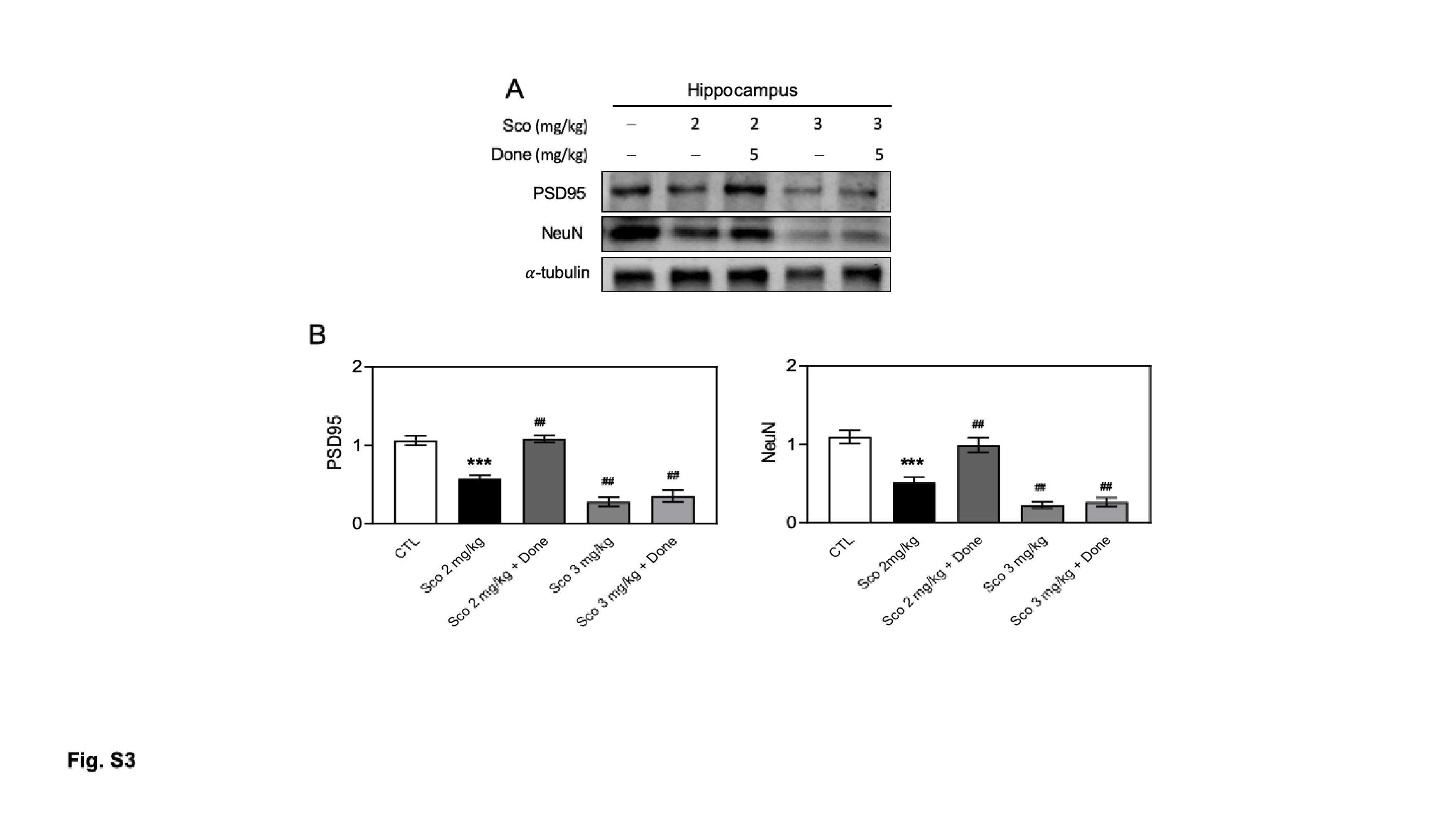

Supplement: SUPPLEMENTARY FIGURE S3 — Effect of ZaTm extract on neuronal damage marker expression in the optimization of scopolamine-induced cognitive and memory deficits in mice. (A) Western blot analysis of hippocampal samples from each group, probed with antibodies against PSD95 and NeuN. Equal amounts of protein were loaded in each lane, with α-tubulin used as a loading control. The bar graphs represent fold changes in the densitometric values of PSD95 and NeuN bands, normalized to the corresponding α-tubulin bands. (B) The graph illustrates the expression levels of PSD95 and NeuN proteins. Control group values were set to (mean ± SEM, n = 4; *p < 0.05, **p < 0.01, ***p < 0.001 compared to the control group; #p < 0.05, ##p < 0.01, ###p < 0.001 compared to the scopolamine-only group; ns, not significant; paired t-test). ZaTm, Zophobas atratus and Tenebrio molitor extract mixture. [file Image_3.tiff]

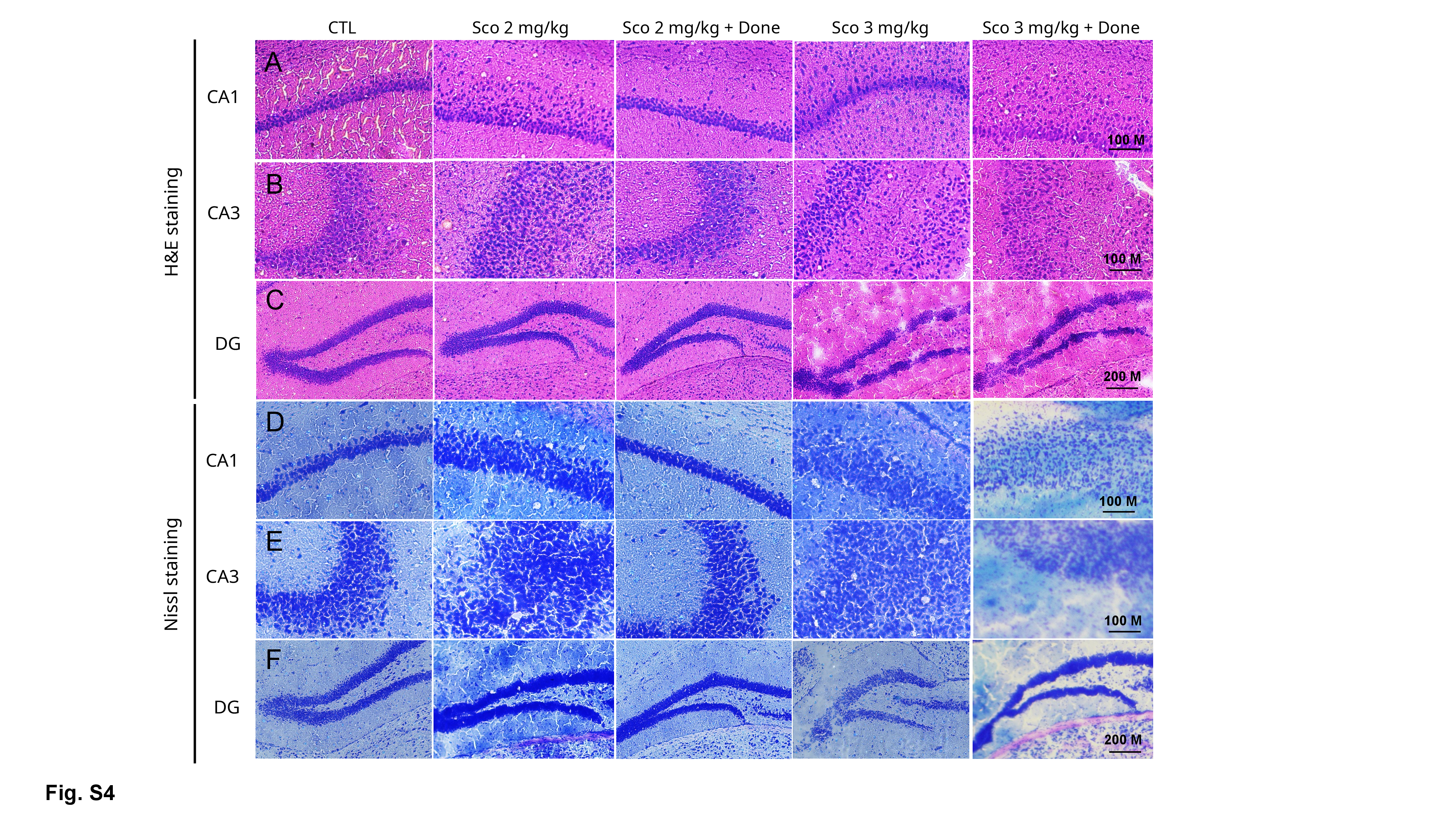

Supplement: SUPPLEMENTARY FIGURE S4 — Effects of ZaTm extract on histological damage and neuronal death in the optimization of scopolamine-induced cognitive and memory deficits in mice. The hippocampal regions, including CA1, CA3, and the dentate gyrus (DG), are critical areas associated with cognitive and memory functions. (A–C) H&E staining and (D–F) Nissl staining were performed on sagittal sections of the hippocampus across the experimental groups. Scale bars: (A,B,D,E) = 100 μm; (C,F) = 200 μm. H&E, hematoxylin and eosin; ZaTm, Zophobas atratus and Tenebrio molitor extract mixture. [file Image_4.tiff]
