# Supplementary material for: The synergistic extract of Zophobas atratus and Tenebrio molitor regulates neuroplasticity and oxidative stress in a scopolamine-induced cognitive impairment model
Source: Front Aging Neurosci. 2025 Apr 23;17:1566621. doi: 10.3389/fnagi.2025.1566621 (PMC12055851; doi:10.3389/fnagi.2025.1566621)
Supplement: Supplementary file 6 [file Data_Sheet_1.docx]

**Supplementary Table 1**. **List of chemicals and antibodies used in this study.**

| **Chemicals** | **Catalog** | **Company** |
| --- | --- | --- |
| Catalase (CAT) activity colorimetric/fluorometric assay kit | K773-100 | BioVision Inc. (Waltham, MA, USA) |
| Donepezil hydrochloride | D6821-10MG | Sigma-Aldrich (St. Louis, MO, USA) |
| EnzyChrom^TM^ GSH/GSSG Assay Kit | EGTT-100 | BioAssay Systems (Hayward, CA, USA) |
| Hematoxylin and eosin (H&E) staining kit | ab245880 | Abcam (Cambridge, UK) |
| Lipid peroxidation (malondialdehyde [MDA]) assay kits | ab118970 | Abcam (Cambridge, UK) |
| Protease inhibitor cocktail | P8340 | Sigma-Aldrich (St. Louis, MO, USA) |
| Scopolamine hydrobromide | S0929 | Sigma-Aldrich (St. Louis, MO, USA) |
| Senescence β-Galactosidase Staining Kit | 9860 | Cell Signaling Technology (Danvers, MA, USA) |
| Superoxide dismutase (SOD) activity assay kit | K335-100 | BioVision Inc. (Waltham, MA, USA) |
| Toluidine blue | 89640 | Sigma-Aldrich (St. Louis, MO, USA) |
| Za and Tm powders (100% pure) |  | Purnae Company (Sejong, Korea) |
|  |  |  |
| **Antibodies** | **Catalog** | **Company** |
| AChE | MBS9605181 | MyBioSource, Inc. (San Diego, CA, USA) |
| Akt (pan) (C67E7) | 4691S | Cell Signaling Technology (Danvers, MA, USA) |
| AMPKα (D5A2) | 5831T | Cell Signaling Technology (Danvers, MA, USA) |
| Brain-derived neurotrophic factor (BDNF) | GTX132621 | GeneTex, Inc. (Irvine, CA, USA) |
| ChAT antibody | ab178850 | Abcam (Cambridge, UK) |
| Gamma-aminobutyric acid (GABA) A receptor alpha 1 | ab252430 | Abcam (Cambridge, UK) |
| GRM5 | CSB-PA003236 | Cusabio Technology (Houston, TX, USA) |
| mTOR | 2972 | Cell Signaling Technology (Danvers, MA, USA) |
| NeuN | ab104224 | Abcam (Cambridge, UK) |
| NMDA R, NR1 subunit antibody | NB300-114 | Novus Biologicals (Centennial, USA) |
| Phospho-Akt (S473) | 4060 | Cell Signaling Technology (Danvers, MA, USA) |
| Phospho-Akt (T308) | 13038 | Cell Signaling Technology (Danvers, MA, USA) |
| Phospho-mTOR (Ser2448) | 2535 | Cell Signaling Technology (Danvers, MA, USA) |
| Phospho-TrkA (Tyr674/675)/TrkB (Tyr706/707) | 4621 | Cell Signaling Technology (Danvers, MA, USA) |
| PSD-95 | ab192757 | Abcam (Cambridge, UK) |
| SIRT1 | GTX134606 | GeneTex, Inc. (Irvine, CA, USA) |
| SIRT3 | 5490 | Cell Signaling Technology (Danvers, MA, USA) |
| TrkB | GTX133722 | GeneTex, Inc. (Irvine, CA, USA) |
| Vesicular GABA transporter | GTX101908 | GeneTex, Inc. (Irvine, CA, USA) |
| vGluT1 | GTX133148 | GeneTex, Inc. (Irvine, CA, USA) |
| α-Tubulin | BS1699 | Bioworld Technology (Nanjing, China) |

**Supplementary Table 2. Identification of insect extract powder by UPLC-Q-TOF**

| **No.** | **Component name** | **t_R_ (min)** | **Neutral mass (Da)** | **Formula** | **Observed neutral mass (Da)** | **Experimental mass m/z** |
| --- | --- | --- | --- | --- | --- | --- |
| 1 | (-)-Acuminatin | 0.47 | 339.99605 | C_6_H_14_O_12_P_2_ | 339.9963 | 338.989 |
| 2 | Arecatannin A2 | 0.5 | 155.06948 | C_6_H_9_N_3_O_2_ | 155.0692 | 156.0765 |
| 3 | (-)-trans-Sobrerol | 0.5 | 260.02972 | C_6_H_13_O_9_P | 260.0302 | 259.0229 |
| 4 | Arnebinone | 0.52 | 155.06948 | C_6_H_9_N_3_O_2_ | 155.0695 | 154.0622 |
| 5 | 2-Formyl-5-(hydroxymethyl) pyrrole | 0.52 | 230.01915 | C_5_H_11_O_8_P | 230.0196 | 229.0123 |
| 6 | 19β-Glucosyl-14-deoxyandrographoside | 0.53 | 174.11168 | C_6_H_14_N_4_O_2_ | 174.1118 | 175.1191 |
| 7 | 1-Methoxy-3,7-dimethyl-2,6-octadiene | 0.53 | 174.11168 | C_6_H_14_N_4_O_2_ | 174.1118 | 173.1045 |
| 8 | Macrostemonoside J | 0.53 | 132.08988 | C_5_H_12_N_2_O_2_ | 132.09 | 131.0827 |
| 9 | Acetophenone | 0.54 | 147.05316 | C_5_H_9_NO_4_ | 147.0533 | 146.046 |
| 10 | Dihydrogentianine | 0.55 | 133.05276 | C_8_H_7_NO | 133.0528 | 156.042 |
| 11 | Corynoxine | 0.56 | 332.0896 | C_17_H_16_O_7_ | 332.0879 | 377.0861 |
| 12 | Isodidehydrotubero-stemonine | 0.56 | 504.16903 | C_18_H_32_O_16_ | 504.1702 | 549.1684 |
| 13 | Ciwujianoside C1 | 0.56 | 408.10565 | C_19_H_20_O_10_ | 408.1044 | 453.1026 |
| 14 | Crocin A | 0.57 | 117.07898 | C_5_H_11_NO_2_ | 117.0785 | 118.0858 |
| 15 | Nootkatone | 0.57 | 656.19525 | C_29_H_36_O_17_ | 656.1944 | 701.1926 |
| 16 | Sanjoinenine | 0.57 | 666.22186 | C_24_H_42_O_21_ | 666.223 | 665.2158 |
| 17 | Theasinensin A | 0.57 | 638.22643 | C_36_H_34_N_2_O_9_ | 638.2281 | 683.2263 |
| 18 | Chuanxiongzine | 0.58 | 342.11621 | C_12_H_22_O_11_ | 342.1162 | 365.1054 |
| 19 | Cynanoside P5 | 0.59 | 115.06333 | C_5_H_9_NO_2_ | 115.0629 | 116.0702 |
| 20 | Tuberostemoenone | 0.59 | 244.06954 | C_9_H_12_N_2_O_6_ | 244.0703 | 243.0631 |
| 21 | Prunose Ⅱ | 0.59 | 210.07395 | C_7_H_14_O_7_ | 210.0744 | 209.0671 |
| 22 | Esculentoside A | 0.6 | 122.02239 | C_4_H_10_S_2_ | 122.023 | 167.0212 |
| 23 | Chlorogenin | 0.6 | 268.08077 | C_10_H_12_N_4_O_5_ | 268.0811 | 267.0738 |
| 24 | Cimidahuside D | 0.6 | 203.1171 | C_10_H_13_N_5_ | 203.1165 | 204.1238 |
| 25 | Aconifine | 0.6 | 612.15196 | C_20_H_32_N_6_O_12_S_2_ | 612.1521 | 611.1448 |
| 26 | Chikusetsusaponin Ib | 0.61 | 268.08077 | C_10_H_12_N_4_O_5_ | 268.0821 | 269.0893 |
| 27 | Acanthoside K2 | 0.61 | 218.16707 | C_15_H_22_O | 218.1661 | 241.1553 |
| 28 | Acankoreoside K | 0.61 | 165.07898 | C_9_H_11_NO_2_ | 165.0792 | 166.0865 |
| 29 | Acetyl-L-tryptophan | 0.62 | 612.15196 | C_20_H_32_N_6_O_12_S_2_ | 612.1524 | 613.1597 |
| 30 | Betaine | 0.62 | 136.03851 | C_5_H_4_N_4_O | 136.0389 | 137.0461 |
| 31 | Aesculioside H | 0.62 | 151.04941 | C_5_H_5_N_5_O | 151.0499 | 152.0571 |
| 32 | Gentioflavine | 0.62 | 189.06372 | C_7_H_11_NO_5_ | 189.0642 | 188.057 |
| 33 | Taurodeoxycholic acid | 0.62 | 914.15417 | C_44_H_34_O_22_ | 914.1534 | 959.1516 |
| 34 | Lycoctonine | 0.63 | 164.04734 | C_9_H_8_O_3_ | 164.0475 | 165.0548 |
| 35 | Oligomycin | 0.63 | 122.03678 | C_7_H_6_O_2_ | 122.0365 | 123.0438 |
| 36 | (+)-Dethiobiotin | 0.63 | 260.02972 | C_6_H_13_O_9_P | 260.0301 | 259.0228 |
| 37 | 2-Furoic acid | 0.64 | 230.01915 | C_5_H_11_O_8_P | 230.0196 | 229.0123 |
| 38 | Histidine | 0.64 | 584.13773 | C_25_H_28_O_16_ | 584.1371 | 583.1298 |
| 39 | Coronaric acid | 0.65 | 332.0896 | C_17_H_16_O_7_ | 332.088 | 377.0862 |
| 40 | N-Methylanthranilic acid | 0.65 | 656.19525 | C_29_H_36_O_17_ | 656.1942 | 701.1924 |
| 41 | Liensinine | 0.65 | 422.08491 | C_19_H_18_O_11_ | 422.0832 | 421.0759 |
| 42 | Lablab saponin I | 0.65 | 131.09463 | C_6_H_13_NO_2_ | 131.0945 | 132.1018 |
| 43 | Ciwujianoside D1 | 0.65 | 408.10565 | C_19_H_20_O_10_ | 408.1043 | 453.1025 |
| 44 | (-)-Epinephrine | 0.66 | 339.99605 | C_6_H_14_O_12_P_2_ | 339.9968 | 338.9895 |
| 45 | Deoxyaconitine | 0.66 | 528.14203 | C_30_H_24_O_9_ | 528.1439 | 527.1366 |
| 46 | Protoveratrine A | 0.66 | 210.07395 | C_7_H_14_O_7_ | 210.0747 | 209.0675 |
| 47 | Arg | 0.68 | 155.06948 | C_6_H_9_N_3_O_2_ | 155.0694 | 156.0767 |
| 48 | Dibutyl sebacate | 0.68 | 342.11621 | C_12_H_22_O_11_ | 342.1174 | 387.1156 |
| 49 | Dioscoreside B | 0.68 | 504.16903 | C_18_H_32_O_16_ | 504.1705 | 503.1632 |
| 50 | Lucidenic acid P | 0.68 | 666.22186 | C_24_H_42_O_21_ | 666.2226 | 665.2153 |
| 51 | 19β-Glucosylandrographoside | 0.69 | 174.11168 | C_6_H_14_N_4_O_2_ | 174.1119 | 175.1191 |
| 52 | D-Fructose 1-phosphate | 0.69 | 126.03169 | C_6_H_6_O_3_ | 126.0318 | 125.0245 |
| 53 | Sarcostin | 0.7 | 342.11621 | C_12_H_22_O_11_ | 342.1162 | 365.1055 |
| 54 | Saponin PB | 0.72 | 118.02661 | C_4_H_6_O_4_ | 118.0267 | 117.0195 |
| 55 | Neomangiferin | 0.75 | 504.16903 | C_18_H_32_O_16_ | 504.1684 | 527.1577 |
| 56 | Cordycepin | 0.77 | 332.0896 | C_17_H_16_O_7_ | 332.0882 | 377.0864 |
| 57 | Isoescin Ⅲ a | 0.78 | 504.16903 | C_18_H_32_O_16_ | 504.1701 | 549.1683 |
| 58 | Prunose Ⅲ | 0.78 | 210.07395 | C_7_H_14_O_7_ | 210.0745 | 209.0672 |
| 59 | Sanjoinine A | 0.85 | 666.22186 | C_24_H_42_O_21_ | 666.2232 | 711.2214 |
| 60 | Nodularin | 0.85 | 656.19525 | C_29_H_36_O_17_ | 656.1945 | 701.1927 |
| 61 | Samarcandin acetate | 0.87 | 666.22186 | C_24_H_42_O_21_ | 666.2226 | 667.2299 |
| 62 | Esculentoside I | 0.89 | 122.02239 | C_4_H_10_S_2_ | 122.023 | 167.0212 |
| 63 | Acanthopanax cerebroside C | 0.89 | 165.07898 | C_9_H_11_NO_2_ | 165.0787 | 166.0859 |
| 64 | Aconine | 0.92 | 612.15196 | C_20_H_32_N_6_O_12_S_2_ | 612.153 | 611.1457 |
| 65 | Cynanoside R3 | 0.92 | 275.05824 | C_17_H_9_NO_3_ | 275.0569 | 274.0496 |
| 66 | Achyranthesterone A | 0.93 | 612.15196 | C_20_H_32_N_6_O_12_S_2_ | 612.1526 | 613.1598 |
| 67 | Wuchuyuamide-Ⅰ | 0.93 | 152.03343 | C_5_H_4_N_4_O_2_ | 152.0338 | 151.0265 |
| 68 | Xanthevodine | 0.93 | 401.11444 | C_17_H_23_NO_8_S | 401.1134 | 424.1027 |
| 69 | Physalin H | 0.94 | 162.06808 | C_10_H_10_O_2_ | 162.0681 | 163.0754 |
| 70 | 2′-Acetylangelicin | 0.98 | 181.07389 | C_9_H_11_NO_3_ | 181.0741 | 180.0668 |
| 71 | 2,3-Dihydroy-farnesol | 0.98 | 181.07389 | C_9_H_11_NO_3_ | 181.0743 | 182.0815 |
| 72 | Lycoposerramine I | 0.98 | 164.04734 | C_9_H_8_O_3_ | 164.0476 | 163.0403 |
| 73 | Tryptamine | 0.98 | 137.08406 | C_8_H_11_NO | 137.0847 | 136.0774 |
| 74 | Lycoposerramine D | 0.99 | 164.04734 | C_9_H_8_O_3_ | 164.0475 | 165.0548 |
| 75 | Ophiopogonin A | 0.99 | 122.03678 | C_7_H_6_O_2_ | 122.0365 | 123.0438 |
| 76 | Tuduranine | 1 | 244.06954 | C_9_H_12_N_2_O_6_ | 244.0701 | 243.0628 |
| 77 | Germacrone | 1.03 | 189.06372 | C_7_H_11_NO_5_ | 189.064 | 188.0567 |
| 78 | Esculentagenin | 1.03 | 122.02239 | C_4_H_10_S_2_ | 122.0231 | 167.0213 |
| 79 | Cnidilide | 1.04 | 332.0896 | C_17_H_16_O_7_ | 332.0888 | 377.087 |
| 80 | Nigellamose | 1.05 | 115.06333 | C_5_H_9_NO_2_ | 115.0635 | 114.0562 |
| 81 | Inosine | 1.05 | 504.16903 | C_18_H_32_O_16_ | 504.1715 | 503.1642 |
| 82 | L-Allylglycine | 1.06 | 131.09463 | C_6_H_13_NO_2_ | 131.0949 | 130.0876 |
| 83 | Anhalinine | 1.1 | 151.04941 | C_5_H_5_N_5_O | 151.0496 | 150.0423 |
| 84 | Alantolactone | 1.11 | 151.04941 | C_5_H_5_N_5_O | 151.0496 | 152.0569 |
| 85 | Saponin PA | 1.11 | 118.02661 | C_4_H_6_O_4_ | 118.0266 | 117.0193 |
| 86 | Gentianal | 1.12 | 240.20893 | C_15_H_28_O_2_ | 240.2079 | 263.1971 |
| 87 | Lactinolide | 1.13 | 131.09463 | C_6_H_13_NO_2_ | 131.0945 | 132.1017 |
| 88 | Escin Ⅳe | 1.15 | 122.02239 | C_4_H_10_S_2_ | 122.0232 | 167.0214 |
| 89 | 2,5-Dimethyl-1,4-benzoquinone | 1.16 | 181.07389 | C_9_H_11_NO_3_ | 181.0741 | 180.0668 |
| 90 | Dictamnol | 1.16 | 342.11621 | C_12_H_22_O_11_ | 342.117 | 341.1097 |
| 91 | Phosphatidyl ethanolamines | 1.2 | 614.16356 | C_30_H_30_O_14_ | 614.1609 | 613.1536 |
| 92 | Laudanosine | 1.21 | 131.09463 | C_6_H_13_NO_2_ | 131.0947 | 130.0874 |
| 93 | Ginsenoside Rs1 | 1.21 | 402.16785 | C_22_H_26_O_7_ | 402.1665 | 447.1648 |
| 94 | Aquilegiolide | 1.21 | 396.14203 | C_19_H_24_O_9_ | 396.1402 | 395.133 |
| 95 | Withanoside Ⅲ | 1.21 | 351.12191 | C_19_H_17_N_3_O_4_ | 351.1213 | 396.1195 |
| 96 | Malonyl-ginsenoside Rb1 | 1.22 | 247.14197 | C_11_H_21_NO_5_ | 247.1431 | 292.1413 |
| 97 | Neogermitrine | 1.23 | 208.12118 | C_11_H_16_N_2_O_2_ | 208.1217 | 209.129 |
| 98 | D-Phenylalanine | 1.27 | 304.07943 | C_12_H_16_O_9_ | 304.0809 | 303.0736 |
| 99 | Eleutherazine B | 1.28 | 224.14124 | C_13_H_20_O_3_ | 224.1402 | 247.1295 |
| 100 | Tunicyclin A | 1.28 | 117.07898 | C_5_H_11_NO_2_ | 117.0786 | 118.0859 |
| 101 | Withametelin E | 1.28 | 333.10011 | C_20_H_15_NO_4_ | 333.0997 | 378.0979 |
| 102 | Kukoamine A | 1.3 | 218.16707 | C_15_H_22_O | 218.1661 | 241.1553 |
| 103 | Prosapogenin 3 | 1.3 | 504.16316 | C_25_H_28_O_11_ | 504.1646 | 503.1574 |
| 104 | 1,2,4-Triethyl benzene | 1.32 | 206.16707 | C_14_H_22_O | 206.1661 | 229.1554 |
| 105 | Anginin | 1.32 | 151.04941 | C_5_H_5_N_5_O | 151.0499 | 150.0426 |
| 106 | Cimicifugoside H2 | 1.32 | 686.2422 | C_31_H_42_O_17_ | 686.2425 | 731.2407 |
| 107 | Andrographatoside | 1.33 | 151.04941 | C_5_H_5_N_5_O | 151.0497 | 152.0569 |
| 108 | Biatractylolide | 1.35 | 136.03851 | C_5_H_4_N_4_O | 136.0387 | 137.046 |
| 109 | Carthamin | 1.36 | 268.08077 | C_10_H_12_N_4_O_5_ | 268.0817 | 267.0744 |
| 110 | BidentatosideⅠ | 1.36 | 136.03851 | C_5_H_4_N_4_O | 136.0386 | 135.0313 |
| 111 | Ganoderic acid α | 1.37 | 574.12638 | C_34_H_22_O_9_ | 574.1272 | 573.1199 |
| 112 | 5-Hydroxyindole-3-acetic acid | 1.37 | 756.24768 | C_34_H_44_O_19_ | 756.2477 | 755.2404 |
| 113 | Worenine | 1.38 | 299.07937 | C_16_H_13_NO_5_ | 299.078 | 298.0707 |
| 114 | Dihydrocoumarin | 1.42 | 342.11621 | C_12_H_22_O_11_ | 342.1175 | 387.1157 |
| 115 | Luteolin-7,4'-di-O-β-D-glucopyranoside | 1.42 | 666.22186 | C_24_H_42_O_21_ | 666.2233 | 711.2215 |
| 116 | Lyciumin C | 1.46 | 164.04734 | C_9_H_8_O_3_ | 164.0478 | 165.0551 |
| 117 | Methyl arteannuate | 1.58 | 562.16057 | C_28_H_31_ClO_10_ | 562.1608 | 607.159 |
| 118 | Ganoderic acid H | 1.58 | 562.16277 | C_34_H_26_O_8_ | 562.1608 | 607.159 |
| 119 | Acankoreoside M | 1.59 | 165.07898 | C_9_H_11_NO_2_ | 165.0792 | 166.0865 |
| 120 | (3R,4R,6S)-3,4-Epoxybisabola-7(14),10-dien-2-one | 1.59 | 148.05243 | C_9_H_8_O_2_ | 148.0528 | 149.0601 |
| 121 | Matol-β-D-glucopyranoside | 1.59 | 165.07898 | C_9_H_11_NO_2_ | 165.0795 | 210.0777 |
| 122 | Khellol-β-D-glucoside | 1.61 | 151.06333 | C_8_H_9_NO_2_ | 151.0626 | 152.0699 |
| 123 | 6'-O-β-D-Glucosylsweroside | 1.67 | 149.08406 | C_9_H_11_NO | 149.0842 | 150.0915 |
| 124 | D-Fructose-6-phosphate | 1.68 | 342.11621 | C_12_H_22_O_11_ | 342.1177 | 387.1159 |
| 125 | Indole-3-carboxylic acid | 1.68 | 504.16903 | C_18_H_32_O_16_ | 504.1706 | 503.1633 |
| 126 | Kingianoside B | 1.73 | 151.06333 | C_8_H_9_NO_2_ | 151.063 | 152.0703 |
| 127 | 7β-(3-Ethyl-cis-crotonoyloxy)-14-hydroxynotonipetranone | 1.73 | 193.07389 | C_10_H_11_NO_3_ | 193.0747 | 194.0819 |
| 128 | Acanthosessilioside F | 1.74 | 193.07389 | C_10_H_11_NO_3_ | 193.0742 | 192.0669 |
| 129 | 9-Ene-methyl palmitate | 1.74 | 193.07389 | C_10_H_11_NO_3_ | 193.0742 | 192.0669 |
| 130 | Daturametelin G | 1.76 | 291.18344 | C_17_H_25_NO_3_ | 291.1825 | 314.1717 |
| 131 | Val | 1.79 | 224.14124 | C_13_H_20_O_3_ | 224.1403 | 247.1295 |
| 132 | Scutellone E | 1.79 | 430.22028 | C_21_H_34_O_9_ | 430.218 | 475.2162 |
| 133 | Xanthine | 1.86 | 284.07568 | C_10_H_12_N_4_O_6_ | 284.0769 | 283.0696 |
| 134 | (E)-3,3'-Dihydroxy-4,4-dimethoxystilbene | 1.86 | 177.07898 | C_10_H_11_NO_2_ | 177.0795 | 176.0722 |
| 135 | (6′-O-Palmitoyl)-sitosterol-3-O-β-D-glucoside | 1.87 | 177.07898 | C_10_H_11_NO_2_ | 177.0795 | 178.0868 |
| 136 | 17-O-β-D-Glucopyra-nosyl-16β-H-ent-kauran-19-oic acid-19-O-β-D-glucopyranoside | 1.9 | 174.11168 | C_6_H_14_N_4_O_2_ | 174.1121 | 175.1194 |
| 137 | Diayangambin | 1.9 | 342.11621 | C_12_H_22_O_11_ | 342.1176 | 387.1158 |
| 138 | Ricinoleic acid | 1.9 | 329.16271 | C_19_H_23_NO_4_ | 329.161 | 374.1592 |
| 139 | Luteolin-7-O-[β-D-apiofuranosyl(1→6)]β-D-glucopyranoside | 1.99 | 666.22186 | C_24_H_42_O_21_ | 666.2232 | 665.2159 |
| 140 | D-Fructose-1,6-bisphosphate | 1.99 | 342.11621 | C_12_H_22_O_11_ | 342.1168 | 341.1096 |
| 141 | Dihydroxy stearic acid | 2 | 504.16903 | C_18_H_32_O_16_ | 504.1707 | 503.1634 |
| 142 | Elaeocarpusin | 2.01 | 224.14124 | C_13_H_20_O_3_ | 224.1402 | 247.1294 |
| 143 | Galphimin A | 2.07 | 548.15299 | C_26_H_28_O_13_ | 548.151 | 547.1438 |
| 144 | 2-Aminoacetophenone | 2.08 | 165.07898 | C_9_H_11_NO_2_ | 165.0789 | 164.0716 |
| 145 | Sanleng acid | 2.08 | 104.0626 | C_8_H_8_ | 104.0625 | 103.0552 |
| 146 | 25R-Inokosterone | 2.1 | 165.07898 | C_9_H_11_NO_2_ | 165.0791 | 166.0863 |
| 147 | (3α,4β,5α)-4,5-Dihydro-4,5-dimethyl-3-(1-pyrrolyl)-furan-2(3H)-one | 2.1 | 148.05243 | C_9_H_8_O_2_ | 148.0527 | 149.0599 |
| 148 | 23-Acetate alisol J | 2.12 | 219.11067 | C_9_H_17_NO_5_ | 219.1107 | 218.1034 |
| 149 | 4-Hydroxymethyl-γ-butyrolactone | 2.12 | 147.08954 | C_6_H_13_NO_3_ | 147.0897 | 146.0824 |
| 150 | 20-Hydroxyecdysone | 2.13 | 219.11067 | C_9_H_17_NO_5_ | 219.111 | 220.1183 |
| 151 | Akeboside Stf | 2.17 | 151.04941 | C_5_H_5_N_5_O | 151.0502 | 152.0574 |
| 152 | Quinatoside B | 2.22 | 366.19166 | C_19_H_28_NO_6_ | 366.1915 | 367.1988 |
| 153 | o-Coumaric acid | 2.22 | 418.25667 | C_21_H_38_O_8_ | 418.2564 | 441.2456 |
| 154 | Tenacissoside F | 2.22 | 242.09027 | C_10_H_14_N_2_O_5_ | 242.0913 | 241.0841 |
| 155 | 2-Hydroxybenzyl alcohol | 2.23 | 484.25331 | C_22_H_36_N_4_O_8_ | 484.255 | 485.2623 |
| 156 | Atroposide E | 2.23 | 270.17321 | C_17_H_22_N_2_O | 270.1729 | 293.1622 |
| 157 | Quinatoside A | 2.24 | 423.26209 | C_23_H_37_NO_6_ | 423.2607 | 446.25 |
| 158 | Melianol | 2.26 | 232.08479 | C_12_H_12_N_2_O_3_ | 232.0858 | 277.084 |
| 159 | ApocynosideⅡ | 2.27 | 368.20999 | C_22_H_28_N_2_O_3_ | 368.2096 | 391.1988 |
| 160 | Myristoleic acid methyl ester | 2.27 | 614.29384 | C_30_H_46_O_13_ | 614.2956 | 659.2938 |
| 161 | Tenacissoside M | 2.28 | 220.14633 | C_14_H_20_O_2_ | 220.1455 | 243.1347 |
| 162 | Daturametelin I | 2.28 | 247.15723 | C_15_H_21_NO_2_ | 247.1584 | 270.1476 |
| 163 | Nimbolidin B | 2.28 | 115.06333 | C_5_H_9_NO_2_ | 115.063 | 116.0702 |
| 164 | 2″-O-Rhamnosyl vitexin | 2.29 | 197.06881 | C_9_H_11_NO_4_ | 197.0696 | 198.0769 |
| 165 | Atroposide F | 2.3 | 310.12051 | C_19_H_18_O_4_ | 310.1192 | 311.1265 |
| 166 | Nigakilactone I | 2.3 | 115.06333 | C_5_H_9_NO_2_ | 115.0635 | 114.0562 |
| 167 | Oleanane-glucopyranosiduronic acid deriv-1 | 2.31 | 400.2461 | C_21_H_36_O_7_ | 400.2459 | 423.2352 |
| 168 | Neogermbudine | 2.31 | 208.12118 | C_11_H_16_N_2_O_2_ | 208.1217 | 209.129 |
| 169 | Smiglaside D | 2.32 | 398.17294 | C_23_H_26_O_6_ | 398.174 | 399.1812 |
| 170 | 6-Hydroxykynurenic acid | 2.33 | 572.29853 | C_32_H_44_O_9_ | 572.2965 | 595.2857 |
| 171 | 2,4-Dihydroxyphenylacetic acid methylester | 2.34 | 181.07389 | C_9_H_11_NO_3_ | 181.0741 | 180.0668 |
| 172 | (6S)-2-Methyl-6-[(1R,5S)-(4-methene-5-hydroxyl-2-cyclohexen)-2-hepten-4-one] | 2.34 | 177.07898 | C_10_H_11_NO_2_ | 177.0796 | 222.0778 |
| 173 | N8-Feruloyl spermidine | 2.35 | 572.28328 | C_28_H_44_O_12_ | 572.2824 | 617.2806 |
| 174 | Tracheloionoside | 2.36 | 160.10005 | C_10_H_12_N_2_ | 160.0994 | 161.1067 |
| 175 | Indole | 2.39 | 376.18859 | C_21_H_28_O_6_ | 376.19 | 377.1973 |
| 176 | Riboflavin (Vitamin B2) | 2.4 | 488.26215 | C_24_H_40_O_10_ | 488.2601 | 487.2529 |
| 177 | Pseudolaric acid A O-β-D-glucopyranoside | 2.41 | 719.36426 | C_37_H_49_N_7_O_8_ | 719.3625 | 742.3517 |
| 178 | 23-Acetate alisomalactone | 2.42 | 219.11067 | C_9_H_17_NO_5_ | 219.1108 | 218.1035 |
| 179 | 22-Hydroxychiisanoside | 2.42 | 219.11067 | C_9_H_17_NO_5_ | 219.1112 | 220.1185 |
| 180 | 4-Hydroxyquinoline | 2.42 | 147.08954 | C_6_H_13_NO_3_ | 147.0898 | 146.0825 |
| 181 | Terrestrosin I | 2.44 | 516.25706 | C_25_H_40_O_11_ | 516.2577 | 517.265 |
| 182 | Courmaric acid | 2.45 | 462.22537 | C_25_H_34_O_8_ | 462.2259 | 463.2331 |
| 183 | Dihydro-khusilol | 2.46 | 504.16903 | C_18_H_32_O_16_ | 504.1676 | 503.1603 |
| 184 | Germine | 2.5 | 624.31994 | C_38_H_44_N_2_O_6_ | 624.3206 | 625.3278 |
| 185 | Gentiatibetine | 2.52 | 321.20524 | C_17_H_27_N_3_O_3_ | 321.2041 | 344.1933 |
| 186 | Neohecogenin-3-O-β-D-glucopyranoside | 2.52 | 208.12118 | C_11_H_16_N_2_O_2_ | 208.1223 | 253.1205 |
| 187 | Hoodistanaloside A | 2.53 | 162.1157 | C_10_H_14_N_2_ | 162.116 | 163.1232 |
| 188 | Trifoside C | 2.54 | 667.31771 | C_29_H_45_N_7_O_11_ | 667.3149 | 668.3222 |
| 189 | Psammosilenins B | 2.54 | 609.32748 | C_31_H_43_N_7_O_6_ | 609.3274 | 632.3167 |
| 190 | Cynatratoside B | 2.54 | 528.27232 | C_30_H_40_O_8_ | 528.2733 | 529.2806 |
| 191 | Calycanthoside | 2.54 | 161.04768 | C_9_H_7_NO_2_ | 161.048 | 160.0407 |
| 192 | Bullatantriol | 2.55 | 161.04768 | C_9_H_7_NO_2_ | 161.0478 | 162.0551 |
| 193 | N,N,N-Trimethyltry-ptophan | 2.55 | 572.28328 | C_28_H_44_O_12_ | 572.2859 | 595.2751 |
| 194 | 19-O-[β-D-Apiofuran-osyl(1→2)-β-D-gluco-pyranoyl]-3,14-dideo-xyandrographolide | 2.55 | 174.11168 | C_6_H_14_N_4_O_2_ | 174.1124 | 175.1196 |
| 195 | Magnaldehyde B | 2.55 | 431.1944 | C_23_H_29_NO_7_ | 431.194 | 432.2013 |
| 196 | Scutellone H | 2.56 | 515.29167 | C_26_H_45_NO_7_S | 515.2918 | 538.2811 |
| 197 | Pennogenin-3-O-α-L-arabinofuranosyl(1→4)[α-L-rhamnopyranosyl(1→2)]-β-D-glucopyranoside | 2.56 | 154.06299 | C_8_H_10_O_3_ | 154.0635 | 153.0563 |
| 198 | (+)-Tubocurarine | 2.57 | 446.19407 | C_24_H_30_O_8_ | 446.1961 | 447.2034 |
| 199 | Cyclo(Pro-Ala) | 2.57 | 115.06333 | C_5_H_9_NO_2_ | 115.063 | 116.0703 |
| 200 | 2,4,5-Trimethoxybenzoic acid | 2.57 | 181.07389 | C_9_H_11_NO_3_ | 181.0743 | 180.067 |
| 201 | N-Acetyl-glutamic acid | 2.58 | 572.28328 | C_28_H_44_O_12_ | 572.2827 | 617.2809 |
| 202 | (E,E)-9-Oxooctadeca-10,12-dienoic acid | 2.59 | 206.16707 | C_14_H_22_O | 206.1661 | 229.1554 |
| 203 | 12-Methyl-tetradeca-noic acid | 2.61 | 326.15181 | C_20_H_22_O_4_ | 326.1518 | 349.141 |
| 204 | 2S,4S-4-Hydroxy proline | 2.61 | 546.31927 | C_31_H_46_O_8_ | 546.3216 | 591.3198 |
| 205 | Cistanoside F | 2.61 | 384.15729 | C_22_H_24_O_6_ | 384.1581 | 429.1563 |
| 206 | 1α,7β-Di(4-methylse-necioyloxy)oplopa-3(14)Z,8(10)-dien-2-one | 2.63 | 204.08988 | C_11_H_12_N_2_O_2_ | 204.0907 | 203.0834 |
| 207 | Tribulosin | 2.63 | 160.10005 | C_10_H_12_N_2_ | 160.1003 | 159.093 |
| 208 | Bufotalinin | 2.63 | 117.05785 | C_8_H_7_N | 117.058 | 116.0507 |
| 209 | 1-Monopalmitin | 2.65 | 204.08988 | C_11_H_12_N_2_O_2_ | 204.0906 | 205.0979 |
| 210 | Bufalin | 2.65 | 117.05785 | C_8_H_7_N | 117.0577 | 118.065 |
| 211 | 3S,5R,6R,9S-Tetrahydroxymegastigmane | 2.65 | 1150.54073 | C_54_H_86_O_26_ | 1150.5374 | 1173.5266 |
| 212 | 2,4,5-Trihydeoxybenzaldehyde | 2.65 | 181.07389 | C_9_H_11_NO_3_ | 181.0742 | 180.0669 |
| 213 | Cistanoside H | 2.69 | 384.15729 | C_22_H_24_O_6_ | 384.158 | 429.1562 |
| 214 | Ornithine | 2.7 | 534.26309 | C_33_H_34_N_4_O_3_ | 534.2607 | 579.2589 |
| 215 | Ile | 2.71 | 424.20972 | C_22_H_32_O_8_ | 424.2087 | 469.2069 |
| 216 | Schizonepetoside E | 2.71 | 498.28288 | C_26_H_42_O_9_ | 498.2804 | 497.2732 |
| 217 | Canrenone | 2.72 | 161.04768 | C_9_H_7_NO_2_ | 161.0481 | 160.0408 |
| 218 | 5-Ene-methylcholate-3-O-β-D-glucuronopyranosyl-(1→4)-α-L-rhamnopyranoside | 2.74 | 290.05791 | C_18_H_10_O_4_ | 290.0565 | 335.0547 |
| 219 | Siraitic acid A | 2.74 | 874.4926 | C_44_H_74_O_17_ | 874.4967 | 873.4894 |
| 220 | Zanthobungeanine | 2.74 | 271.12084 | C_16_H_17_NO_3_ | 271.1212 | 270.1139 |
| 221 | YemuosideⅠ | 2.75 | 271.12084 | C_16_H_17_NO_3_ | 271.1217 | 272.129 |
| 222 | 3-O-β-D-Glucopyranosyl-14,19-dideoxyandrographolide | 2.75 | 1002.46717 | C_48_H_74_O_22_ | 1002.4712 | 1025.4604 |
| 223 | Terrestrosin G | 2.75 | 772.42452 | C_39_H_64_O_15_ | 772.4229 | 771.4156 |
| 224 | Vitetrifolin D | 2.75 | 486.26175 | C_28_H_38_O_7_ | 486.2608 | 531.259 |
| 225 | Pterodontoside F | 2.76 | 719.36426 | C_37_H_49_N_7_O_8_ | 719.3675 | 720.3747 |
| 226 | Terreside B | 2.76 | 772.42452 | C_39_H_64_O_15_ | 772.4221 | 773.4294 |
| 227 | Dehydrololiolide | 2.76 | 485.27774 | C_28_H_39_NO_6_ | 485.2754 | 484.2681 |
| 228 | Pennogenin-3-O-α-L-rhamnopyranosyl(1→2)-[α-L-rhamno-pyranosyl(1→4)]-β-D-glucopyranoside | 2.77 | 400.19982 | C_22_H_28_N_2_O_5_ | 400.1991 | 401.2064 |
| 229 | Quinatoside D | 2.77 | 209.06881 | C_10_H_11_NO_4_ | 209.0694 | 208.0621 |
| 230 | Chymostatin | 2.77 | 342.11621 | C_12_H_22_O_11_ | 342.1166 | 341.1093 |
| 231 | Magnolol | 2.78 | 238.15689 | C_14_H_22_O_3_ | 238.1565 | 261.1457 |
| 232 | Lablaboside A | 2.79 | 131.09463 | C_6_H_13_NO_2_ | 131.0945 | 132.1018 |
| 233 | ArteamisinineⅠ | 2.79 | 603.30435 | C_32_H_45_NO_10_ | 603.3033 | 626.2926 |
| 234 | Isonuezhenide | 2.79 | 686.33023 | C_37_H_50_O_12_ | 686.3272 | 685.32 |
| 235 | TrigoneosideⅠa | 2.8 | 506.28797 | C_28_H_42_O_8_ | 506.2876 | 529.2768 |
| 236 | Hordatine A | 2.8 | 410.23045 | C_22_H_34_O_7_ | 410.2318 | 433.221 |
| 237 | Mutongsaponin C | 2.8 | 598.29893 | C_30_H_46_O_12_ | 598.2997 | 599.307 |
| 238 | Hecogenin-3-O-β-D-xylopyransyl(1→2)-[β-D-xylopyransyl (1→3)]-β-D-glucopyranosyl(1→4)-[α-rhamnopyranosyl (1→2)]-β-D-mannopyranoside | 2.8 | 900.47187 | C_45_H_72_O_18_ | 900.4692 | 899.4619 |
| 239 | Psamosilenins A | 2.81 | 609.32748 | C_31_H_43_N_7_O_6_ | 609.3283 | 632.3175 |
| 240 | 7-oxo-β-Sitosteryl tetra-O-acetyl-β-D-glycopyranoside | 2.82 | 149.08406 | C_9_H_11_NO | 149.0843 | 194.0825 |
| 241 | Acanthosessiligenin II | 2.82 | 193.07389 | C_10_H_11_NO_3_ | 193.0743 | 192.067 |
| 242 | Ciwujianoside B | 2.84 | 660.33571 | C_32_H_52_O_14_ | 660.3372 | 683.3264 |
| 243 | Daturametelin E | 2.84 | 467.2883 | C_25_H_41_NO_7_ | 467.2867 | 490.276 |
| 244 | Cartormin | 2.84 | 371.20966 | C_22_H_29_NO_4_ | 371.2105 | 394.1997 |
| 245 | Nigakihemiacetal C | 2.85 | 768.39322 | C_39_H_60_O_15_ | 768.3918 | 791.381 |
| 246 | (E)-9-Octadecenoic acid | 2.85 | 177.07898 | C_10_H_11_NO_2_ | 177.0798 | 222.078 |
| 247 | Trifoside B | 2.87 | 667.31771 | C_29_H_45_N_7_O_11_ | 667.3169 | 668.3242 |
| 248 | Turpinionosides B | 2.87 | 117.07898 | C_5_H_11_NO_2_ | 117.0794 | 116.0721 |
| 249 | 6-Hydroxymelatonin | 2.88 | 572.29853 | C_32_H_44_O_9_ | 572.297 | 573.3042 |
| 250 | Quinatoside C | 2.88 | 295.17836 | C_16_H_25_NO_4_ | 295.1776 | 318.1668 |
| 251 | Daphnoretin | 2.88 | 941.42832 | C_47_H_59_N_9_O_12_ | 941.431 | 942.4383 |
| 252 | Kusulactone | 2.9 | 166.13577 | C_11_H_18_O | 166.1348 | 189.1241 |
| 253 | Viscumneoside Ⅲ | 2.91 | 189.04259 | C_10_H_7_NO_3_ | 189.0428 | 188.0355 |
| 254 | Veratetrine | 2.92 | 189.04259 | C_10_H_7_NO_3_ | 189.0433 | 190.0506 |
| 255 | D-ribose-5-phosphate | 2.92 | 149.05105 | C_5_H_11_NO_2_S | 149.0507 | 172.0399 |
| 256 | Bulnesol | 2.92 | 161.04768 | C_9_H_7_NO_2_ | 161.0483 | 162.0556 |
| 257 | Blumenol C glucoside | 2.93 | 131.09463 | C_6_H_13_NO_2_ | 131.0949 | 130.0876 |
| 258 | Huperzinine | 2.96 | 410.23045 | C_22_H_34_O_7_ | 410.231 | 433.2202 |
| 259 | Isoinosine | 2.97 | 698.33023 | C_38_H_50_O_12_ | 698.3324 | 699.3397 |
| 260 | Pro | 2.97 | 732.35096 | C_42_H_52_O_11_ | 732.3538 | 731.3465 |
| 261 | Nigakihemiacetal A | 2.99 | 898.45622 | C_45_H_70_O_18_ | 898.4518 | 921.4411 |
| 262 | Gentianine | 3 | 246.13683 | C_14_H_18_N_2_O_2_ | 246.1373 | 247.1446 |
| 263 | 1,1,6-Trimethyl-1,2-dihydronaphthalene | 3.01 | 206.16707 | C_14_H_22_O | 206.166 | 229.1552 |
| 264 | Acankoreoside N | 3.01 | 165.07898 | C_9_H_11_NO_2_ | 165.0797 | 166.0869 |
| 265 | Wilsonic acid | 3.03 | 652.34588 | C_34_H_52_O_12_ | 652.3482 | 675.3374 |
| 266 | Nuciferine | 3.03 | 550.24141 | C_28_H_38_O_11_ | 550.2418 | 551.2491 |
| 267 | Nystose | 3.03 | 550.24141 | C_28_H_38_O_11_ | 550.2402 | 549.2329 |
| 268 | Prosapogenin 4 | 3.03 | 452.21989 | C_27_H_32_O_6_ | 452.2184 | 453.2257 |
| 269 | 1-Monomethyl citrate | 3.04 | 204.08988 | C_11_H_12_N_2_O_2_ | 204.0903 | 205.0976 |
| 270 | Cyclo(Pro-Val) | 3.04 | 115.06333 | C_5_H_9_NO_2_ | 115.0633 | 116.0705 |
| 271 | Pregn-5-en-20-one deriv P57 | 3.04 | 382.23554 | C_21_H_34_O_6_ | 382.2345 | 405.2238 |
| 272 | Daturametelin J | 3.05 | 485.27774 | C_28_H_39_NO_6_ | 485.2773 | 486.2846 |
| 273 | Dendronobilin B | 3.05 | 280.10994 | C_18_H_16_O_3_ | 280.1096 | 325.1078 |
| 274 | Cynanoside Q3 | 3.06 | 311.14812 | C_14_H_21_N_3_O_5_ | 311.1497 | 356.1479 |
| 275 | Cimiside C | 3.07 | 482.26684 | C_29_H_38_O_6_ | 482.2678 | 505.257 |
| 276 | Rhapontisterone C | 3.07 | 462.2465 | C_22_H_38_O_10_ | 462.2481 | 507.2463 |
| 277 | Trifoside A | 3.08 | 297.13649 | C_18_H_19_NO_3_ | 297.1357 | 320.1249 |
| 278 | Segetalin E | 3.09 | 814.41396 | C_44_H_62_O_14_ | 814.4103 | 837.3995 |
| 279 | BOA | 3.09 | 131.09463 | C_6_H_13_NO_2_ | 131.095 | 130.0877 |
| 280 | Neojiangyouaconitine | 3.1 | 456.10565 | C_23_H_20_O_10_ | 456.1058 | 455.0985 |
| 281 | Neohecogenin-3-O-β-D-glucopyranosyl(1→2)-β-D-glucopyranosyl (1→4)-β-D-galactopy-ranoside | 3.1 | 456.10565 | C_23_H_20_O_10_ | 456.1061 | 457.1134 |
| 282 | 3β-Formyloxy-7β,12β-dihydroxy-5α-lanost-11,15,23-trioxo-8-en-(E)-26-oic acid | 3.1 | 157.07389 | C_7_H_11_NO_3_ | 157.0745 | 202.0727 |
| 283 | Cynanoside R1 | 3.11 | 610.30429 | C_37_H_42_N_2_O_6_ | 610.3026 | 611.3099 |
| 284 | Cynatratoside D | 3.11 | 518.28797 | C_29_H_42_O_8_ | 518.2855 | 541.2747 |
| 285 | Senbusine B | 3.11 | 1016.49808 | C_53_H_76_O_19_ | 1016.5027 | 1015.4955 |
| 286 | Cimifoetiside Ⅳ | 3.12 | 152.09496 | C_8_H_12_N_2_O | 152.0954 | 197.0936 |
| 287 | Oxyphyllenodiol A | 3.14 | 750.41904 | C_40_H_62_O_13_ | 750.419 | 773.4082 |
| 288 | Chavicol-β-D-glucoside | 3.14 | 371.20966 | C_22_H_29_NO_4_ | 371.2097 | 394.1989 |
| 289 | Rengyolone | 3.14 | 448.23085 | C_21_H_36_O_10_ | 448.2311 | 447.2238 |
| 290 | Rengynic acid | 3.15 | 448.23085 | C_21_H_36_O_10_ | 448.2323 | 449.2396 |
| 291 | N-Isobutyl-(2E,4E)-octadecadienamide | 3.17 | 793.42486 | C_41_H_63_NO_14_ | 793.4282 | 816.4174 |
| 292 | 5-Methyl-2-isopropyl-methylphenyl ether | 3.17 | 532.30362 | C_30_H_44_O_8_ | 532.3035 | 555.2927 |
| 293 | Pterodontoside H | 3.18 | 719.36426 | C_37_H_49_N_7_O_8_ | 719.3669 | 718.3596 |
| 294 | Hirsutine | 3.19 | 601.32508 | C_33_H_47_NO_9_ | 601.3275 | 624.3168 |
| 295 | Tribulusamide B | 3.19 | 387.20457 | C_22_H_29_NO_5_ | 387.2046 | 410.1938 |
| 296 | Borneol 2-O-β-D-apiosyl-(1→6)-β-D-glucoside | 3.19 | 131.09463 | C_6_H_13_NO_2_ | 131.0949 | 130.0876 |
| 297 | Shionoside B | 3.19 | 1018.51373 | C_53_H_78_O_19_ | 1018.5175 | 1063.5157 |
| 298 | D-(+)-Trehalose | 3.19 | 580.14282 | C_26_H_28_O_15_ | 580.1447 | 579.1374 |
| 299 | Trigoneoside Ⅲa | 3.2 | 390.22537 | C_19_H_34_O_8_ | 390.2275 | 435.2257 |
| 300 | 3β-O-trans-p-Coumaroyl alphitolic acid | 3.2 | 157.07389 | C_7_H_11_NO_3_ | 157.0746 | 202.0728 |
| 301 | Marsdenoside B | 3.2 | 494.32435 | C_28_H_46_O_7_ | 494.322 | 539.3202 |
| 302 | Ciwujianoside D3 | 3.21 | 736.40339 | C_39_H_60_O_13_ | 736.4011 | 759.3903 |
| 303 | Notoginsenoside G | 3.22 | 916.48471 | C_51_H_64_N_8_O_8_ | 916.4886 | 961.4869 |
| 304 | Esculentoside M | 3.23 | 548.27874 | C_34_H_36_N_4_O_3_ | 548.2762 | 593.2744 |
| 305 | Tenacissimoside B | 3.24 | 311.15214 | C_19_H_21_NO_3_ | 311.1512 | 334.1405 |
| 306 | 1-Methyluric acid | 3.24 | 204.08988 | C_11_H_12_N_2_O_2_ | 204.0902 | 205.0974 |
| 307 | Isopropyl methoxy pyrazine | 3.24 | 726.36153 | C_40_H_54_O_12_ | 726.3599 | 725.3526 |
| 308 | Serratanidine | 3.25 | 996.52938 | C_51_H_80_O_19_ | 996.5255 | 1041.5237 |
| 309 | Delbrusine | 3.26 | 1106.5509 | C_53_H_86_O_24_ | 1106.5468 | 1129.536 |
| 310 | Citronellyl formate | 3.26 | 580.14282 | C_26_H_28_O_15_ | 580.1449 | 579.1377 |
| 311 | Butyl isobutyl phthalate | 3.26 | 161.04768 | C_9_H_7_NO_2_ | 161.0485 | 206.0467 |
| 312 | Phytolaccoside D | 3.27 | 489.26276 | C_29_H_35_N_3_O_4_ | 489.2614 | 490.2687 |
| 313 | Atratoside A | 3.27 | 550.3016 | C_28_H_38_N_8_O_4_ | 550.2991 | 551.3064 |
| 314 | Marsdenoside A | 3.27 | 696.37209 | C_36_H_56_O_13_ | 696.3712 | 719.3604 |
| 315 | Malonyl-ginsenoside Rd | 3.27 | 564.14791 | C_26_H_28_O_14_ | 564.1495 | 563.1423 |
| 316 | 2α,3β,4α-Trihydroxy nortropane | 3.28 | 826.43509 | C_42_H_66_O_16_ | 826.4322 | 849.4214 |
| 317 | Marsdenoside C | 3.29 | 494.32435 | C_28_H_46_O_7_ | 494.3226 | 539.3208 |
| 318 | Isomaltose | 3.29 | 698.33023 | C_38_H_50_O_12_ | 698.3319 | 697.3246 |
| 319 | Ethyl succinate | 3.3 | 908.48952 | C_47_H_68_N_6_O_12_ | 908.4929 | 953.4911 |
| 320 | Tussilagolactone | 3.3 | 809.41977 | C_41_H_63_NO_15_ | 809.4162 | 832.4054 |
| 321 | Clavatine | 3.31 | 932.46169 | C_45_H_72_O_20_ | 932.4594 | 955.4486 |
| 322 | 4-Methyl ester octenoic acid | 3.31 | 608.28864 | C_37_H_40_N_2_O_6_ | 608.2885 | 607.2812 |
| 323 | Periplocin | 3.32 | 376.13828 | C_17_H_20_N_4_O_6_ | 376.1396 | 375.1323 |
| 324 | Pennogenin-3-O-α-L-rhamnopyranosyl(1→4)-O-α-L-rhamnopyranosyl (1→4)-[α-L-rhamnopy-ranosyl-(1→2)]-β-D-glucopyrano side | 3.33 | 376.13828 | C_17_H_20_N_4_O_6_ | 376.1392 | 377.1465 |
| 325 | 6-Gingerol | 3.33 | 510.26175 | C_30_H_38_O_7_ | 510.2636 | 511.2709 |
| 326 | Tenacissoside E | 3.33 | 242.1089 | C_11_H_18_N_2_O_2_S | 242.1077 | 287.1059 |
| 327 | Dipetaline | 3.33 | 936.50825 | C_49_H_76_O_17_ | 936.5117 | 981.5099 |
| 328 | 2-(p-Anisyl)-5-methyl-1-hexen | 3.34 | 181.07389 | C_9_H_11_NO_3_ | 181.0747 | 182.082 |
| 329 | Daturametelin A | 3.34 | 941.42832 | C_47_H_59_N_9_O_12_ | 941.4327 | 986.4309 |
| 330 | Ophiopogonin B' | 3.34 | 471.22302 | C_22_H_29_N_7_O_5_ | 471.221 | 516.2192 |
| 331 | Gracillin | 3.35 | 677.37751 | C_36_H_55_NO_11_ | 677.379 | 700.3683 |
| 332 | p-Tolualdehyde | 3.35 | 812.42211 | C_43_H_56_N_8_O_8_ | 812.4212 | 835.4104 |
| 333 | Dendronobilin F | 3.35 | 266.13068 | C_18_H_18_O_2_ | 266.1301 | 289.1193 |
| 334 | Liriodenine | 3.35 | 960.52938 | C_48_H_80_O_19_ | 960.527 | 983.5162 |
| 335 | Astin G | 3.35 | 1124.56147 | C_53_H_88_O_25_ | 1124.5603 | 1169.5585 |
| 336 | Daturametelin H | 3.36 | 291.18344 | C_17_H_25_NO_3_ | 291.1824 | 314.1717 |
| 337 | Cytosine | 3.36 | 610.15338 | C_27_H_30_O_16_ | 610.1537 | 655.1519 |
| 338 | Mannotriose | 3.37 | 738.41904 | C_39_H_62_O_13_ | 738.4164 | 761.4057 |
| 339 | Isopentenyladenine | 3.37 | 726.36153 | C_40_H_54_O_12_ | 726.3623 | 727.3696 |
| 340 | 6-Methoxyharmalan | 3.37 | 574.31418 | C_32_H_46_O_9_ | 574.3136 | 597.3029 |
| 341 | Kushecarpins C | 3.37 | 166.13577 | C_11_H_18_O | 166.135 | 189.1242 |
| 342 | Acanjaposide C | 3.37 | 175.06333 | C_10_H_9_NO_2_ | 175.0643 | 220.0625 |
| 343 | Jujuboside B | 3.38 | 738.36153 | C_41_H_54_O_12_ | 738.3626 | 739.3699 |
| 344 | DL-Tryptophan | 3.38 | 766.41396 | C_40_H_62_O_14_ | 766.4153 | 789.4045 |
| 345 | Nimbolidin C | 3.38 | 115.06333 | C_5_H_9_NO_2_ | 115.0632 | 116.0705 |
| 346 | Marsdenoside E | 3.38 | 494.32435 | C_28_H_46_O_7_ | 494.3231 | 539.3213 |
| 347 | DL-Arginine | 3.38 | 856.46091 | C_47_H_68_O_14_ | 856.4645 | 855.4573 |
| 348 | Delamide | 3.39 | 263.18853 | C_16_H_25_NO_2_ | 263.188 | 286.1772 |
| 349 | Ciwujianoside D2 | 3.39 | 736.40339 | C_39_H_60_O_13_ | 736.4055 | 759.3947 |
| 350 | Scuterivulactone A | 3.4 | 515.29167 | C_26_H_45_NO_7_S | 515.2932 | 560.2914 |
| 351 | Fagomine | 3.41 | 1001.51099 | C_52_H_71_N_7_O_13_ | 1001.514 | 1046.5122 |
| 352 | Succinic acid | 3.42 | 1098.54582 | C_51_H_86_O_25_ | 1098.5404 | 1121.5296 |
| 353 | n-Heptadecanal | 3.42 | 898.45622 | C_45_H_70_O_18_ | 898.4531 | 921.4423 |
| 354 | Prosapogenin 5 (Julibroside A1) | 3.43 | 498.26175 | C_29_H_38_O_7_ | 498.2611 | 499.2684 |
| 355 | Dopal | 3.44 | 766.41396 | C_40_H_62_O_14_ | 766.4155 | 789.4047 |
| 356 | 5-Oxoproline | 3.44 | 616.32475 | C_34_H_48_O_10_ | 616.322 | 615.3147 |
| 357 | Azedarachin C | 3.45 | 615.30435 | C_33_H_45_NO_10_ | 615.3013 | 616.3086 |
| 358 | Delbruline | 3.45 | 1064.54034 | C_51_H_84_O_23_ | 1064.5368 | 1065.5441 |
| 359 | 2-Monolinolein | 3.45 | 561.29512 | C_31_H_39_N_5_O_5_ | 561.2955 | 562.3027 |
| 360 | Teracrylshikonin | 3.45 | 220.14633 | C_14_H_20_O_2_ | 220.1451 | 243.1344 |
| 361 | Isosamarcandin | 3.46 | 726.36153 | C_40_H_54_O_12_ | 726.3617 | 771.3599 |
| 362 | Harmalol | 3.47 | 900.47187 | C_45_H_72_O_18_ | 900.4755 | 923.4647 |
| 363 | Macrostemonoside D | 3.47 | 896.47695 | C_46_H_72_O_17_ | 896.4727 | 919.4619 |
| 364 | Cynascyroside C | 3.47 | 458.26684 | C_27_H_38_O_6_ | 458.2688 | 481.258 |
| 365 | Xanthosine | 3.47 | 1028.51921 | C_51_H_80_O_21_ | 1028.5229 | 1051.5121 |
| 366 | Protogracillin | 3.47 | 512.24102 | C_29_H_36_O_8_ | 512.239 | 511.2317 |
| 367 | Reserpic acid | 3.48 | 462.2465 | C_22_H_38_O_10_ | 462.2466 | 463.2539 |
| 368 | Segetalin A | 3.49 | 814.41396 | C_44_H_62_O_14_ | 814.4156 | 815.4229 |
| 369 | Hydrotanshinone ⅡA | 3.49 | 380.21989 | C_21_H_32_O_6_ | 380.2214 | 403.2107 |
| 370 | 2H-1-Benzopyran-2-one | 3.49 | 642.36153 | C_33_H_54_O_12_ | 642.3601 | 643.3674 |
| 371 | Taraxacolide-1-O-β-D-glucopyranoside | 3.49 | 1080.53525 | C_51_H_84_O_24_ | 1080.5318 | 1125.53 |
| 372 | Palmitoleic acid methyl ester | 3.5 | 720.40848 | C_39_H_60_O_12_ | 720.4051 | 743.3943 |
| 373 | Pyrophaeophorbide A | 3.5 | 494.25158 | C_26_H_38_O_9_ | 494.2538 | 495.261 |
| 374 | Macrostemonoside G | 3.5 | 724.40339 | C_38_H_60_O_13_ | 724.4013 | 747.3905 |
| 375 | 2-Hydroxy-1-methoxyaporphine | 3.5 | 714.3649 | C_36_H_58_O_12_S | 714.363 | 759.3612 |
| 376 | Eucommiol | 3.51 | 985.51607 | C_52_H_71_N_7_O_12_ | 985.5153 | 1008.5046 |
| 377 | Sessiline | 3.51 | 1018.51373 | C_53_H_78_O_19_ | 1018.5185 | 1041.5077 |
| 378 | 11a-Hydroxyfawcettidine | 3.52 | 1078.5196 | C_51_H_82_O_24_ | 1078.5175 | 1079.5247 |
| 379 | Hokbusine A | 3.52 | 1004.51921 | C_49_H_80_O_21_ | 1004.5155 | 1027.5047 |
| 380 | Kadsurenin A | 3.52 | 674.3091 | C_39_H_46_O_10_ | 674.3099 | 673.3026 |
| 381 | 16-Oxo alisol A | 3.54 | 808.42452 | C_42_H_64_O_15_ | 808.4212 | 853.4194 |
| 382 | Bilirubin | 3.54 | 131.09463 | C_6_H_13_NO_2_ | 131.0952 | 130.0879 |
| 383 | Clinopodiside F | 3.55 | 530.31044 | C_28_H_42_N_4_O_6_ | 530.3082 | 553.2974 |
| 384 | 2-Amino-3-(3,4-dihydroxyphenyl)-propanoic acid methyl ester | 3.55 | 165.07898 | C_9_H_11_NO_2_ | 165.0795 | 164.0723 |
| 385 | Linolic acid | 3.55 | 127.09971 | C_7_H_13_NO | 127.1006 | 172.0988 |
| 386 | β-Dichroine (Febrifugine) | 3.55 | 301.14264 | C_16_H_19_N_3_O_3_ | 301.1432 | 346.1414 |
| 387 | Zhebeininoside | 3.55 | 593.39277 | C_33_H_55_NO_8_ | 593.3918 | 638.39 |
| 388 | Picrasidine C | 3.56 | 534.32061 | C_31_H_42_N_4_O_4_ | 534.3192 | 557.3084 |
| 389 | Nigakilactone H | 3.56 | 115.06333 | C_5_H_9_NO_2_ | 115.0633 | 116.0705 |
| 390 | Terrestrosin J | 3.56 | 516.25706 | C_25_H_40_O_11_ | 516.2559 | 561.2541 |
| 391 | 3,4-Dihydroxyphenethylamine | 3.57 | 826.43509 | C_42_H_66_O_16_ | 826.4343 | 849.4235 |
| 392 | Gomisin S | 3.57 | 677.37751 | C_36_H_55_NO_11_ | 677.375 | 700.3642 |
| 393 | Pennogenin-3-O-α-L-rhamnopyranosyl(1→2)-β-D-glucopyranoside | 3.57 | 508.34 | C_29_H_48_O_7_ | 508.3384 | 553.3366 |
| 394 | Puromycin | 3.58 | 812.42211 | C_43_H_56_N_8_O_8_ | 812.424 | 813.4313 |
| 395 | Glycoside K | 3.58 | 709.40373 | C_37_H_59_NO_12_ | 709.4011 | 732.3903 |
| 396 | Vomifoliol | 3.59 | 652.34588 | C_34_H_52_O_12_ | 652.3492 | 675.3384 |
| 397 | Neferine | 3.59 | 572.28328 | C_28_H_44_O_12_ | 572.2831 | 617.2813 |
| 398 | 4-Hydroxyacetophenone | 3.59 | 682.35644 | C_35_H_54_O_13_ | 682.3548 | 727.353 |
| 399 | DL-Tyrosine | 3.61 | 766.41396 | C_40_H_62_O_14_ | 766.4175 | 789.4068 |
| 400 | Hirsutolide | 3.61 | 601.32508 | C_33_H_47_NO_9_ | 601.3278 | 624.3171 |
| 401 | Apiin | 3.61 | 1164.55638 | C_55_H_88_O_26_ | 1164.5545 | 1165.5618 |
| 402 | Thiopental | 3.61 | 866.46639 | C_45_H_70_O_16_ | 866.4633 | 889.4525 |
| 403 | Mulberrofuran G | 3.61 | 810.44017 | C_42_H_66_O_15_ | 810.4377 | 809.4304 |
| 404 | Macrostemonoside K | 3.61 | 351.11067 | C_20_H_17_NO_5_ | 351.1119 | 396.1101 |
| 405 | D-pantothenic acid | 3.62 | 592.30362 | C_35_H_44_O_8_ | 592.3047 | 593.3119 |
| 406 | Thebaine | 3.62 | 912.47187 | C_46_H_72_O_18_ | 912.4714 | 935.4606 |
| 407 | 3-O-β-D-Galactopyra-nosyl-(1→2)-β-D-glucuronopyranosyl gypsogenin | 3.63 | 1134.54582 | C_54_H_86_O_25_ | 1134.5476 | 1135.5549 |
| 408 | Physanol A | 3.63 | 442.23554 | C_26_H_34_O_6_ | 442.2332 | 487.2314 |
| 409 | Aspidospermine | 3.64 | 603.30435 | C_32_H_45_NO_10_ | 603.3052 | 604.3124 |
| 410 | Phytolaccagenin | 3.64 | 489.26276 | C_29_H_35_N_3_O_4_ | 489.264 | 490.2713 |
| 411 | Timosaponin B-2 | 3.64 | 882.4613 | C_45_H_70_O_17_ | 882.4608 | 905.45 |
| 412 | Terrestrosin K | 3.65 | 1150.57712 | C_55_H_90_O_25_ | 1150.5812 | 1173.5704 |
| 413 | Marstenacigenin B | 3.68 | 604.36113 | C_34_H_52_O_9_ | 604.3584 | 627.3476 |
| 414 | Hexanorcucurbitacin F | 3.69 | 601.32508 | C_33_H_47_NO_9_ | 601.3245 | 602.3318 |
| 415 | 3-O-β-D-Galactopyranosyl-(1→2)-β-D-6-O-methylglucuronopyranosyl quillaic acid | 3.71 | 1018.49847 | C_49_H_78_O_22_ | 1018.4951 | 1019.5023 |
| 416 | 1-Nonen-3-ol | 3.72 | 204.08988 | C_11_H_12_N_2_O_2_ | 204.0906 | 203.0833 |
| 417 | Chelidimerine | 3.72 | 1114.55599 | C_55_H_86_O_23_ | 1114.5559 | 1159.5541 |
| 418 | 4,8-Dimethoxy-1-(2-methoxyethyl)-β-carboline | 3.72 | 174.08921 | C_8_H_14_O_4_ | 174.09 | 173.0828 |
| 419 | Gossypol | 3.74 | 677.37751 | C_36_H_55_NO_11_ | 677.378 | 700.3672 |
| 420 | Divaroside | 3.74 | 832.46091 | C_45_H_68_O_14_ | 832.4586 | 855.4478 |
| 421 | Acanthosessilioside C | 3.74 | 193.07389 | C_10_H_11_NO_3_ | 193.0745 | 192.0673 |
| 422 | Cyclo(Ala-Val) | 3.76 | 1102.55599 | C_54_H_86_O_23_ | 1102.5539 | 1125.5432 |
| 423 | Terrestrosin H | 3.76 | 1033.54576 | C_50_H_83_NO_21_ | 1033.5457 | 1056.5349 |
| 424 | 2''-O-Feruloylaloesin | 3.76 | 561.29512 | C_31_H_39_N_5_O_5_ | 561.2961 | 606.2943 |
| 425 | Taurocholic acid | 3.76 | 1078.5196 | C_51_H_82_O_24_ | 1078.5151 | 1077.5078 |
| 426 | Semiaquilegoside A | 3.77 | 1016.49808 | C_53_H_76_O_19_ | 1016.4989 | 1017.5062 |
| 427 | 2-Pentadecanone | 3.77 | 546.31927 | C_31_H_46_O_8_ | 546.317 | 591.3152 |
| 428 | Patuletin-7-O-[6′′-(2-methylbutyryl)]-glucoside | 3.77 | 174.08921 | C_8_H_14_O_4_ | 174.09 | 173.0828 |
| 429 | Aster saponin G | 3.79 | 1040.5556 | C_53_H_84_O_20_ | 1040.5508 | 1063.54 |
| 430 | Sumogaside | 3.79 | 1082.5509 | C_51_H_86_O_24_ | 1082.5525 | 1083.5598 |
| 431 | Picfeltarraenin IB | 3.8 | 489.26276 | C_29_H_35_N_3_O_4_ | 489.262 | 490.2693 |
| 432 | Evobioside | 3.8 | 1001.51099 | C_52_H_71_N_7_O_13_ | 1001.5087 | 1002.5159 |
| 433 | Delsemine A | 3.8 | 934.47734 | C_45_H_74_O_20_ | 934.4762 | 935.4835 |
| 434 | Microcystin YR | 3.81 | 532.34 | C_31_H_48_O_7_ | 532.3408 | 555.33 |
| 435 | Sedoheptulose | 3.81 | 499.29676 | C_26_H_45_NO_6_S | 499.2984 | 522.2877 |
| 436 | cis-Aconitic acid | 3.81 | 1044.55051 | C_52_H_84_O_21_ | 1044.5476 | 1067.5368 |
| 437 | 2-Methoxyanofinic acid | 3.81 | 536.29987 | C_30_H_40_N_4_O_5_ | 536.2974 | 581.2956 |
| 438 | Segetalin D | 3.82 | 814.41396 | C_44_H_62_O_14_ | 814.4163 | 815.4235 |
| 439 | 14-Methyl-hexadecanoic acid | 3.82 | 656.30977 | C_38_H_44_N_2_O_8_ | 656.3121 | 701.3103 |
| 440 | Glutamic acid | 3.83 | 709.40373 | C_37_H_59_NO_12_ | 709.4029 | 732.3922 |
| 441 | Kadsurenin B | 3.85 | 1088.57672 | C_54_H_88_O_22_ | 1088.5759 | 1111.5651 |
| 442 | Farnesyl acetate | 3.85 | 1067.54402 | C_54_H_73_N_11_O_12_ | 1067.541 | 1068.5483 |
| 443 | Tenacissoside K | 3.85 | 242.09027 | C_10_H_14_N_2_O_5_ | 242.0914 | 241.0841 |
| 444 | Astragaline A | 3.85 | 914.48752 | C_46_H_74_O_18_ | 914.4901 | 937.4794 |
| 445 | 1-Methyl-2-[(Z)-8-tetradecenyl]-4(1H)-quinolone | 3.85 | 204.08988 | C_11_H_12_N_2_O_2_ | 204.0906 | 205.0979 |
| 446 | Cimifoetiside Ⅴ | 3.85 | 152.09496 | C_8_H_12_N_2_O | 152.0958 | 197.094 |
| 447 | 2-Hydroxy-5-methoxy acetophenone | 3.86 | 1110.10332 | C_47_H_34_O_32_ | 1110.1046 | 1111.1118 |
| 448 | Julibroside C1 | 3.86 | 674.3091 | C_39_H_46_O_10_ | 674.3069 | 673.2996 |
| 449 | 5-O-Caffeoyl quinic acid butyl ester | 3.87 | 532.30362 | C_30_H_44_O_8_ | 532.3016 | 577.2998 |
| 450 | Hoodigoside E | 3.88 | 1004.51921 | C_49_H_80_O_21_ | 1004.5198 | 1027.5091 |
| 451 | Yemuoside YM11 | 3.9 | 1174.57712 | C_57_H_90_O_25_ | 1174.5817 | 1175.589 |
| 452 | Maltol | 3.9 | 870.4613 | C_44_H_70_O_17_ | 870.4654 | 893.4546 |
| 453 | Yemuoside YM13 | 3.9 | 1174.57712 | C_57_H_90_O_25_ | 1174.5724 | 1173.5651 |
| 454 | Nipponoside E | 3.91 | 1064.54034 | C_51_H_84_O_23_ | 1064.5372 | 1065.5444 |
| 455 | Nimbolidin E | 3.92 | 937.5035 | C_48_H_75_NO_17_ | 937.5024 | 960.4916 |
| 456 | 2-Ethyl-2-hexenoic aldehyde | 3.92 | 266.09429 | C_17_H_14_O_3_ | 266.0932 | 265.086 |
| 457 | Curculigo saponin E | 3.93 | 1082.52977 | C_54_H_82_O_22_ | 1082.5343 | 1081.527 |
| 458 | 6-N-Methyl adenosine | 3.94 | 526.25667 | C_30_H_38_O_8_ | 526.2586 | 571.2568 |
| 459 | Nipponoside B | 3.95 | 1072.54542 | C_53_H_84_O_22_ | 1072.5435 | 1095.5327 |
| 460 | Deoxyadenosine | 3.96 | 1194.60333 | C_57_H_94_O_26_ | 1194.6083 | 1195.6156 |
| 461 | Marsdekoiside B | 3.96 | 1030.53486 | C_51_H_82_O_21_ | 1030.5328 | 1029.5256 |
| 462 | Forsythoside B | 3.98 | 1044.52803 | C_52_H_72_N_10_O_13_ | 1044.526 | 1089.5242 |
| 463 | Tenacissoside C | 3.98 | 242.1089 | C_11_H_18_N_2_O_2_S | 242.109 | 287.1072 |
| 464 | Xanthiside | 3.99 | 896.47695 | C_46_H_72_O_17_ | 896.4802 | 919.4694 |
| 465 | Aesculioside G | 4.01 | 884.47695 | C_45_H_72_O_17_ | 884.4803 | 907.4696 |
| 466 | Senkirkine | 4.02 | 996.52938 | C_51_H_80_O_19_ | 996.5252 | 1019.5144 |
| 467 | Acantrifoside B | 4.02 | 509.29887 | C_27_H_43_NO_8_ | 509.2972 | 532.2864 |
| 468 | Anhalonine | 4.02 | 200.09496 | C_12_H_12_N_2_O | 200.0961 | 245.0943 |
| 469 | Delbruine | 4.03 | 1064.54034 | C_51_H_84_O_23_ | 1064.5377 | 1065.545 |
| 470 | Adenine | 4.04 | 802.43509 | C_40_H_66_O_16_ | 802.4321 | 803.4394 |
| 471 | Tenacissoside D | 4.06 | 242.1089 | C_11_H_18_N_2_O_2_S | 242.1085 | 287.1067 |
| 472 | Guanine | 4.08 | 592.36113 | C_33_H_52_O_9_ | 592.3616 | 615.3508 |
| 473 | Phenobarbitone | 4.1 | 1100.54034 | C_54_H_84_O_23_ | 1100.5398 | 1099.5325 |
| 474 | Thymol isobutyrate | 4.12 | 866.46639 | C_45_H_70_O_16_ | 866.4707 | 889.4599 |
| 475 | Phe | 4.12 | 1100.54034 | C_54_H_84_O_23_ | 1100.5435 | 1101.5507 |
| 476 | Curculigo saponin I | 4.12 | 1102.55599 | C_54_H_86_O_23_ | 1102.5553 | 1125.5445 |
| 477 | Acantrifoside C | 4.13 | 1120.60294 | C_55_H_92_O_23_ | 1120.6043 | 1143.5935 |
| 478 | 2α,3β-Dihydroxy nortropane | 4.14 | 826.43509 | C_42_H_66_O_16_ | 826.4359 | 849.4251 |
| 479 | Formyltanshinone | 4.2 | 1044.52803 | C_52_H_72_N_10_O_13_ | 1044.5247 | 1045.532 |
| 480 | Shionoside C | 4.24 | 668.37718 | C_35_H_56_O_12_ | 668.3767 | 691.366 |
| 481 | Macrostemonoside A | 4.27 | 764.43469 | C_41_H_64_O_13_ | 764.4313 | 787.4206 |
| 482 | 4a,6a-Dihydroxylycopodine | 4.29 | 188.10486 | C_9_H_16_O_4_ | 188.1054 | 187.0982 |
| 483 | Mulberrofuran P | 4.3 | 792.42961 | C_42_H_64_O_14_ | 792.4296 | 815.4188 |
| 484 | 11-Hydroxy-9-tridecenoic acid | 4.31 | 1078.5196 | C_51_H_82_O_24_ | 1078.519 | 1079.5263 |
| 485 | Curculigo saponin C | 4.34 | 1082.52977 | C_54_H_82_O_22_ | 1082.53 | 1083.5373 |
| 486 | 3-O-Formyl-20R,21-epoxyresibufogenin | 4.34 | 1018.49847 | C_49_H_78_O_22_ | 1018.5019 | 1019.5092 |
| 487 | Nimbolidin D | 4.34 | 896.47695 | C_46_H_72_O_17_ | 896.4737 | 919.4629 |
| 488 | Cistanoside B | 4.34 | 1102.5196 | C_53_H_82_O_24_ | 1102.5158 | 1101.5086 |
| 489 | Benzyl isothiocyanate | 4.36 | 1062.52469 | C_51_H_82_O_23_ | 1062.5271 | 1061.5198 |
| 490 | Benzaldehyde | 4.37 | 1062.52469 | C_51_H_82_O_23_ | 1062.5255 | 1063.5328 |
| 491 | Nicotine | 4.37 | 898.45622 | C_45_H_70_O_18_ | 898.4555 | 899.4628 |
| 492 | Glutathione oxidized | 4.41 | 709.40373 | C_37_H_59_NO_12_ | 709.4006 | 732.3898 |
| 493 | Ethyl glutamate | 4.41 | 908.48952 | C_47_H_68_N_6_O_12_ | 908.4887 | 931.4779 |
| 494 | Dracorhodin | 4.43 | 612.32983 | C_35_H_48_O_9_ | 612.3328 | 613.3401 |
| 495 | Kirenol | 4.43 | 824.44324 | C_41_H_60_N_8_O_10_ | 824.4459 | 847.4351 |
| 496 | Cyclo(Ala-Ile) | 4.44 | 1102.55599 | C_54_H_86_O_23_ | 1102.5596 | 1125.5488 |
| 497 | Poricoic acid DM | 4.45 | 1014.53995 | C_51_H_82_O_20_ | 1014.5444 | 1037.5336 |
| 498 | Sinomenine | 4.47 | 1098.56107 | C_55_H_86_O_22_ | 1098.5663 | 1121.5555 |
| 499 | Gentialutine | 4.47 | 1090.55599 | C_53_H_86_O_23_ | 1090.5557 | 1113.545 |
| 500 | Deoxyschizandrin | 4.48 | 1032.55051 | C_51_H_84_O_21_ | 1032.5538 | 1055.543 |
| 501 | Nimbolin B | 4.49 | 937.5035 | C_48_H_75_NO_17_ | 937.499 | 960.4882 |
| 502 | Fangchinoline | 4.5 | 1067.54402 | C_54_H_73_N_11_O_12_ | 1067.5429 | 1090.5321 |
| 503 | Tomatine | 4.5 | 906.48243 | C_44_H_74_O_19_ | 906.483 | 929.4723 |
| 504 | Sucrose | 4.5 | 1098.54582 | C_51_H_86_O_25_ | 1098.5466 | 1097.5393 |
| 505 | Acsonine | 4.52 | 802.43509 | C_40_H_66_O_16_ | 802.4332 | 825.4224 |
| 506 | Terpinen-4-ol acetate | 4.53 | 920.49808 | C_45_H_76_O_19_ | 920.4944 | 943.4837 |
| 507 | Cyclo-(Leu-Ile) | 4.54 | 200.10486 | C_10_H_16_O_4_ | 200.1056 | 199.0983 |
| 508 | Shionoside A | 4.6 | 1018.51373 | C_53_H_78_O_19_ | 1018.5112 | 1063.5094 |
| 509 | 3-Hexen-1-ol benzoate | 4.63 | 1018.49847 | C_49_H_78_O_22_ | 1018.498 | 1019.5053 |
| 510 | Deltaline | 4.63 | 936.49299 | C_45_H_76_O_20_ | 936.4933 | 981.4915 |
| 511 | Lycoposerramine O | 4.66 | 1104.57164 | C_54_H_88_O_23_ | 1104.5754 | 1127.5646 |
| 512 | Suspenol | 4.67 | 1096.53017 | C_51_H_84_O_25_ | 1096.5322 | 1097.5395 |
| 513 | Cynanoside Q2 | 4.68 | 357.19401 | C_21_H_27_NO_4_ | 357.1929 | 356.1857 |
| 514 | Oxymaistemonine | 4.69 | 750.41904 | C_40_H_62_O_13_ | 750.4175 | 773.4067 |
| 515 | Planteose | 4.69 | 854.46639 | C_44_H_70_O_16_ | 854.4671 | 877.4563 |
| 516 | Safrol | 4.7 | 940.53955 | C_49_H_80_O_17_ | 940.5424 | 985.5406 |
| 517 | Bavachinin | 4.72 | 1062.52469 | C_51_H_82_O_23_ | 1062.5266 | 1063.5339 |
| 518 | Cynanoside Q1 | 4.72 | 357.19401 | C_21_H_27_NO_4_ | 357.1929 | 356.1856 |
| 519 | Astragaline E | 4.75 | 914.48752 | C_46_H_74_O_18_ | 914.4879 | 937.4771 |
| 520 | Nopol | 4.76 | 858.46398 | C_45_H_62_N_8_O_9_ | 858.4646 | 881.4538 |
| 521 | Kaempferol 3-Lathyroside | 4.78 | 944.49808 | C_47_H_76_O_19_ | 944.5003 | 967.4895 |
| 522 | Safflomin C | 4.78 | 966.27937 | C_47_H_50_O_22_ | 966.2826 | 967.2898 |
| 523 | Kingianoside C | 4.83 | 824.44324 | C_41_H_60_N_8_O_10_ | 824.44 | 847.4292 |
| 524 | Aconitine | 4.94 | 802.43509 | C_40_H_66_O_16_ | 802.432 | 825.4212 |
| 525 | Esculentoside K | 5.12 | 1078.55599 | C_52_H_86_O_23_ | 1078.5597 | 1079.567 |
| 526 | Mandelonitrile | 5.13 | 884.47695 | C_45_H_72_O_17_ | 884.4751 | 907.4644 |
| 527 | Ganoderenic acid F | 5.18 | 392.19876 | C_25_H_28_O_4_ | 392.1973 | 391.19 |
| 528 | Pyrocatechol monoglucoside | 5.19 | 812.42211 | C_43_H_56_N_8_O_8_ | 812.4263 | 857.4245 |
| 529 | Esculentoside N | 5.3 | 312.12561 | C_13_H_20_N_4_O_3_S | 312.1251 | 311.1178 |
| 530 | Pregna-5,16-dien-3β-ol-20-one 3-O-α-L-rhamnopyranosyl-(1→2)-[α-L-rhamnopyranosyl-(1→4)]-β-D-glucopyranoside | 5.4 | 732.35096 | C_42_H_52_O_11_ | 732.3503 | 731.343 |
| 531 | 2-O-β-D-Glucopyranosylatractyligenin | 5.46 | 1088.54034 | C_53_H_84_O_23_ | 1088.5434 | 1089.5507 |
| 532 | Timosaponin F | 5.47 | 904.50317 | C_45_H_76_O_18_ | 904.507 | 949.5052 |
| 533 | Demecolcine | 5.48 | 950.50864 | C_46_H_78_O_20_ | 950.509 | 951.5163 |
| 534 | Pinnatifine I | 5.6 | 330.24062 | C_18_H_34_O_5_ | 330.2415 | 329.2342 |
| 535 | Ephedradine C | 5.9 | 224.14124 | C_13_H_20_O_3_ | 224.1425 | 269.1407 |
| 536 | Picrasinoside E | 5.95 | 330.24062 | C_18_H_34_O_5_ | 330.2415 | 329.2342 |
| 537 | Picrasinoside D | 6.02 | 330.24062 | C_18_H_34_O_5_ | 330.2414 | 329.2342 |
| 538 | Pilocarpine | 6.11 | 330.24062 | C_18_H_34_O_5_ | 330.241 | 329.2337 |
| 539 | Picrasinoside H | 6.19 | 330.24062 | C_18_H_34_O_5_ | 330.241 | 329.2337 |
| 540 | Picrasinoside G | 6.27 | 330.24062 | C_18_H_34_O_5_ | 330.2409 | 329.2337 |
| 541 | Atroposide G | 6.58 | 264.17254 | C_16_H_24_O_3_ | 264.1727 | 309.1709 |
| 542 | Leonurine | 6.59 | 281.14158 | C_18_H_19_NO_2_ | 281.1418 | 280.1346 |
| 543 | Eclalbasaponin Ⅴ | 6.61 | 248.17763 | C_16_H_24_O_2_ | 248.1777 | 293.1759 |
| 544 | Oxyberberine | 6.61 | 588.36622 | C_34_H_52_O_8_ | 588.3653 | 587.358 |
| 545 | Mahuannin D | 6.61 | 268.24023 | C_17_H_32_O_2_ | 268.2407 | 313.2389 |
| 546 | 4-Oxypentanoic acid | 6.62 | 264.20893 | C_17_H_28_O_2_ | 264.2102 | 309.2084 |
| 547 | Scroneoside A | 6.63 | 176.10486 | C_8_H_16_O_4_ | 176.1052 | 221.1034 |
| 548 | (10E,9S,12S,13S)-Trihydroxy-10-octadecenoate | 6.64 | 446.19407 | C_24_H_30_O_8_ | 446.1942 | 491.1924 |
| 549 | Atroposide H | 6.78 | 264.17254 | C_16_H_24_O_3_ | 264.1728 | 309.171 |
| 550 | 11,13-Dihydrosantamarine | 6.87 | 316.26136 | C_18_H_36_O_4_ | 316.2617 | 315.2545 |
| 551 | Stachyose | 6.92 | 196.14633 | C_12_H_20_O_2_ | 196.1462 | 195.1389 |
| 552 | Lucidenic acid D2 methyl ester | 6.93 | 295.15723 | C_19_H_21_NO_2_ | 295.1574 | 294.1502 |
| 553 | Ergonorine | 6.94 | 242.22458 | C_15_H_30_O_2_ | 242.2248 | 287.223 |
| 554 | SoyasaponinsⅠmethyl ester | 6.98 | 196.14633 | C_12_H_20_O_2_ | 196.1464 | 241.1446 |
| 555 | 7-O-Galloylcatechin | 7.05 | 149.08406 | C_9_H_11_NO | 149.0839 | 194.0821 |
| 556 | Lucidenic acid A | 7.11 | 295.15723 | C_19_H_21_NO_2_ | 295.1575 | 294.1502 |
| 557 | (25R)-5α-Spirostan-12-carbonyl-3-O-β-D-glucopyranosyl(1→2)-β-D-glucopysranosyl-(1→4)-β-D-galactopyranoside | 7.2 | 314.24571 | C_18_H_34_O_4_ | 314.2449 | 313.2377 |
| 558 | Galphin A | 7.33 | 616.17921 | C_30_H_32_O_14_ | 616.1777 | 615.1704 |
| 559 | 1,3-Dihydroxy-2-hydroxymethylanthraquinone-3-O-β-D-xylopyranose(1→6)-β-D-glucopyranoside | 7.44 | 316.26136 | C_18_H_36_O_4_ | 316.2616 | 315.2544 |
| 560 | 1,4-Diethyl-1,4-dimethyl-2,5-cyclohexadiene | 7.55 | 316.26136 | C_18_H_36_O_4_ | 316.2618 | 315.2545 |
| 561 | 1,3,6-Trihydroxy-2-methyl-anthraquinone-3-O-β-D-xylopyranose(1→6)-β-D-(6-O-acetyl)glucopyranoside | 7.61 | 316.26136 | C_18_H_36_O_4_ | 316.2617 | 315.2544 |
| 562 | Tyramine | 7.66 | 596.17412 | C_27_H_32_O_15_ | 596.1748 | 619.164 |
| 563 | 1,3,6-Trihydroxy-2-methylanthraquinone-3-O-(O-6-acetyl)-β-D-glucopyranoside | 7.71 | 316.26136 | C_18_H_36_O_4_ | 316.2617 | 315.2544 |
| 564 | Zearalenone | 7.91 | 318.14672 | C_18_H_22_O_5_ | 318.1472 | 317.14 |
| 565 | β-Rotunol | 8.14 | 234.16198 | C_15_H_22_O_2_ | 234.1624 | 235.1697 |
| 566 | Periplocoside M | 8.32 | 298.25079 | C_18_H_34_O_3_ | 298.2513 | 297.244 |
| 567 | Citric acid | 8.35 | 342.14672 | C_20_H_22_O_5_ | 342.1463 | 365.1355 |
| 568 | Clinopodiside B | 8.38 | 338.24571 | C_20_H_34_O_4_ | 338.2457 | 361.2349 |
| 569 | Periplocoside L | 8.45 | 298.25079 | C_18_H_34_O_3_ | 298.2516 | 297.2443 |
| 570 | Proline | 8.63 | 348.17842 | C_16_H_28_O_8_ | 348.1795 | 347.1722 |
| 571 | Hoodigoside K | 8.71 | 254.26097 | C_17_H_34_O | 254.2617 | 299.2599 |
| 572 | Hoodigoside M | 8.8 | 254.26097 | C_17_H_34_O | 254.2613 | 299.2595 |
| 573 | Cireneol G | 9.15 | 482.26684 | C_29_H_38_O_6_ | 482.266 | 481.2587 |
| 574 | 3-Acetylaconitine | 9.16 | 826.43509 | C_42_H_66_O_16_ | 826.4338 | 827.4411 |
| 575 | Hypoxanthine | 9.16 | 424.20972 | C_22_H_32_O_8_ | 424.2078 | 425.215 |
| 576 | Adenosine diphosphate | 9.16 | 418.19915 | C_23_H_30_O_7_ | 418.2003 | 441.1895 |
| 577 | Ophiopogonin E | 9.16 | 272.0896 | C_12_H_16_O_7_ | 272.0904 | 273.0977 |
| 578 | Methyl propyl disulfide | 9.3 | 546.37091 | C_36_H_50_O_4_ | 546.3691 | 591.3673 |
| 579 | Hypaconitine | 9.41 | 424.20972 | C_22_H_32_O_8_ | 424.2081 | 425.2154 |
| 580 | Uridine | 9.41 | 406.27192 | C_24_H_38_O_5_ | 406.2722 | 429.2614 |
| 581 | Methyl protogracillin | 9.45 | 546.37091 | C_36_H_50_O_4_ | 546.3696 | 591.3678 |
| 582 | MFCD03296125 | 9.55 | 546.37091 | C_36_H_50_O_4_ | 546.3695 | 591.3677 |
| 583 | α-Onocerin | 9.55 | 442.38108 | C_30_H_50_O_2_ | 442.3812 | 465.3704 |
| 584 | Hypoglaucin G | 9.79 | 424.20972 | C_22_H_32_O_8_ | 424.2082 | 425.2155 |
| 585 | Cynanoside R2 | 9.79 | 280.24023 | C_18_H_32_O_2_ | 280.2414 | 279.2341 |
| 586 | Apoatropine | 9.79 | 404.25627 | C_24_H_36_O_5_ | 404.2559 | 427.2451 |
| 587 | L-(+)-Valine | 9.79 | 166.13577 | C_11_H_18_O | 166.1365 | 211.1347 |
| 588 | Microcystin WR | 9.85 | 546.37091 | C_36_H_50_O_4_ | 546.37 | 591.3682 |
| 589 | Diphenylamine | 9.91 | 834.47656 | C_45_H_70_O_14_ | 834.4805 | 879.4787 |
| 590 | Sachunoside | 9.96 | 472.31887 | C_29_H_44_O_5_ | 472.3209 | 473.3282 |
| 591 | Microcystin LA | 9.97 | 546.37091 | C_36_H_50_O_4_ | 546.3699 | 591.3681 |
| 592 | Norswertianolin | 9.97 | 858.46398 | C_45_H_62_N_8_O_9_ | 858.4662 | 903.4644 |
| 593 | Disinomenine | 9.97 | 834.47656 | C_45_H_70_O_14_ | 834.4793 | 879.4775 |
| 594 | (25R)-Spirostan-4-ene-3,6,12-trione | 9.98 | 178.13577 | C_12_H_18_O | 178.1365 | 223.1347 |
| 595 | Kaurane acid glycoside A | 10 | 335.31881 | C_22_H_41_NO | 335.3199 | 380.3181 |
| 596 | Mudanpioside H | 10.08 | 810.44017 | C_42_H_66_O_15_ | 810.4402 | 811.4474 |
| 597 | Microcystin LF | 10.09 | 546.37091 | C_36_H_50_O_4_ | 546.37 | 591.3682 |
| 598 | Hydroxyvalerenic acid | 10.13 | 424.20972 | C_22_H_32_O_8_ | 424.2085 | 425.2158 |
| 599 | Methyl pyrophaeophorbide а | 10.2 | 546.37091 | C_36_H_50_O_4_ | 546.3695 | 591.3677 |
| 600 | Norleucine | 10.21 | 858.46398 | C_45_H_62_N_8_O_9_ | 858.4644 | 903.4626 |
| 601 | Methyl jasmonate | 10.31 | 546.37091 | C_36_H_50_O_4_ | 546.3692 | 591.3674 |
| 602 | Nornuciferine | 10.31 | 858.46398 | C_45_H_62_N_8_O_9_ | 858.4642 | 903.4624 |
| 603 | Mirificin | 10.35 | 810.44017 | C_42_H_66_O_15_ | 810.4424 | 811.4497 |
| 604 | 14-Hydroxy sprengerinin C | 10.51 | 169.08915 | C_12_H_11_N | 169.0896 | 170.0968 |
| 605 | 14-epi-Andrographolide | 11.79 | 169.08915 | C_12_H_11_N | 169.0894 | 170.0967 |
| 606 | Mulberrofuran A | 12.14 | 810.44017 | C_42_H_66_O_15_ | 810.4413 | 811.4486 |
| 607 | Neotigogenin-3-O-β-D-xylopyranosyl(1→2)-[β-D-xylopyranosyl (1→3)]-β-D-glucopy-ranosyl(1→4)-[α-L-rhamnopyranosyl (1→2)]-β-D-galactopyranoside | 12.52 | 528.34509 | C_32_H_48_O_6_ | 528.3427 | 551.3319 |
| 608 | Neociwujiaphenol | 12.97 | 612.27819 | C_30_H_44_O_13_ | 612.281 | 657.2792 |
| 609 | β-Sitosteryl tetra-O-acetyl-β-D-glycopyranoside | 13.23 | 744.48125 | C_43_H_68_O_10_ | 744.4778 | 767.467 |
| 610 | Methyl 2α, 3β-diacetyloxy-lup-20(29)-en-28-oate | 13.24 | 745.56216 | C_41_H_80_NO_8_P | 745.5631 | 768.5523 |
| 611 | Methionine | 13.49 | 745.56216 | C_41_H_80_NO_8_P | 745.5618 | 768.5511 |
| 612 | Lycoposseramine M | 13.69 | 790.52311 | C_45_H_74_O_11_ | 790.5217 | 789.5145 |
| 613 | Methyl myristate | 13.78 | 546.37091 | C_36_H_50_O_4_ | 546.3683 | 591.3665 |
| 614 | Maltose | 13.8 | 870.4613 | C_44_H_70_O_17_ | 870.4589 | 869.4516 |
| 615 | Astin B | 13.83 | 878.50277 | C_47_H_74_O_15_ | 878.5037 | 877.4965 |
| 616 | Dendrodensiflorol | 13.83 | 950.50864 | C_46_H_78_O_20_ | 950.511 | 995.5092 |
| 617 | Nor-ψ-tropine | 13.84 | 858.46398 | C_45_H_62_N_8_O_9_ | 858.4618 | 903.46 |
| 618 | Thymidine | 13.84 | 866.46639 | C_45_H_70_O_16_ | 866.4681 | 889.4573 |
| 619 | Saucernetin-8, | 13.84 | 342.11621 | C_12_H_22_O_11_ | 342.1167 | 341.1095 |
| 620 | Pantothenic acid | 13.86 | 704.41356 | C_39_H_60_O_11_ | 704.4129 | 727.4021 |
| 621 | Microcystin LY | 13.91 | 546.37091 | C_36_H_50_O_4_ | 546.3684 | 591.3666 |
|  | Ecdysterone-3-O-β-D-glucopyranoside | 13.92 | 570.39204 | C_35_H_54_O_6_ | 570.3923 | 593.3815 |
| 623 | Adenosine | 14.01 | 802.43509 | C_40_H_66_O_16_ | 802.4322 | 847.4304 |
| 624 | Styrene | 14.02 | 754.41396 | C_39_H_62_O_14_ | 754.4108 | 755.4181 |
| 625 | Ganosporeric acid A | 14.02 | 238.22967 | C_16_H_30_O | 238.2302 | 239.2375 |
| 626 | Aduncin | 14.02 | 518.19407 | C_30_H_30_O_8_ | 518.1923 | 519.1996 |
| 627 | Muscone | 14.02 | 494.19541 | C_29_H_26_N_4_O_4_ | 494.1957 | 495.2029 |
|  |  |  |  |  |  |  |
